# Mitotic checkpoint gene expression is tuned by codon usage bias

**DOI:** 10.15252/embj.2021107896

**Published:** 2022-07-11

**Authors:** Eric Esposito, Douglas E Weidemann, Jessie M Rogers, Claire M Morton, Erod Keaton Baybay, Jing Chen, Silke Hauf

**Affiliations:** ^1^ Department of Biological Sciences Virginia Tech Blacksburg VA USA; ^2^ Fralin Life Sciences Institute Virginia Tech Blacksburg VA USA

**Keywords:** co‐translational assembly, gene expression noise, mitosis, mRNA decay, spindle assembly checkpoint, Cell Cycle, RNA Biology, Translation & Protein Quality

## Abstract

The mitotic checkpoint (also called spindle assembly checkpoint, SAC) is a signaling pathway that safeguards proper chromosome segregation. Correct functioning of the SAC depends on adequate protein concentrations and appropriate stoichiometries between SAC proteins. Yet very little is known about the regulation of SAC gene expression. Here, we show in the fission yeast *Schizosaccharomyces pombe* that a combination of short mRNA half‐lives and long protein half‐lives supports stable SAC protein levels. For the SAC genes *mad2* ^+^ and *mad3* ^+^, their short mRNA half‐lives are caused, in part, by a high frequency of nonoptimal codons. In contrast, *mad1* ^+^ mRNA has a short half‐life despite a higher frequency of optimal codons, and despite the lack of known RNA‐destabilizing motifs. Hence, different SAC genes employ different strategies of expression. We further show that Mad1 homodimers form co‐translationally, which may necessitate a certain codon usage pattern. Taken together, we propose that the codon usage of SAC genes is fine‐tuned to ensure proper SAC function. Our work shines light on gene expression features that promote spindle assembly checkpoint function and suggests that synonymous mutations may weaken the checkpoint.

## Graphical abstract

Optimal protein levels of the mitotic checkpoint proteins Mad1, Mad2, and Mad3 in fission yeast are the result of a combination of differential regulation of mRNA and protein stability.

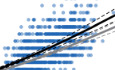

## Introduction

The spindle assembly checkpoint (SAC; also called mitotic checkpoint) is a eukaryotic signaling pathway that delays cell cycle progression when chromosomes have not yet become properly attached to microtubules during mitosis (Lara‐Gonzalez *et al*, 2012; Musacchio, 2015; Kops *et al*, 2020). Proper function of the SAC needs appropriate SAC protein concentrations (both too low and too high expression can be detrimental) and needs adequate stoichiometries between proteins in the pathway (Chung & Chen, 2002; Ryan *et al*, 2012; Schuyler *et al*, 2012; Heinrich *et al*, 2013; Gross *et al*, 2018). This makes it important to quantitatively understand SAC gene expression. Yet, the expression of these genes has not been studied in any detail.

**Figure 1:**
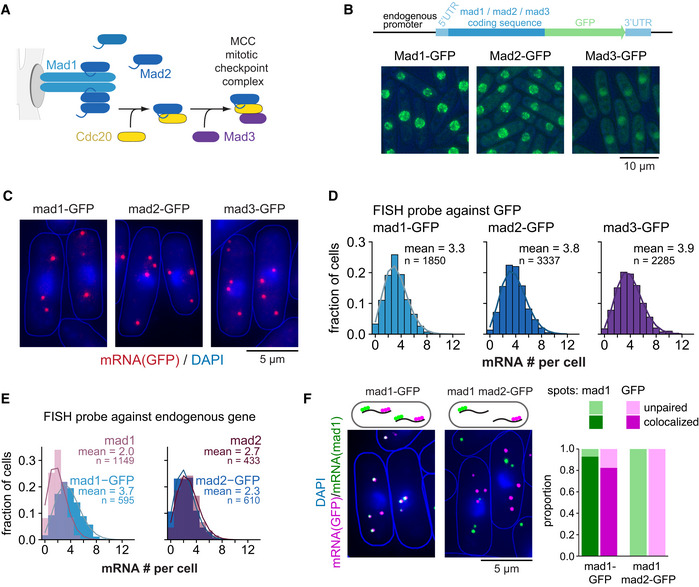
Low steady‐state mRNA numbers of checkpoint genes *mad1* ^+^, *mad2* ^+^, and *mad3* ^+^ AOverview of the interactions between Mad1, Mad2, and Mad3.BSchematic of marker‐less GFP‐tagging at the endogenous locus and representative live‐cell images of Mad1‐, Mad2‐, and Mad3‐GFP strains (average intensity projections).CRepresentative images of single‐molecule mRNA FISH (smFISH) staining of *S. pombe* using probes against GFP (red). DNA was stained with DAPI (blue). The gamma‐value was adjusted to make the cytoplasm visible; cell shapes are outlined in blue.DFrequency distribution of mRNA numbers per cell determined by smFISH; combined data from 3, 4, and 5 experiments, respectively, shown separately in Fig EV1C; *n*, number of cells. Curves show fit to a Poisson distribution.EFrequency distribution of mRNA numbers per cell using FISH probes against the endogenous genes and using either strains expressing the GFP‐tagged gene or the endogenous, untagged gene. Curves show fit to a Poisson distribution. The difference for *mad1* ^+^ is statistically significant, that for *mad2* ^+^ is not (Fig EV1E). A lower mRNA number for untagged *mad1* ^+^ was also observed in an independent strain.FCo‐staining by smFISH using probes against *mad1* ^+^ and GFP either in a strain expressing *mad1* ^+^‐GFP as a positive control or in a strain expressing wild‐type *mad1* ^+^ and *mad2* ^+^‐GFP. Cytoplasmic *mad1* ^+^ (green) or GFP mRNA spots (magenta) were quantified as co‐localizing or not with the respective other. For the *mad1* ^+^‐GFP strain, 544 cells and a total of 1,641 mad1 spots and 1,839 GFP spots were analyzed; 48 cells were not considered as they did not contain at least one spot of each type in the cytoplasm. For the *mad1* ^+^ *mad2* ^+^‐GFP strain, 571 cells and a total of 1,107 mad1 spots and 1,537 GFP spots were analyzed; 158 cells were not considered since they did not contain at least one spot of each type in the cytoplasm. Source data are available online for this figure.

The protein network of the SAC, on the other hand, is well understood. While the SAC is active, it forms the mitotic checkpoint complex (MCC), which prevents the anaphase‐promoting complex (APC/C) from initiating anaphase (Pines, 2011). A key effector of the SAC is the Mad1/Mad2 complex, a tetramer of two Mad1 and two Mad2 molecules (Chen *et al*, 1999; Sironi *et al*, 2002; Fig 1A). Mad1 homodimerizes through a long, parallel intermolecular coiled‐coil at its N‐terminus, which is followed by the Mad2‐binding motif and a C‐terminal RWD (RING finger‐, WD‐repeat‐, and DEAD‐like proteins) domain (Chen *et al*, 1999; Sironi *et al*, 2002; Kim *et al*, 2012; Piano *et al*, 2021; preprint: Fischer *et al*, 2022). The Mad1‐binding partner Mad2 is a HORMA domain protein (named after Hop1, Rev7, and Mad2) that can change its conformation between open (O) and closed (C) (Aravind & Koonin, 1998; Luo *et al*, 2002, 2004). To bind Mad1, the C‐terminus of Mad2 wraps around the Mad1 polypeptide similar to a seat belt and Mad2 adopts the closed conformation (Luo *et al*, 2002; Sironi *et al*, 2002). This results in a tight complex with no measurable dissociation rate *in vitro* (Chen *et al*, 1999; Sironi *et al*, 2001; Vink *et al*, 2006). If and to what extent the formation of the intricate Mad1/Mad2 complex is aided by other factors is unknown.

Through a different surface, Mad2 can form heterodimers between its two conformations (O‐C) (Mapelli *et al*, 2007). Dimerization of Mad1/C‐Mad2 with O‐Mad2 facilitates binding of this O‐Mad2 molecule to the APC/C activator Cdc20 (Slp1 in *Schizosaccharomyces pombe*) (De Antoni *et al*, 2005; Piano *et al*, 2021; preprint: Fischer *et al*, 2022). O‐Mad2 changes its conformation in the process, forming C‐Mad2/Cdc20 through the same seat belt type of binding (Luo *et al*, 2002). Subsequent binding of BubR1 (Mad3 in yeast) to C‐Mad2/Cdc20 results in the mitotic checkpoint complex (MCC) (Sudakin *et al*, 2001; Chao *et al*, 2012). The MCC then inhibits the APC/C to block anaphase (Pines, 2011; Alfieri *et al*, 2016).

Because the SAC plays a central role in preventing chromosome mis‐segregation and because persistent chromosome mis‐segregation is a driver of tumor evolution, SAC malfunction is suspected to contribute to carcinogenesis (Gordon *et al*, 2012; Funk *et al*, 2016). Mouse models have shown that impairing the SAC promotes chromosome mis‐segregation and tumor formation (Baker *et al*, 2005; Holland & Cleveland, 2009; Schvartzman *et al*, 2010). Completely abolishing the SAC, however, is detrimental to human cells (Dobles *et al*, 2000; Kops *et al*, 2004; Michel *et al*, 2004; Schukken *et al*, 2021), and suppression of the SAC may in fact be a successful therapeutic strategy against some cancer types (Cohen‐Sharir *et al*, 2021; Quinton *et al*, 2021). Together, these results indicate that tuning SAC function can make the difference between normal growth, cancerous growth, and cell death.

Although the SAC network has been studied in much detail from a protein‐centric view, little is known about SAC gene expression. Understanding this regulatory layer is important, because the changes in SAC protein concentrations can cause SAC malfunction—at least partly because proper stoichiometries, such as between Mad1 and Mad2, are important for function (Chung & Chen, 2002; Ryan *et al*, 2012; Schuyler *et al*, 2012; Heinrich *et al*, 2013; Gross *et al*, 2018). Here, using fission yeast (*Schizosaccharomyces pombe*), we study the mRNA layer of SAC gene expression and provide evidence that a combination of short mRNA and long protein half‐lives ensures a stable concentration of SAC proteins over time and between cells. Our findings indicate that codon usage bias in *mad2* ^+^ and *mad3* ^+^, but not *mad1* ^+^, contributes to their short mRNA half‐lives, and that the coding sequence of *mad1* ^+^ carries other features that influence expression of this gene. We provide evidence that Mad1 homodimers form co‐translationally, which may necessitate a certain codon usage pattern. Overall, our findings shine light on gene expression features that promote SAC function and raise the possibility that synonymous mutations may impair the SAC.

## Results

### SAC mRNA numbers are approximately Poisson‐distributed with means of two to four per cell

**Figure EV1:**
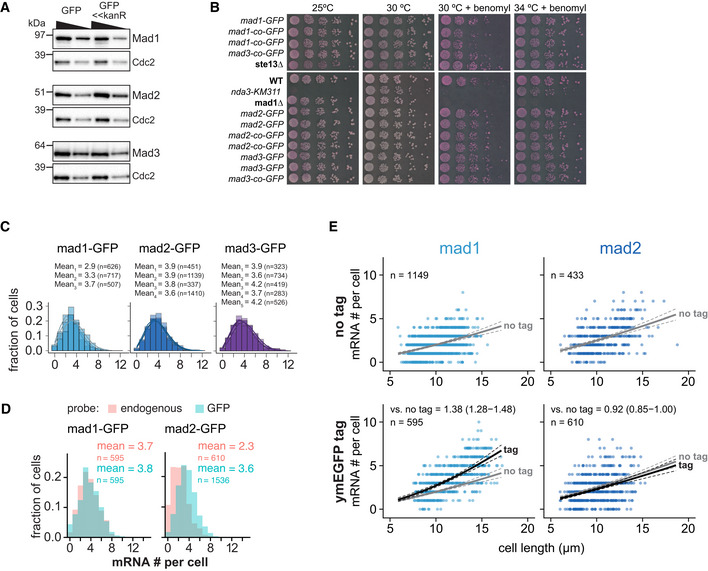
Additional data on Mad1, Mad2, and Mad3 tagging and mRNA numbers AImmunoblot comparing expression of *mad1* ^+^, *mad2* ^+^, and *mad3* ^+^ tagged at the endogenous locus either by marker‐less insertion of yeast codon‐optimized monomeric enhanced GFP (ymEGFP, here: GFP) or conventionally with GFP‐S65T and a kanamycin‐resistance cassette (GFP<<kanR). Antibodies against the endogenous proteins were used. Cdc2 was probed as loading control. A 1:1 dilution is loaded in the second lane for each sample.BGrowth assay for the indicated strains on rich medium plates without (left side) or with benomyl (right side). The agar contains Phloxine B, which stains dead cells.CFrequency distribution of mRNA numbers per cell. Data from individual experiments which are shown combined in Fig 1. Probes were against the GFP portion of each fusion gene. Curves show fit to a Poisson distribution.DFrequency distribution of mRNA numbers per cell using probes against the endogenous gene or against GFP in strains expressing a GFP fusion of either *mad1* ^+^ or *mad2* ^+^. The comparison illustrates that for *mad2* ^+^‐GFP either the endogenous probe is less sensitive, or there is considerable mRNA degradation from the 5′ end leading to fewer detected spots with a probe on the endogenous gene than on the 3′ end GFP tag.ESame experiment as Fig 1E. Scatter plots of whole‐cell mRNA counts versus cell length. Solid lines are regression curves from generalized linear mixed model fits (gray for no tag, black for GFP‐tagged gene). Dashed lines represent 95% bootstrap confidence bands for the regression curves. Model estimates of the ratio of tagged to untagged mRNA levels with bootstrap 95% confidence intervals are included in the plots. One experiment with probes against *mad1* ^+^ or *mad2* ^+^ coding sequences, respectively. Source data are available online for this figure.

We previously quantified the concentration of SAC proteins fused to green fluorescent protein (GFP) in *S. pombe* and determined protein concentrations in a range between 30 and 150 nM with strikingly little intercell variability (i.e., low gene expression “noise”) (Heinrich *et al*, 2013). In these strains, GFP had been fused by traditional tagging, changing the endogenous 3′ UTR to that of the *Saccharomyces cerevisiae ADH1* gene and appending an antibiotic‐resistance gene, which both may alter gene expression. To avoid such effects, we now employed CRISPR/Cas9‐mediated scarless genome editing (Jacobs *et al*, 2014). We fused ymEGFP (yeast codon‐optimized, monomeric enhanced GFP; in the following just “GFP”) to the SAC genes *mad1* ^+^, *mad2* ^+^, and *mad3* ^+^ without any change to the surrounding sequences (Fig 1B). Immunoblots showed concentrations broadly similar to the previous strains (Fig EV1A), and strains were not sensitive to the microtubule drug benomyl, suggesting that SAC functionality was maintained (Fig EV1B).

The mean SAC mRNA numbers per cell, determined by single‐molecule mRNA fluorescence *in situ* hybridization (FISH) with probes targeting GFP, were in the range of 3 to 4, even lower than the means of 4.5 to 6 that we had previously observed (Figs 1C and D, and EV1C; Heinrich *et al*, 2013). This indicates that the traditional tagging strategy indeed influenced gene expression. To test whether the expression in the new strains resembles endogenous expression, we used FISH probes against endogenous *mad1* ^+^ and *mad2* ^+^ and compared strains expressing the endogenous untagged gene with strains expressing the GFP‐tagged gene. For *mad2* ^+^, the mean mRNA number for untagged and tagged *mad2* ^+^ was comparable (Figs 1E and EV1E). However, untagged *mad1* ^+^ showed even fewer mRNA molecules than *mad1* ^+^‐GFP (Fig 1E and EV1E), suggesting that the mere addition of GFP, without any changes in the UTRs or surrounding sequences can change expression of *mad1* ^+^. [Note that for *mad2* ^+^, the efficiency of the gene‐specific probe was slightly lower than the GFP probe (Fig EV1D, both probes measured on *mad2* ^+^‐GFP), but this is not expected to influence the conclusion in an experiment that only uses the gene‐specific probe (Fig 1E).

While the mean mRNA numbers per cell for the GFP‐tagged genes were in the range of 3 to 4, the numbers in single cells ranged from 0 to around 9 (Fig 1D and E). As expected (Zhurinsky *et al*, 2010; Padovan‐Merhar *et al*, 2015; Sun *et al*, 2020), smaller cells had on average lower numbers than larger cells (Fig EV1E). However, even cells of the same size could differ in mRNA number by 8 or more (Fig EV1E). The spread of mRNA numbers in the cell population was well approximated by a Poisson distribution (Fig 1D and E). A Poisson distribution is expected from constitutive expression, where mRNA is synthesized and degraded in uncorrelated events but with a uniform probability over time. In contrast, “bursty” expression (characterized by alterations of promoter activity and inactivity) would result in an even wider distribution (Zenklusen *et al*, 2008). These results therefore indicate that SAC mRNA numbers vary considerably, but that this variation is within the expected range for constitutive expression.

### *mad1* ^+^ and *mad2* ^+^ mRNAs do not co‐localize in the cytoplasm

The mRNA FISH data also provide the location of mRNAs. Recent work has suggested that co‐translational assembly of protein complexes is more prevalent than previously thought (Schwarz & Beck, 2019). How the stable Mad1/Mad2 complex assembles is unknown. When heterodimeric complexes assemble while both subunits are being translated, their mRNAs will co‐localize (Panasenko *et al*, 2019). We asked whether this is the case for Mad1 and Mad2. We stained *mad1* ^+^ mRNA (using a *mad1* ^+^ probe) and *mad2* ^+^‐GFP mRNA (using a GFP probe) in the same cells, where both were expressed from their respective endogenous loci. While a *mad1* ^+^‐GFP strain, used as positive control, showed strong co‐localization of the *mad1* ^+^ and GFP probes, there was no evidence for co‐localization of *mad1* ^+^ and *mad2* ^+^‐GFP mRNA (Fig 1F). This absence of mRNA co‐localization excludes that the Mad1/Mad2 complex forms by synchronous co‐translational assembly. We will discuss other possibilities below.

### Low protein noise can be explained through long protein and short mRNA half‐lives

**Figure 2:**
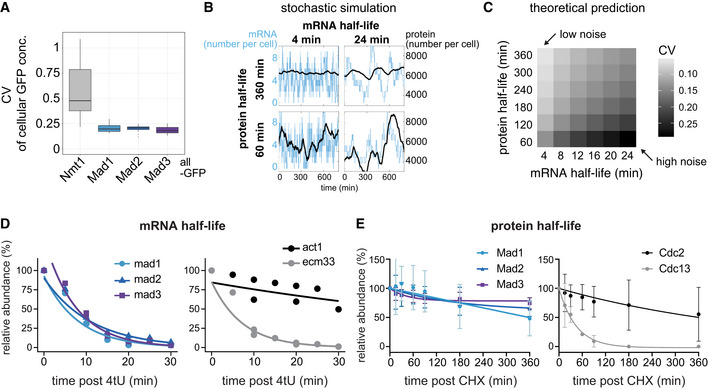
The checkpoint genes *mad1* ^+^, *mad2* ^+^, and *mad3* ^+^ combine short mRNA and long protein half‐lives, explaining low noise ACellular protein noise (coefficient of variation, CV = std / mean) in live‐cell microscopy images of *S. pombe*; *n* = 7 images (Nmt1‐GFP), 11 (Mad1‐GFP), 19 (Mad2‐GFP), 10 (Mad3‐GFP); single images had 16–79 GFP‐positive and 6–94 GFP‐negative (control) cells. Boxplots show median and interquartile range (IQR); whiskers extend to values no further than 1.5 times the IQR from the first and third quartile, respectively. Mad1, Mad2, and Mad3 all showed significantly lower noise than Nmt1 (Wilcoxon rank sum test; all *P* < 0.001).BSimulations of stochastic gene expression noise from selected mRNA/protein half‐life combinations assuming a constantly active promoter (see Methods). Synthesis rates were set to obtain a mean mRNA number of 4 per cell, and a mean protein number of 6,000 per cell. The x‐axis of each graph shows time, the y‐axis shows mRNA number per cell (blue) or protein number per cell (black).CTheoretical prediction for the coefficient of variation (CV = std/mean) of the protein number per cell, assuming different mRNA and protein half‐lives, using the same underlying model as in B. Synthesis rates were adjusted to maintain a mean mRNA number per cell of 3.5, and a mean protein number per cell of 6,000 (approx. 100 nM).DmRNA abundances by qPCR following metabolic labeling and removal of the labeled pool (two independent experiments). Lines are regression curves from generalized linear mixed model fits, excluding the measurements at *t* = 0 in order to accommodate for noninstantaneous labeling by 4tU. Act1^+^ and ecm33^+^ were used as long and short half‐life controls, respectively; qPCR was performed for the endogenous mRNAs. Half‐lives (95% confidence interval): *mad1* ^+^ 5.6 min (4.3–8.4), *mad2* ^+^ 7.7 min (6.2–10.4), *mad3* ^+^ 5.2 min (4.3–6.9), act1^+^ 61.8 min (37.2–172.3), ecm33^+^ 5.0 min (4.5–5.7).EProtein abundances after translation shut‐off with cycloheximide (CHX); *n* = 3 experiments, error bars = std. Lines indicate fit to a one‐phase exponential decay. Cdc2 and Cdc13 were used as long and short half‐life controls, respectively. Immunoblots for the endogenous proteins (no tag). A representative experiment shown in Appendix Fig S2E. Source data are available online for this figure.

To analyze if and to what extent the strong mRNA variation propagates to the protein level, we quantified GFP‐tagged Mad1, Mad2, and Mad3 in single cells using our “Pomegranate” image analysis pipeline, which allows for 3D segmentation (Appendix Fig S1 and S2A; Baybay *et al*, 2020). To subtract autofluorescence, we mixed the GFP‐expressing cells with cells not expressing GFP (Appendix Fig S1). Unlike for the mRNA, we observed little cell‐to‐cell variability in the SAC protein concentrations (Fig 2A). As a comparison, we imaged a “noisy” *S. pombe* protein, Nmt1 (Saint *et al*, 2019), which indeed showed pronounced cell‐to‐cell variability (Fig 2A; Appendix Fig S1C). A measure of variability is the coefficient of variation (CV; standard deviation divided by mean). The CVs for Mad1‐, Mad2‐, or Mad3‐GFP were in the range of 0.2, whereas that for Nmt1‐GFP was around 0.5 (Fig 2A).

This raised the question how the protein concentrations of Mad1, Mad2, and Mad3 can be homogeneous across the population when the mRNA numbers are highly variable. We considered a simple gene expression model with a constitutively active promoter, and different mRNA and protein synthesis and degradation rates (see Methods for details) that would all yield mean mRNA and protein numbers similar to those that we observe for *mad1* ^+^, *mad2* ^+^, and *mad3* ^+^. The longer the mRNA half‐life, the longer a state of low or high mRNA numbers persists; and the shorter the protein half‐life, the more closely protein concentrations follow the mRNA numbers (Fig 2B). Hence, long mRNA half‐lives and short protein half‐lives favor noise, whereas short mRNA half‐lives and long protein half‐lives suppress noise (Fig 2B and C; Appendix Fig S2B). In the latter case, the long persistence time of proteins buffers fast fluctuations at the mRNA level (Fig 2B).

To ascertain whether this prediction is met by SAC genes, we measured mRNA and protein half‐lives. We determined mRNA half‐life by metabolic labeling followed by depletion of the labeled pool and quantification of the remaining pool by quantitative PCR. The mRNA half‐lives for *mad1* ^+^, *mad2* ^+^, and *mad3* ^+^ were all in the range of a few minutes (*mad1* ^+^: 5.6 min, *mad2* ^+^: 7.7 min, and *mad3* ^+^: 5.2 min) (Fig 2D). This was consistent with the half‐lives determined for these genes in a large‐scale study using metabolic labeling (Appendix Fig S2D) (Eser *et al*, 2016). RNA half‐lives have been notoriously difficult to measure, with much variability between studies (Carneiro *et al*, 2019; preprint: Agarwal & Kelley, 2022). An earlier *S. pombe* study (Hasan *et al*, 2014) found longer half‐lives across the entire transcriptome, but even in this study, SAC genes were at the lower end of mRNA half‐lives (Appendix Fig S2D). As controls, we measured two unrelated genes with reportedly long and short half‐life (Eser *et al*, 2016), *act1* ^+^ and *ecm33* ^+^, which behaved as expected (Fig 2D). We determined protein half‐lives by translation shut‐off using cycloheximide, followed by immunoblotting. The half‐lives of Mad1, Mad2, and Mad3 were in the range of many hours, considerably longer than the typical *S. pombe* cell cycle of 2.5 h (Fig 2E; Appendix Fig S2E) and broadly consistent with previous data (Sczaniecka *et al*, 2008; Horikoshi *et al*, 2013; Christiano *et al*, 2014). This large difference in mRNA and protein half‐lives explains the low cell‐to‐cell variability in protein concentration despite the considerable variation in mRNA numbers (Fig 2C). The short mRNA half‐life is therefore important to mitigate the effect of the large variation in mRNA numbers.

### *mad2* ^+^ and *mad3* ^+^ have low codon stabilization coefficients

**Figure 3:**
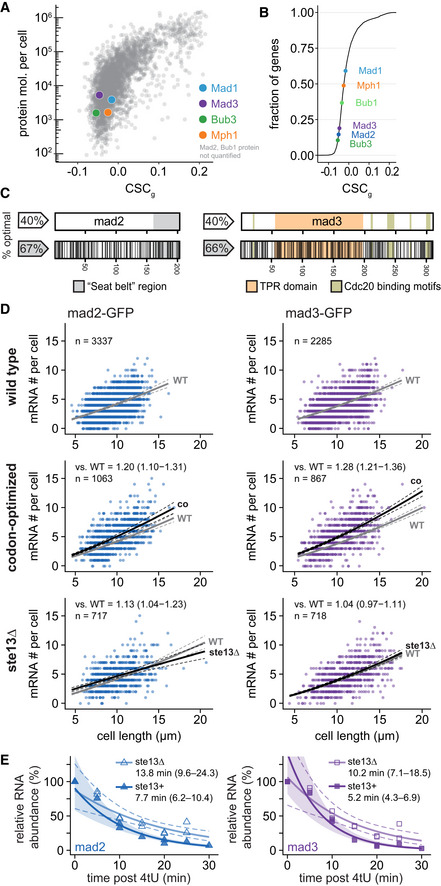
Codon‐optimization increases the steady‐state mRNA numbers of mad2 and mad3 AThe mean CSC value for each *S. pombe* gene (CSC_g_) relative to protein number per cell by mass spectrometry (Carpy *et al*, 2014). CSC was determined using the mRNA half‐life data by Eser *et al* (2016) as described in Methods. Colored dots highlight proteins of interest. For Mad2 and Bub1, no protein abundance data was available.BCumulative frequency distribution of the CSC_g_ values for protein‐coding *S. pombe* genes. The position of spindle assembly checkpoint genes is highlighted.CSchematic of the *mad2* ^+^ and *mad3* ^+^ genes. Regions coding for important structural features are highlighted. Black lines in the bottom graph indicate synonymous codon changes in the codon‐optimized version.DScatter plots of whole‐cell RNA counts versus cell length. Solid lines are regression curves from generalized linear mixed model fits (gray: wild type, black: codon‐optimized or *ste13Δ*). Dashed lines: 95% bootstrap confidence bands for the regression curves. Model estimates of the ratio relative to wild‐type mRNA are included with bootstrap 95% confidence interval in brackets. Two to five replicates per genotype.ETime course of RNA abundances by qPCR following metabolic labeling and removal of the labeled pool (two independent experiments). Solid lines: regression curves from generalized linear mixed model fits (dark = *ste13* ^+^, light = *ste13Δ*), excluding *t* = 0 to accommodate for non‐instantaneous labeling by 4tU. Shaded area: 95% bootstrap confidence band for *ste13* ^+^; dashed lines: 95% bootstrap confidence band for *ste13Δ*. Half‐life estimates are included with 95% bootstrap confidence intervals in brackets. See Fig EV2C for additional statistics. The *ste13* ^+^ data are the same as in Fig 2. Source data are available online for this figure.

One of the determining factors for mRNA half‐life is codon optimality, which positively correlates with mRNA stability in several eukaryotes (Presnyak *et al*, 2015; Hanson & Coller, 2018; Narula *et al*, 2019; Wu *et al*, 2019; Forrest *et al*, 2020). The codon stabilization coefficient (CSC) describes the correlation between the occurrence of a codon in mRNA transcripts and experimentally determined mRNA stability (Presnyak *et al*, 2015). The CSC for a codon is positive if this codon is overrepresented in stable mRNAs and negative if overrepresented in unstable mRNAs. Similar to Harigaya & Parker (2016), we determined CSC values for *S. pombe* based on large‐scale mRNA half‐life measurements (Hasan *et al*, 2014; Eser *et al*, 2016). The CSC value for each gene (CSC_g_) is the arithmetic mean of the CSC values of all codons in that gene. As had been seen before (Presnyak *et al*, 2015; Harigaya & Parker, 2016), the CSC_g_ correlated with other measures of codon optimality such as the percentage of optimal codons or the tRNA adaptation index (tAI) (Appendix Fig S3A). As the SAC genes had short mRNA half‐lives, we expected them to have low CSC_g_ values. Indeed, *mad2* ^+^ and *mad3* ^+^ were among the 20% of protein‐coding genes with the lowest CSC_g_ values (Fig 3A and B). This result was independent of which large‐scale mRNA half‐life data or which correlation parameter was used (Appendix Fig S3C and D). These results raise the interesting possibility that codon usage in *mad2* ^+^ and *mad3* ^+^ contributes to their short mRNA half‐life. The *mad1* ^+^ gene showed different characteristics, which we will discuss below.

### Codon‐optimization increases the mRNA concentration of *mad2* ^+^ and *mad3* ^+^

To test if codon usage contributes to the short mRNA half‐lives, we codon‐optimized *mad2* ^+^ and *mad3* ^+^ and inserted the codon‐optimized sequence at the respective endogenous locus (Fig 3C; Appendix Fig S3B and F). The GFP tag, which remained unchanged, mitigated but did not abolish the effect of the codon‐optimization on the CSC_g_ value of the fusion genes (Appendix Fig S3B). An increase in mRNA half‐life should result in an increased steady‐state mRNA number if synthesis was unchanged. Indeed, we found an increased mRNA number for codon‐optimized *mad2* and *mad3* compared with the wild‐type gene (Fig 3D). Cytoplasmic mRNAs showed a 27% increase (Fig EV3). For *mad2*, the increase was restricted to the cytoplasm and not observed in the nucleus, strongly suggesting stabilization of the mRNA (Fig EV3).

**Figure EV2:**
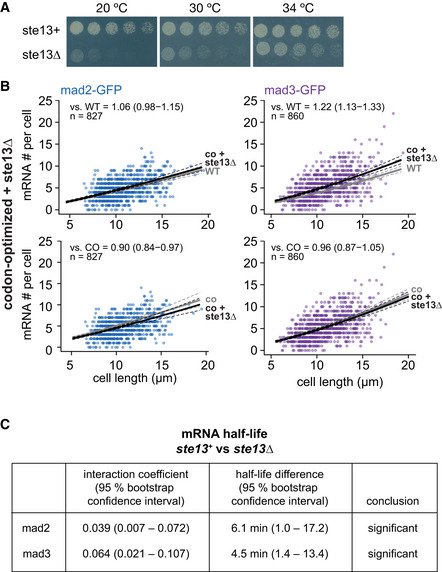
Additional data on *mad2* ^+^ and *mad3* ^+^ mRNA numbers and half‐lives after codon‐optimization or *ste13* ^+^ deletion AGrowth assay for wild‐type and *ste13Δ* cells on minimal medium plates.BScatter plots of whole‐cell mRNA counts versus cell length for cells expressing codon‐optimized mad2‐ or mad3‐GFP and deleted for *ste13* ^+^. Solid lines are regression curves from generalized linear mixed model fits; black for the genotype shown, gray for the respective reference: wild type (WT) in the first row, codon‐optimized (co) in the second row. Dashed lines represent 95% bootstrap confidence bands for the regression curves. Model estimates of the mRNA level relative to the reference with bootstrap 95% confidence intervals in parentheses are included in the plots. Control curves for upper panels from wild‐type *mad2* ^+^ and *mad3* ^+^ data in Fig 3D, and for lower panels from codon‐optimized mad2 and mad3 data in Fig 3D. Two to five replicates per genotype.CStatistical significance for mRNA half‐life changes after deletion of *ste13* ^+^. First and second columns show estimates and 95% bootstrap confidence intervals for the model interaction coefficient and the half‐life difference, respectively. The change in half‐life after deletion of *ste13* ^+^ was considered significant if the 95% bootstrap confidence intervals for the interaction coefficient and the half‐life difference excluded 0. Source data are available online for this figure.

**Figure EV3:**
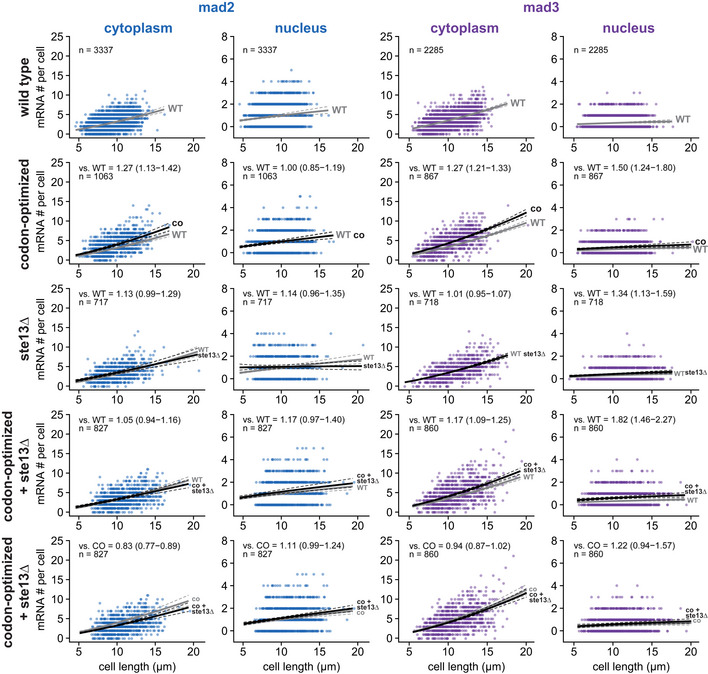
Cytoplasmic and nuclear FISH data for *mad2* ^+^ and *mad3* ^+^ Scatter plots of cytoplasmic and nuclear mRNA counts versus cell length for *mad2* ^+^ and *mad3* ^+^. Solid lines are regression curves from generalized linear mixed model fits; gray is wild type (WT) in rows 1–4 and codon‐optimized (co) in row 5, black is the genotype indicated. Dashed lines represent 95% bootstrap confidence bands for the regression curves. Model estimates for the mRNA ratio between the genotype indicated on the left and the respective reference are included in the plots with bootstrap 95% confidence intervals in parentheses. Same experiments as whole‐cell data in Figs 1D and 3D, and EV2B. Two to five replicates per genotype. Source data are available online for this figure.

In *S. cerevisiae*, the RNA helicase Dhh1 (*S. pombe* Ste13) is involved in specifically lowering the mRNA half‐life of genes with a high fraction of nonoptimal codons (Radhakrishnan *et al*, 2016; Cheng *et al*, 2017; Webster *et al*, 2018; Buschauer *et al*, 2020). Consistently, we observed that the deletion of *ste13* ^+^ significantly increased *mad2* ^+^ and *mad3* ^+^ mRNA half‐lives—from about 8 to 14 min for *mad2* ^+^, and 5 to 10 min for *mad3* ^+^ (Figs 3E and EV2). This indicates that *mad2* ^+^ and *mad3* ^+^ mRNA are subject to Ste13‐mediated degradation. The steady‐state mRNA numbers were not greatly affected by *ste13* ^+^ deletion (Figs 3D and EV2B, and EV3). This is consistent with a global “buffering” of mRNA concentrations that has been observed in budding yeast when mRNA degradation rates or synthesis rates are globally reduced (Haimovich *et al*, 2013; Sun *et al*, 2013; Timmers & Tora, 2018; Fischer *et al*, 2020). Buffering has been found to be a global phenomenon, not observed when the mRNA of single genes is stabilized (Garcia‐Martinez *et al*, 2021). This may explain why mRNA numbers increased after codon‐optimization, but not after *ste13* ^+^ deletion. Overall, our results support the hypothesis that nonoptimal codons in *mad2* ^+^ and *mad3* ^+^ contribute to the short mRNA half‐life of these genes.

### Codon‐optimization, but not *ste13* ^+^ deletion, increases the protein concentration of Mad2 and Mad3

**Figure 4:**
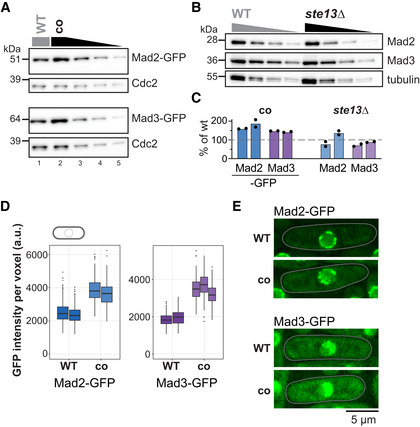
Codon‐optimization increases the protein concentrations of Mad2 and Mad3 AImmunoblot of *S. pombe* protein extracts from cells expressing wild‐type (WT) or codon‐optimized (co) Mad2‐GFP or Mad3‐GFP probed with antibodies against GFP and Cdc2 (loading control). Lanes 3–5 are a 1:1 dilution series of the extract from cells expressing the codon‐optimized version.BImmunoblot of protein extracts from wild‐type (WT) or *ste13Δ* strains probed with antibodies against Mad2, Mad3, and tubulin (loading control). A 1:1 dilution series was loaded for quantification.CEstimates of the protein concentration relative to wild‐type conditions from experiments such as in (A) and (B). Bars are experimental replicates, dots are technical replicates. Two‐sided *t*‐tests: *P* = 0.03 (Mad2‐co), 0.004 (Mad3‐co), 0.82 (Mad2 *ste13Δ*), 0.15 (Mad3 *ste13Δ*).DWhole‐cell GFP concentration from individual live‐cell fluorescence microscopy experiments (a.u., arbitrary units). Boxes show median and interquartile range (IQR); whiskers extend to values no further than 1.5 times the IQR from the first and third quartile, respectively. Codon‐optimized concentration significantly higher than wild type for both genes (generalized linear mixed model). Mad2‐GFP: *n* = 468 and 413; Mad2‐co‐GFP: *n* = 206 and 366; Mad3‐GFP: *n* = 224 and 127; Mad3‐co‐GFP: *n* = 160, 450 and 212 cells.ERepresentative images from one of the experiments in (D). A single Z‐slice is shown. Cells are outlined in gray. Source data are available online for this figure.

To ask whether the consequences of codon‐optimization propagate to the protein level, we quantified Mad2‐ and Mad3‐GFP protein expressed from the wild‐type or codon‐optimized genes. Both immunoblotting (Fig 4A and C) and fluorescence microscopy (Fig 4D and E) showed an increase in protein concentration after codon‐optimization, which can partly be explained by the increase in mRNA (Fig 3) and might be enhanced by an increased translation efficiency. In contrast, the Mad2 and Mad3 protein concentrations in *ste13Δ* cells remained largely stable when analyzed by immunoblotting (Fig 4B and C), consistent with the mRNA results (Fig 3D). Altogether, these data support that codon usage bias toward nonoptimal codons in *mad2* ^+^ and *mad3* ^+^ lowers their protein concentration but supports a short mRNA half‐life, thereby establishing a gene expression pattern that lowers cell‐to‐cell variability.

### *mad1* ^+^ expression regulation differs from that of *mad2* ^+^ and *mad3* ^+^

**Figure 5:**
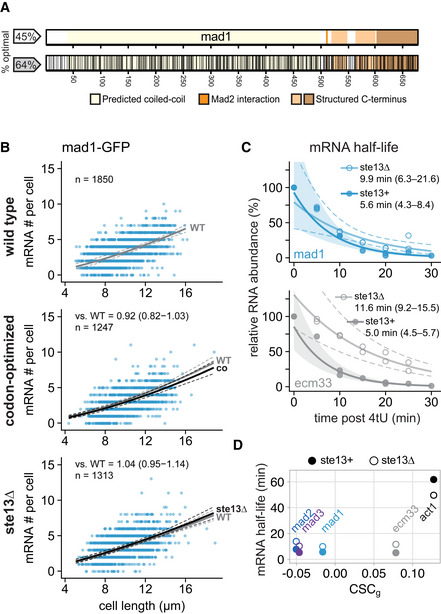
Codon‐optimization and *ste13* ^+^ deletion do not significantly affect the steady‐state mRNA number of *mad1* ^+^ ASchematic of the *mad1* ^+^ gene. Regions coding for important structural features are highlighted. Black lines in the bottom graph indicate synonymous codon changes in the codon‐optimized version.BScatter plots of whole‐cell mRNA counts versus cell length. Solid lines are regression curves from generalized linear mixed model fits (gray: wild type, black: codon‐optimized or *ste13Δ*). Dashed lines: 95% bootstrap confidence bands for the regression curves. Model estimates of the ratio relative to wild‐type mRNA are included with bootstrap 95% confidence interval in brackets. Two to three replicates per genotype.CTime course of RNA abundances by qPCR following metabolic labeling and removal of the labeled pool (two independent experiments). Solid lines: regression curves from generalized linear mixed model fits (dark = *ste13* ^+^, light = *ste13Δ*), excluding *t* = 0 to accommodate for non‐instantaneous labeling by 4tU. Shaded area: 95% bootstrap confidence band for *ste13* ^+^; dashed lines: 95% bootstrap confidence band for *ste13Δ*. Half‐life estimates are included with 95% bootstrap confidence intervals in brackets. See Fig EV4E for additional statistics. The *ste13* ^+^ data are the same as in Fig 2.DComparison between mean CSC values for selected genes (CSC_g_) and mRNA half‐life measured with or without deletion of *ste13* ^+^. mRNA half‐life estimates from Figs 3E and 5C, and EV4D. Source data are available online for this figure.

**Figure EV4:**
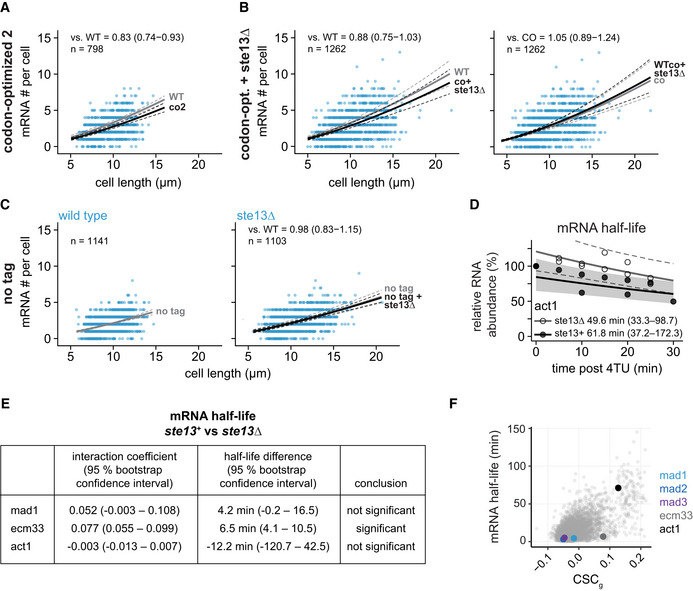
Additional data on *mad1* ^+^ mRNA number and half‐life after codon‐optimization or *ste13* ^+^ deletion AScatter plot of whole‐cell mRNA counts versus cell length. Solid lines are regression curves from generalized linear mixed model (GLMM) fits; black for the genotype shown (co2), gray for the wild‐type (WT) reference. Dashed lines represent 95% bootstrap confidence bands for the regression curves. Model estimates for the mRNA ratio between the genotypes indicated are included in the plots with bootstrap 95% confidence intervals in parentheses. Two to three replicates per genotype.BAs in (A). Black regression line for the genotype shown (co + *ste13Δ*), gray for the respective reference, wild type (WT) or codon‐optimized (co). Two to three replicates per genotype.CScatter plots for whole‐cell mRNA counts of untagged *mad1* ^+^ in *ste13* ^+^ (left) or *ste13Δ* (right) cells, similar to (A). The regression curve for untagged *mad1* ^+^ in *ste13* ^+^ is shown in gray, that for untagged *mad1* ^+^ in *ste13Δ* in black. Probes were against the *mad1* ^+^ coding sequence. One to three replicates per genotype.DTime course of RNA abundances by qPCR following metabolic labeling and removal of the labeled pool (two independent experiments). Solid lines are regression curves from GLMM fits (black = *ste13* ^+^, gray = *ste13Δ*), excluding the measurements at *t* = 0 to accommodate for noninstantaneous labeling by 4tU. Shaded area is 95% bootstrap confidence band for the *ste13* ^+^ curve and dashed lines indicate 95% bootstrap confidence band for the *ste13Δ* curve. Half‐life estimates with 95% bootstrap confidence intervals are included on the plot. The *ste13* ^+^ data are the same as in Fig 2.EStatistical significance for mRNA half‐life changes after deletion of *ste13* ^+^. First and second columns show estimates and 95% bootstrap confidence intervals for the model interaction coefficient and the half‐life difference, respectively. The change in half‐life after deletion of *ste13* ^+^ was considered significant if the 95% bootstrap confidence intervals for the interaction coefficient and the half‐life difference excluded 0.FCSC_g_ values (this study) and mRNA half‐lives (from Eser *et al*, 2016) for protein‐coding *S. pombe* genes with the indicated genes highlighted. Source data are available online for this figure.

**Figure EV5:**
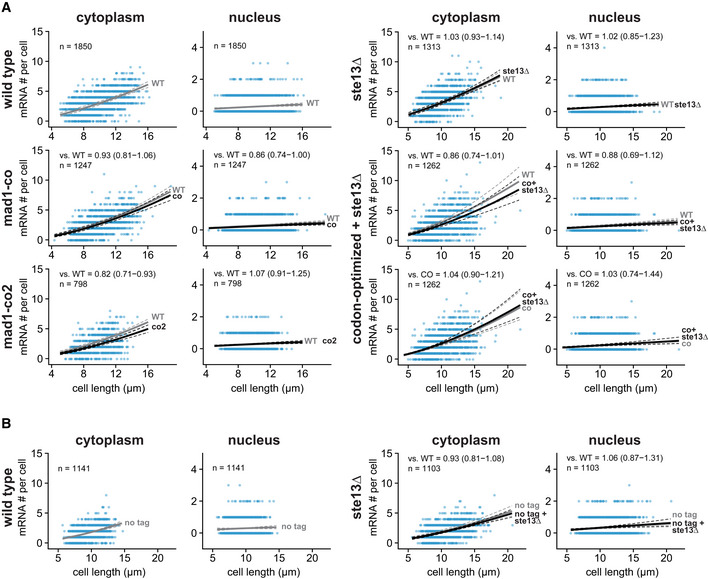
Cytoplasmic and nuclear FISH data for *mad1* ^+^ AScatter plots of cytoplasmic and nuclear mRNA counts versus cell length for *mad1* ^+^. Solid lines are regression curves from generalized linear mixed model fits; gray is wild‐type (WT) *mad1* ^+^‐GFP for all panels, except the bottom row on the right side, where it is codon‐optimized mad1‐GFP (co), black is the genotype indicated on the left. Dashed lines represent 95% bootstrap confidence bands for the regression curves. Model estimates for the mRNA ratio between the genotype indicated on the left and the respective reference are included in the plots with bootstrap 95% confidence intervals in parentheses. Same experiments as whole‐cell data in Figs 1D and 5B, and EV4A and B. Two to three replicates per genotype.BSimilar to (A) but for untagged *mad1* ^+^. Solid lines are regression curves from generalized linear mixed model fits; gray is untagged wild‐type *mad1* ^+^ for all panels, black is untagged *mad1* ^+^ in *ste13Δ*. One to three replicates per genotype. Same experiments as whole‐cell data in Fig EV4B. Source data are available online for this figure.

The *mad1* ^+^ gene shares a short mRNA half‐life with *mad2* ^+^ and *mad3* ^+^ (Fig 2D). Different from *mad2* ^+^ and *mad3* ^+^, though, *mad1* ^+^ has a higher fraction of optimal codons and a CSC_g_ value above the median of all protein‐coding *S. pombe* genes (Fig 3A and B; Appendix Fig S3A and B). This was surprising because we expected similar features within the SAC network. Unlike for *mad2* and *mad3*, the *mad1* mRNA number did not increase after codon‐optimization, but rather decreased slightly (Figs 5A and B, and EV5). A second codon‐optimized *mad1* whose sequence was considerably different from the first (77% nucleotide identity; Appendix Fig S3F and Appendix Table S3) showed the same trend (Figs EV4A and EV5). Similar to *mad2* ^+^ and *mad3* ^+^, *mad1* ^+^ mRNA half‐life was still prolonged in *ste13Δ* cells (from 6 to 10 min; Fig 5C), but unlike for *mad2* ^+^ and *mad3* ^+^ not reaching statistical significance (Fig EV4E). Thus, the short *mad1* ^+^ mRNA half‐life is less dependent on codon usage bias and Ste13, and hence, different modes of regulation bring about the short mRNA half‐life of these SAC genes.

The *ecm33* ^+^ control mRNA was strongly stabilized in *ste13* ^+^‐deleted cells (Figs 5C and EV4E), despite a high fraction of optimal codons in *ecm33* ^+^ (Fig 5D). This highlights that—despite some overall correlation—the relationships between codon optimality, mRNA half‐life, and susceptibility to *ste13* ^+^ deletion are far from predictable (Fig EV4F) (He *et al*, 2018). It is worth noting that Ecm33 is a plasma membrane‐binding protein. The budding yeast and human orthologs of Ste13 (Dhh1 and DDX6, respectively) influence translation and mRNA degradation of membrane‐binding proteins, and budding yeast Dhh1 has been shown to bind Ecm33 and its paralog Pst1 (Jungfleisch *et al*, 2017; Weber *et al*, 2020). If conserved in *S. pombe*, this could explain the strong destabilizing effect of Ste13 on *ecm33* ^+^ mRNA.

### Codon‐optimization of *mad1* ^+^ decreases its protein concentration

**Figure 6:**
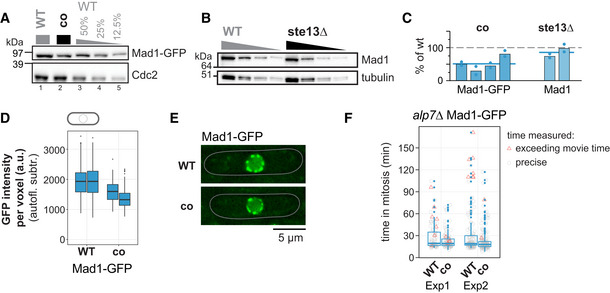
Codon identity in *mad1* ^+^ is important for proper protein concentration AImmunoblot of *S. pombe* protein extracts from cells expressing wild‐type (WT) or codon‐optimized (co) Mad1‐GFP probed with antibodies against GFP and Cdc2 (loading control). Lanes 3–5 are a dilution series of the extract from wild‐type cells.BImmunoblot of protein extracts from wild‐type (WT) or *ste13Δ* strains probed with antibodies against Mad1 and tubulin (loading control). A 1:1 dilution series was loaded for quantification. Tubulin blot is the same as in Fig 4B.CEstimates of the protein concentration relative to wild‐type conditions from experiments such as in (A) and (B). Bars are experimental replicates, dots are technical replicates. Blue lines indicate the mean of all experiments. Two‐sided *t*‐tests: *P* = 0.005 (Mad1‐co, *n* = 4 experimental replicates); *P* = 0.16 (Mad1 *ste13Δ*, *n* = 2).DWhole‐cell GFP concentration from individual live‐cell fluorescence microscopy experiments (a.u. = arbitrary units). Boxplots show median and interquartile range (IQR); whiskers extend to values no further than 1.5 times the IQR from the first and third quartile, respectively. Codon‐optimized concentration significantly lower than wild type (generalized linear mixed model). Mad1‐GFP: *n* = 197 and 224; Mad1‐co‐GFP: *n* = 80 and 377 cells.ERepresentative images from one of the experiments in (D). An average projection of three Z‐slices is shown; cells are outlined in gray.FLive‐cell imaging for time spent in mitosis. The *alp7* ^+^ gene was deleted to increase the likelihood of spindle assembly checkpoint activation. Localization of Plo1‐tdTomato to spindle‐pole bodies was used to judge entry into and exit from mitosis (also see Appendix Fig S4). Exp1: *n* = 73 (WT) and 94 cells (co); Exp2: *n* = 126 (WT) and 152 cells (co). Boxplots show median and interquartile range (IQR); whiskers extend to values no further than 1.5 times the IQR from the first and third quartile, respectively. Measurements for individual cells are shown in addition (gray circles if measurement was exact, red triangles if end of mitosis was not captured because imaging ended). Difference between WT and co: *P* = 0.14 (Exp1) and 0.15 (Exp2) by Kolmogorov–Smirnov test. Source data are available online for this figure.

Unlike Mad2‐ and Mad3‐GFP, whose protein concentration increased after codon‐optimization, that of Mad1‐GFP decreased, both by immunoblotting (Fig 6A and C) and by fluorescence microscopy (Fig 6D and E). Mad1 protein formed from the codon‐optimized mRNA had a similar stability to that formed from wild‐type mRNA (Appendix Fig S4A and B) and still bound Mad2 (Appendix Fig S4C). The reduction, rather than increase, in protein concentration after codon‐optimization of *mad1* ^+^ corroborates that the codon usage pattern of *mad1* ^+^ serves a different purpose than that of *mad2* ^+^ and *mad3* ^+^. Deletion of *ste13* ^+^ had hardly any influence on the Mad1 protein concentration (Fig 6B and C), consistent with the largely unchanged mRNA concentration (Fig 5B).

We previously found that SAC function was well preserved when Mad1 levels were lowered to 30% (Heinrich *et al*, 2013). Consistently, we did not observe an obvious growth defect when cells expressing codon‐optimized *mad1* were grown in the presence of the microtubule drug benomyl (Fig EV1B), and we did not observe a SAC defect in a live‐cell imaging assay where microtubules were depolymerized (Appendix Fig S4D and E). To test SAC function in a more sensitive assay, we deleted the gene for the microtubule‐interacting protein Alp7 (Sato *et al*, 2003). This also activates the SAC, but less robustly than microtubule‐depolymerization. Using this assay, cells expressing codon‐optimized *mad1* tended to exit mitosis more quickly than cells expressing wild‐type *mad1* ^+^ (Fig 6F; Appendix Fig S4F). The difference did not reach the level of statistical significance but was reproducible with independent strains. This suggests that synonymous codon changes, without any change in the protein sequence, can impair SAC function.

### Upstream and downstream sequences of *mad1* ^+^ are insufficient for proper expression

The lower mRNA concentration after *mad1* codon‐optimization (Figs 5B and EV4A) suggested that the concentration of *mad1* ^+^ mRNA is not purely determined by regulatory sequences upstream and downstream of the coding sequence. This is supported by our observation that merely fusing GFP to *mad1* ^+^, without altering surrounding sequences, significantly increases its mRNA number (Figs 1E and EV1E). Further supporting this notion, but rather surprisingly, we found that replacing the *mad1* ^+^ coding sequence with GFP produced neither significant amounts of mRNA nor protein (Appendix Fig S5A and B). This again contrasted with the *mad2* ^+^ and *mad3* ^+^ genes, which produced comparable amounts of mRNA and protein when the original coding sequence was replaced with GFP (Appendix Fig S5C and D). Hence, the sequences surrounding the *mad1* ^+^ coding sequence are insufficient to establish *mad1* ^+^‐like expression, and contributions from the coding sequence are required. Preserving the first 66 or 108 base pairs of *mad1* ^+^ partly rescued both mRNA and protein levels but not completely (Appendix Fig S5A and B). While this suggests that the 5′ region of the *mad1* ^+^ coding sequence carries signals that are important for mRNA synthesis or stabilization, some other genes contain sequences that can compensate. Introducing an *nmt1* ^+^‐GFP fusion gene or fusions between *S. cerevisiae* GCN4 and N‐terminally truncated versions of *S. pombe mad1* ^+^ (Heinrich *et al*, 2014) allowed for expression from the *mad1* ^+^ locus (Appendix Fig S5A and B). What these genes share, that GFP does not, remains unclear.

Altogether, these results indicate that *mad1* ^+^ expression has some unique aspects: *mad1* ^+^ uses a different mode for reducing mRNA half‐life than *mad2* ^+^ or *mad3* ^+^, and its coding sequence carries elements that help transcribe, stabilize, or translate RNA.

### Mad1 homodimers assemble co‐translationally

**Figure 7:**
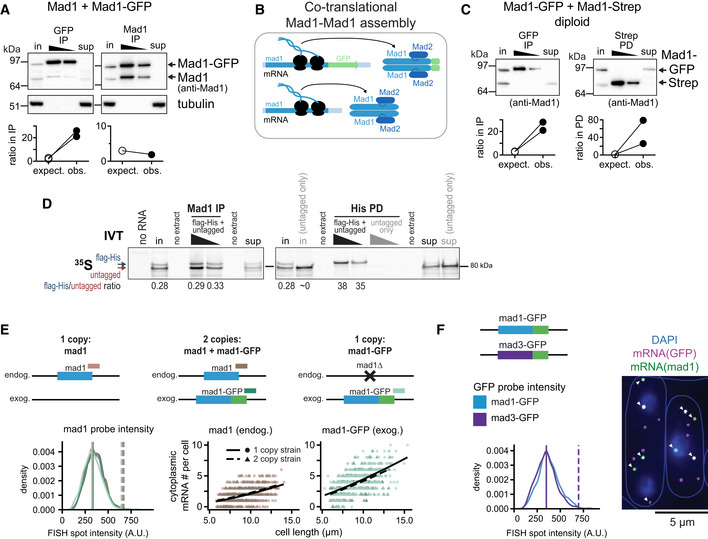
Mad1 homodimers assemble co‐translationally ATop: Immunoprecipitation (IP) with anti‐GFP or anti‐Mad1 from extracts of haploid *S. pombe* cells expressing both untagged and GFP‐tagged Mad1, probed with antibodies against Mad1 and tubulin; in = input (2.5% of extract for IP), sup = supernatant after IP. Bottom: Comparison between the observed (obs.) and the expected (expect.) ratio between Mad1‐GFP and untagged Mad1 in the IP given their ratio in the input (see Fig EV6A); two and one experiment(s), respectively. One more GFP‐IP from the same strain was unquantifiable, because no second band was visible in the IP.BSchematic illustrating that Mad1‐Mad1 complex assembly likely takes place co‐translationally with only proteins synthesized from the same mRNA being combined.CTop: Anti‐GFP immunoprecipitation (IP) and Strep pull‐down (PD) from extracts of diploid cells expressing Mad1‐GFP and Mad1‐Strep from the two endogenous loci; membrane probed with anti‐Mad1; in, input (7% of extract for IP/PD), sup, supernatant after IP/PD. Bottom: as in (A), 2 experiments each. See Fig EV6 for a quantified experiment. The experiment shown at the top and two more GFP‐IPs from the same strain were unquantifiable, because no second band was visible in the IP.D
*In vitro* translation (IVT) of Mad1‐flag‐His and untagged Mad1 in the presence of ^35^S‐labeled Methionine and Cysteine, followed by Mad1 immunoprecipitation (IP) or His pull‐down (PD); in, input (9.5% of extract for IP/PD), sup, supernatant after IP/PD. An IVT with only untagged Mad1 was used to check for specificity of the His PD (right side). Shown is the autoradiograph after SDS‐PAGE with quantification of the Mad1‐flag‐His to untagged Mad1 ratio in select lanes.ETest for mRNA dimerization by single‐molecule mRNA FISH; probes against *mad1* ^+^ and GFP. Top: Schematic of genotypes. Example pictures in Fig EV6. Bottom left: Intensity of cytoplasmic *mad1* ^+^ mRNA spots in the different strains. For the 2 copy strain, a *mad1* ^+^ spot was classified as *mad1* ^+^‐GFP if it was co‐localizing with a GFP spot, and as *mad1* ^+^ otherwise. Colors as indicated in the schematic. Vertical solid line: peak of each density plot; dashed line: theoretical position of a double‐intensity peak. Number of spots analyzed: *mad1* ^+^ (1 copy strain) = 921, *mad1* ^+^ (2 copy strain) = 637, *mad1* ^+^‐GFP (2 copy strain) = 982, *mad1* ^+^‐GFP (1 copy strain) = 1,699. Bottom right: Counts of cytoplasmic *mad1* ^+^ or *mad1* ^+^‐GFP mRNA from the same experiment with generalized linear mixed model fits as lines. Number of cells: 1 copy strain *mad1* ^+^ = 478, 2 copy strain = 327, 1 copy strain *mad1* ^+^‐GFP = 466.FExperiment similar to (E), except that cells expressing both *mad1* ^+^‐GFP and *mad3* ^+^‐GFP from the respective endogenous locus were probed with FISH probes against *mad1* ^+^ and GFP mRNA. A GFP spot was classified as *mad1* ^+^‐GFP if it was co‐localizing with a *mad1* ^+^ spot (arrowheads), and as *mad3* ^+^‐GFP otherwise. The intensity of GFP spots was quantified. Vertical solid line: peak of each density plot; dashed line: theoretical position of a double‐intensity peak. Number of spots analyzed: *mad1* ^+^‐GFP = 987, *mad3* ^+^‐GFP = 1,299. Source data are available online for this figure.

**Figure EV6:**
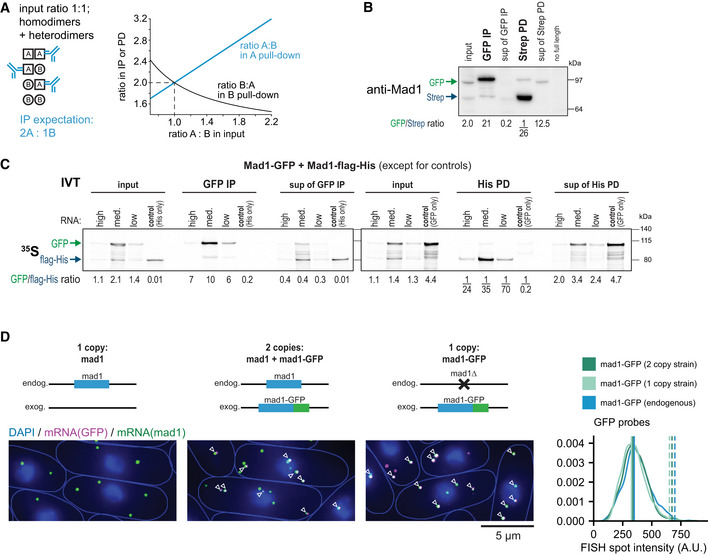
Additional experiments supporting that Mad1 homodimers assemble co‐translationally ATheoretical considerations: if, for two different copies of Mad1, homodimer and heterodimer formation was equally likely, and the ratio in the input was 1:1, one would expect a ratio of 2:1 in the pull‐down. Expectations for other input ratios are shown in the graph. For typical input ratios in our experiments, the maximum expected ratio in IP/PD is around 4:1, whereas we typically observe 10:1 or higher.BReplicate experiment for Fig 7C; one of the experiments quantified at the bottom of Fig 7C. Anti‐GFP immunoprecipitation (IP) and Strep pull‐down (PD) from extracts of diploid cells expressing Mad1‐GFP and Mad1‐Strep from the two endogenous loci; membrane probed with anti‐Mad1; input is 3% of extract used for IP/PD, sup = supernatant. Numbers at the bottom show the quantification of the Mad1‐GFP to Mad1‐Strep ratio. The last lane contains extract of a diploid strain with both copies of endogenous *mad1* ^+^ deleted.C
*In vitro* translation (IVT) of Mad1‐GFP and Mad1‐flag‐His in the presence of ^35^S‐labeled Methionine and Cysteine, followed by GFP immunoprecipitation (IP) or His pull‐down (PD); input is 10% of extract used for IP/PD, sup = supernatant. IVTs with only Mad1‐flag‐His, or only Mad1‐GFP were used to control for the specificity of the IP/PD. High RNA conc. is 40 ng/μl mad1‐GFP and 35 ng/μl mad1‐flag‐His; the medium and low concentrations are 1:10 and 1:100 dilutions of the “high” mix. Shown is the autoradiograph after SDS‐PAGE with quantification of the Mad1‐GFP to Mad1‐flag‐His ratio. One out of two experiments with similar results.DSame experiment as in Fig 7E. Representative images from each strain with co‐localizing *mad1* ^+^ and GFP spots marked by arrowheads. Right side: Intensity of cytoplasmic GFP mRNA spots in the different strains. Vertical solid line: peak of each density plot; dashed line: theoretical position of a double‐intensity peak. Number of spots analyzed: *mad1* ^+^‐GFP (2 copy strain) = 1,178, *mad1* ^+^‐GFP (1 copy strain) = 1,796, *mad1* ^+^‐GFP expressed from the endogenous locus (not shown schematically on the left) = 987. Source data are available online for this figure.

We considered whether *mad1* ^+^ may have a certain codon usage pattern to facilitate protein production or complex formation (Liu *et al*, 2021). Mad1 forms a homodimer through a long N‐terminal coiled‐coil (Sironi *et al*, 2002; Piano *et al*, 2021), but—except in a very recent genome‐wide study (Bertolini *et al*, 2021)—how this homodimer forms has not been examined. If formation was co‐translational rather than post‐translational, this may require a certain pattern of codon usage for proper complex formation. To assess dimer formation, we examined cells expressing both tagged and untagged Mad1. If Mad1 dimer formation was post‐translational, it should be possible to observe interactions between tagged and untagged Mad1. However, in haploid strains expressing a C‐terminally GFP‐tagged and an untagged *mad1* ^+^ gene, a GFP immunoprecipitation almost exclusively precipitated Mad1‐GFP, but not untagged Mad1 (Fig 7A). In contrast, a Mad1 immunoprecipitation precipitated Mad1‐GFP and Mad1 in approximately the same ratio in which they were present in the extract. These experiments used a monomeric version of GFP. Thus, it is unlikely that this pattern is driven by dimerization of GFP. With two versions of Mad1 being expressed, a slight bias toward the form that is being pulled down would be expected even when heterodimers between these forms were generated with equal likelihood as homodimers (Fig EV6A). At a 1:1 ratio of the isoforms in the extract, a 2:1 ratio would be expected in an immunoprecipitation or pull‐down. However, the bias that we observed always exceeded the expected bias, usually vastly (Figs 7 and EV6). Hence, we propose that Mad1 forms homodimers between isoforms more efficiently than heterodimers. This is most easily explained by co‐translational assembly of Mad1 dimers from the nascent chains of two ribosomes translating *mad1* ^+^ from the same mRNA molecule (Fig 7B).

We further corroborated this finding using diploid strains expressing Mad1‐GFP and Mad1‐Strep from the two endogenous loci. Again, a GFP‐immunoprecipitation isolated Mad1‐GFP but very little Mad1‐Strep, whereas a Strep pull‐down isolated Mad1‐Strep but very little Mad1‐GFP (Figs 7C and EV6B). We obtained similar results after *in vitro* translation of Mad1 (Fig EV6C): when Mad1‐GFP and Mad1‐flag‐His were co‐translated in a rabbit reticulocyte lysate, a subsequent GFP immunoprecipitation isolated very little Mad1‐flag‐His, and a His pull‐down isolated very little Mad1‐GFP. Heterodimerization between C‐terminal Mad1 fragments has previously been reported in an *in vitro* translation (Kim *et al*, 2012). However, in our experiments, even C‐terminal fragments showed a strong bias toward the form that was being precipitated, both in yeast extracts and after *in vitro* translation (Appendix Fig S6). To exclude that heterodimer formation between Mad1‐GFP and untagged Mad1 was nonphysiologically prevented by the large GFP tag, we tested a combination of Mad1‐flag‐His and untagged Mad1 in an *in vitro* translation. Again, His pull‐down almost exclusively isolated Mad1‐flag‐His, whereas a Mad1 immunoprecipitation isolated both forms in approximately the same ratio in which they were present in the extract (Fig 7D).

To further test the idea that Mad1 dimer assembly occurs on a single mRNA molecule (Fig 7B), we examined *mad1* ^+^ mRNA. Consistent with few heterodimers on the protein level, we did not observe co‐localization between two different *mad1* ^+^ isoform mRNAs present in the same cell (Fig 7E). Intensity measurements of mRNA FISH spots suggested the presence of single mRNAs, not mRNA doublets, when both untagged *mad1* ^+^ and *mad1* ^+^‐GFP were expressed and mRNA spots were detected with a *mad1* ^+^ probe (Fig 7E, left; EV6D). Further supporting this finding, the number of *mad1* ^+^ mRNA spots that were co‐localizing with GFP spots (indicating *mad1* ^+^‐GFP) or not (indicating untagged *mad1* ^+^) was identical in strains expressing one or both isoforms (Fig 7E, right), indicating that the isoforms do not co‐localize. We additionally tested the possibility that mRNAs of the same isoform may co‐localize by comparing FISH spot intensities with probes against GFP between *mad1* ^+^‐GFP mRNA and *mad3* ^+^‐GFP mRNA (the latter coding for Mad3 monomers). We did not find any difference in spot intensity (Fig 7F). Hence, we conclude that *mad1* ^+^ mRNAs rarely, if ever, co‐localize, and we favor the idea that Mad1 homodimers emerge from two ribosomes co‐translating a single mRNA (Fig 7B).

The fact that Mad1 homodimers form co‐translationally is consistent with the idea that synonymous codon changes may subtly impair complex formation and therefore translation efficiency and mRNA stability. Overall, these results suggest that codon usage bias within *mad1* ^+^ contributes to maintaining proper mRNA and protein levels, possibly by supporting Mad1 folding and dimerization.

## Discussion

Proteins are the workhorses of cells. The deployment of this workhorse army is controlled by regulatory elements encoded in DNA that are still incompletely understood. The spindle assembly checkpoint is sensitive to expression changes, and we therefore asked which features of gene expression may be important for its proper function. Our results suggest that a combination of short mRNA half‐lives and long protein half‐lives is important to keep protein variability low. We also find that—despite their closely shared function—*mad1* ^+^ differs in its expression features from *mad2* ^+^ and *mad3* ^+^. The coding sequences of *mad2* ^+^ and *mad3* ^+^ contribute to the short mRNA half‐life of these genes, whereas that of *mad1* ^+^ contributes to maintaining mRNA (Appendix Fig S5) and protein levels (Fig 6). We propose that the choice of synonymous codons in *mad1* ^+^ is optimized for the formation of the Mad1 homodimer, and ultimately the Mad1/Mad2 complex.

### Short mRNA half‐life of constitutively expressed SAC genes favors low noise

The short mRNA half‐lives of *mad1* ^+^, *mad2* ^+^, and *mad3* ^+^, along with their long protein half‐lives, can explain the low protein noise of SAC genes despite low and variable mRNA numbers (Figs 1 and 2) (Thattai & van Oudenaarden, 2001). In human cells, a long protein half‐life has also been shown to buffer the effects of variable mRNA numbers (Raj *et al*, 2006). Human Mad1, Mad2, and BubR1 (Mad3 ortholog) are also highly stable proteins (Suijkerbuijk *et al*, 2010; Varetti *et al*, 2011; Schweizer *et al*, 2013; Rodriguez‐Bravo *et al*, 2014), which will support stable protein concentrations over time and between cells. SAC genes are certainly not unique in combining a short mRNA and long protein half‐life to achieve low noise. Other constitutively expressed genes that produce low or modest amounts of protein will likely show a similar behavior. Keeping noise low in this manner requires a high turnover of mRNA that confers some energy cost. An alternative way to keep protein noise low would be to produce the same amount of protein from a larger number of more stable mRNA molecules (Appendix Fig S2C). Several side‐effects likely prohibit this solution as a general strategy. For example, the cytoplasm would be much more crowded with mRNAs, and stable mRNAs may accumulate chemical damage. Indeed, genes using an expression strategy of high transcription and low translation rates are exceedingly rare among different eukaryotes (Hausser *et al*, 2019).

### Different SAC genes employ different strategies for achieving short mRNA half‐life

The half‐life of an mRNA is influenced by sequence motifs, codon usage, and other factors that influence translation. Currently, known factors predict around 50–60% of mRNA half‐life in budding yeast (Neymotin *et al*, 2016; Cheng *et al*, 2017). At least two elements seem to play a role for *mad2* ^+^ and *mad3* ^+^: Our data suggest that the mRNA half‐lives are shortened by a high fraction of nonoptimal codons (Fig 3); in addition, the *mad2* ^+^ and *mad3* ^+^ 3′ UTRs contain sequence motifs that are associated with a short mRNA half‐life (Eser *et al*, 2016). We previously found higher mRNA numbers after traditional tagging, which changed the 3′ UTR to that of a highly expressed gene (Heinrich *et al*, 2013), suggesting that the predicted motifs in the 3′ UTR may indeed be functional. For *mad1* ^+^, in contrast, overall codon usage bias seems to play a lesser role (Fig 5), and the *mad1* ^+^ 3′ UTR does not contain reported motifs implicated in half‐life shortening (Eser *et al*, 2016). We suspect that other elements that influence translation efficiency may be important. Generally, less efficiently translated mRNAs are less stable (Hanson & Coller, 2018), and *mad1* ^+^ seems to be translated less efficiently than *mad2* ^+^ or *mad3* ^+^ (Rubio *et al*, 2020).

### Formation of the Mad1/Mad2 complex involves co‐translation assembly of the Mad1 dimer but not synchronous co‐translational assembly of the tetramer

Mad1 and Mad2 form a tight tetrameric complex (Sironi *et al*, 2002; Kim *et al*, 2012), but how this complex assembles is unknown. Our experiments suggest that the Mad1 homodimer forms between two polypeptides translated from the same mRNA, and that Mad1 molecules translated from different mRNA molecules associate very inefficiently with each other, if at all (Fig 7). This assembly mode is further supported by a recent study in human cells, which analyzed footprints of ribosome disomes on mRNA and found wide‐spread evidence for co‐translational assembly of protein homomers (Bertolini *et al*, 2021). Coiled‐coils were the most prominent domain class driving co‐translational assembly, and co‐translational assembly was more likely when the dimerization domain was N‐terminal. Mad1 meets both these criteria and was indeed identified in this study as probably assembling co‐translationally.

At least two studies have expressed Mad1 N‐terminal fragments and full‐length Mad1 from two different loci and have interpreted the failure to see association between those two as an inability of the N‐terminal fragment to dimerize (Jin *et al*, 1998; Ji *et al*, 2018). Based on the evidence for co‐translational homodimer assembly, we suggest that the capacity of an N‐terminal Mad1 fragment to dimerize would need to be based on assessing self‐association rather than assessing association with Mad1 expressed from a different locus. Of note, C‐terminal Mad1 fragments also dimerize, possibly post‐translationally (Kim *et al*, 2012), although our own experiments still suggest a preference of homodimerization (Appendix Fig S6).

While we propose that assembly of the Mad1 homodimer occurs co‐translationally, the assembly of the Mad1/Mad2 tetramer does not occur in synchronous co‐translational fashion, as the mRNAs for *mad1* ^+^ and *mad2* ^+^ do not co‐localize in the cytoplasm (Fig 1). This leaves open the possibility of post‐translational assembly of the tetramer or of asynchronous co‐translational assembly, where one protein is already fully formed and binds the other that is being translated (Duncan & Mata, 2011; Shiber *et al*, 2018). Formation of the C‐Mad2/Cdc20 complex necessitates catalysis (Kulukian *et al*, 2009; Lad *et al*, 2009; Simonetta *et al*, 2009; Faesen *et al*, 2017; Piano *et al*, 2021), making it likely that C‐Mad2/Mad1 formation also needs to be facilitated. We favor the idea that the tetramer assembles while one of the proteins is being translated, and it will be interesting to test whether the *mad1* ^+^ mRNA binds Mad2 protein or vice versa to facilitate such an assembly. It will also be interesting to examine whether different eukaryotes use the same assembly pathway for the highly conserved Mad1/Mad2 complex.

### Potential SAC malfunction from synonymous mutations

Overall, our data suggest that the coding sequences of *mad1* ^+^, *mad2* ^+^, and *mad3* ^+^ modulate gene expression. Hence, even synonymous mutations carry some risk of impairing the SAC. We suspect that *mad1* ^+^ is most susceptible to single synonymous substitutions, given the need for co‐translational homodimer assembly (Fig 7), which may be facilitated by controlling the speed of ribosome movement (Liu *et al*, 2021). In *S. pombe*, a cluster of nonoptimal codons follows the coiled‐coil region of *mad1* ^+^ (Appendix Figs S3E and S7), which may ensure that the N‐terminal coiled‐coil is fully formed before the remainder of Mad1 is translated.

It will be interesting to test whether synonymous mutations found in cancer samples can modulate SAC gene expression or function. Within MAD2L1 (H.s. *mad2* ^+^), synonymous mutations detected in cancer samples seem to cluster in a conserved region with high CSC values preceding the “seat belt,” (Appendix Fig S7) suggesting that codon usage bias in this region may be functionally important. Although most synonymous mutations will only have small effects, they may fuel carcinogenesis. This is particularly true in the context of the SAC, because drastic impairment is more likely to be detrimental for cancer cells, whereas subtle impairment may promote carcinogenesis (Kops *et al*, 2004; Funk *et al*, 2016; Cohen‐Sharir *et al*, 2021; Quinton *et al*, 2021). Synonymous mutations and changes in tRNA expression have been implicated in carcinogenesis (Sauna & Kimchi‐Sarfaty, 2011; Supek *et al*, 2014). Our data suggest that this may partly occur by impairing the SAC.

## Materials and Methods

### Reagents and Tools table

**Table:** 

Reagent/Resource	Reference or Source	Identifier or Catalog Number
**Experimental models**		
*Schizosaccharomyces pombe* strains	This study	Appendix Table S1
*Saccharomyces cerevisiae* strain	Nick Buchler, NC State University, USA	Appendix Table S1
**Recombinant DNA**		
sgRNA sequences	This study	Appendix Table S2
Codon‐optimized *mad1*, *mad2*, and *mad3*	This study	Appendix Table S3
PCR fragments for *in vitro* transcription	This study	Appendix Table S6
**Antibodies**		
Mouse anti‐Cdc13 (monoclonal)	Novus	Cat # NB200‐576; RRID: AB_10003103
Rabbit anti‐Cdc2 (polyclonal)	Santa‐Cruz	Cat # sc‐53; RRID: AB_2074908
Mouse anti‐GFP (mix of 2 monoclonals)	Roche	Cat # 11814460001; RRID: AB_390913
Rabbit anti‐Mad1 (polyclonal, against peptide ADSPRDPFQSRSQLC)	Heinrich *et al* (2013), PMID: 24161933	N/A
Rabbit anti‐Mad2 (polyclonal, against recombinant protein)	Sewart and Hauf (2017), PMID: 28366743	N/A
Rabbit anti‐Mad3 (polyclonal, against recombinant protein)	Sewart and Hauf (2017), PMID: 28366743	N/A
Rabbit anti‐Strep‐tag II (monoclonal, recombinant)	Abcam	Cat # ab180957
Rabbit anti‐Strep‐tag II (polyclonal)	Abcam	Cat # ab76949; RRID: AB_1524455
Mouse anti‐tubulin	Sigma	Cat # T5168; RRID: AB_477579
Goat anti‐mouse HRP	Jackson ImmunoResearch Labs	Cat # 115–035‐003; RRID: AB_10015289
Goat anti‐rabbit HRP	Jackson ImmunoResearch Labs	Cat # 111–035‐003; RRID: AB_2313567
**Oligonucleotides and other sequence‐based reagents**		
FISH probes	This study	Appendix Table S4
qPCR primers	This study	Appendix Table S5
**Chemicals, enzymes, and other reagents**		
4‐thiouracil (4tU)	Chem Impex	Cat # 21484
MTSEA biotin‐XX	Biotium	Cat # 90066
Cycloheximide (from Streptomyces griseus)	Chem Impex	Cat # 00083
Wizard SV Gel and PCR Clean‐Up System	Promega	Cat # A9285
SuperScript IV First Strand Synthesis System	ThermoFisher	Cat # 18091050
HiScribe T7 ARCA mRNA Kit (with tailing)	New England Biolabs	Cat # E2060S
Monarch RNA Cleanup Kit	New England Biolabs	Cat # T2040S
Rabbit Reticulocyte Lysate, Nuclease‐Treated	Promega	Cat # L4960
EasyTag EXPRESS 35S Protein Labeling Mix	Perkin Elmer	Cat # NEG772007MC
SUPERase•In RNase Inhibitor	ThermoFisher	Cat # ACM2694
SuperSignal West Pico PLUS Chemiluminescent Substrate	ThermoFisher	Cat # 34580
cOmplete, EDTA‐free Protease Inhibitor Cocktail	Roche	Cat # 04693132001
Halt Protease Inhibitor Cocktail, EDTA‐Free (100X)	ThermoFisher	Cat # 87785
PhosSTOP	Roche	Cat # 04906837001
Halt Phosphatase Inhibitor Cocktail	ThermoFisher	Cat # 78420
Dynabeads Protein G	ThermoFisher	Cat # 10003D
Dynabeads His‐Tag Isolation and Pull‐down	ThermoFisher	Cat # 10103D
MagStrep “type3” XT beads	IBA Lifesciences	Cat # 2–4090‐002
Dynabeads MyOne Streptavidin C1	Thermo Fisher	Cat # 65001
Oligo d(T)_25_ Magnetic Beads	New England Biolabs	Cat # S1419S
Pierce BCA Protein Assay Kit	ThermoFisher	Cat # 23225
EMM (Edinburgh's Minimal Medium)	MP Biomedicals	Cat # 114110022
Lectin	Sigma	Cat # L1395
**Software**		
Fiji/ImageJ	Schindelin *et al* (2012), PMID: 22743772	https://imagej.net/software/fiji/; RRID: SCR_002285
SoftWoRx	Applied Precision, GE Healthcare	https://download.cytivalifesciences.com/cellanalysis/download_data/softWoRx/6.5.2/SoftWoRx.htm; RRID: SCR_019157
MetaMorph	Molecular Devices	Version 7.10.1
YeaZ	Dietler *et al* (2020), PMID: 33184262	N/A
ImageLab	Bio‐Rad Laboratories	Version 6.0.1 build 34
Matlab	Mathworks	https://www.mathworks.com ; RRID: SCR_001622
FISH‐Quant	Mueller *et al* (2013), PMID: 23538861	N/A
Trainable Weka Segmentation	Arganda‐Carreras *et al* (2017), PMID: 28369169	N/A
Prism 9	GraphPad Software, Inc	https://www.graphpad.com ; RRID: SCR_002798
R	Cran.R	https://cran.r‐project.org ; RRID: SCR_001905
R studio	N/A	https://www.rstudio.com ; RRID: SCR_000432
tidyverse package	Cran.R	https://tidyverse.tidyverse.org ; RRID: SCR_019186, Version 1.3.1
ggplot2 package	Cran.R	https://ggplot2.tidyverse.org/ ; RRID: SCR_014601
alphashape3d package	Cran.R	https://CRAN.R‐project.org/package=alphashape3d, Version 1.3.1
boxcoxmix package	Cran.R	https://cran.r‐project.org/src/contrib/Archive/boxcoxmix/, Version 0.28
broom package	Cran.R	https://CRAN.R‐project.org/package=broom, Version 0.7.9
broom.mixed package	Cran.R	https://CRAN.R‐project.org/package=broom.mixed, Version 0.2.7
cairo package	Cran.R	https://CRAN.R‐project.org/package=Cairo, Version 1.5–12.2
cowplot package	Cran.R	https://cran.r‐project.org/package=cowplot ; RRID: SCR_018081, Version 1.1.1
descTools package	Cran.R	https://cran.r‐project.org/package=DescTools, Version 0.99.43
egg package	Cran.R	https://CRAN.R‐project.org/package=egg, Version 0.4.5
geometry package	Cran.R	https://CRAN.R‐project.org/package=geometry, Version 0.4.5
gridExtra package	Cran.R	https://CRAN.R‐project.org/package=gridExtra, Version 2.3
lemon package	Cran.R	https://CRAN.R‐project.org/package=lemon, Version 0.4.5
lme4 package	Cran.R	https://cran.r‐project.org/web/packages/lme4/index.html; RRID: SCR_015654
Irescale package	Cran.R	https://CRAN.R‐project.org/package=Irescale, Version 2.3.0
MASS package	Cran.R	https://cran.r‐project.org/package=MASS ; RRID: SCR_019125
mclust package	Cran.R	https://cran.r‐project.org/package=mclust
nabor package	Cran.R	https://cran.r‐project.org/package=nabor
pbkrtest package	Cran.R	https://cran.r‐project.org/package=pbkrtest
plotly package	Cran.R	https://plotly.com/r/; RRID: SCR_013991, Version 4.10.0
plyr package	Cran.R	https://cran.r‐project.org/package=plyr
readxl package	Cran.R	https://cran.r‐project.org/web/packages/readxl/index.html ; RRID: SCR_018083, Version 1.3.1
rgl package	Cran.R	https://CRAN.R‐project.org/package=rgl, Version 0.107.14
sf package	Cran.R	https://CRAN.R‐project.org/package=sf
shotGroups package	Cran.R	https://CRAN.R‐project.org/package=shotGroups, Version 0.8.1
spatstat package	Cran.R	https://cran.r‐project.org/package=spatstat
**Other**		
Mixer mill MM400	Retsch	Cat # 20.745.0001
Grinding jar 10 ml	Retsch	Cat # 01.462.0236
Grinding jar 25 ml	Retsch	Cat # 01.462.0213
Adapter for reaction vials	Retsch	Cat # 22.008.0008
Glass beads, acid‐washed	Sigma	Cat # G8772
μ‐Slide 8‐well, glass bottom	Ibidi	Cat # 80827
Y04C Microfluidic Plate for Haploid Yeast	CellAsic / Sigma	Cat # Y04C‐02‐5PK
Invitrogen NuPAGE 4 to 12%, Bis‐Tris, 20‐well	Invitrogen	Cat # WG1402BOX
Invitrogen NuPAGE 4 to 12%, Bis‐Tris, 20‐well	Invitrogen	Cat # NP0322BOX
Immobilon‐P PVDF membrane	Millipore	Cat # IPVH00010

### Methods and Protocols

#### Yeast strains

Yeast strains are listed in Appendix Table S1. Tagging of *nmt1* ^+^ and deletion of *ste13* ^+^ and *alp7* ^+^ were performed by conventional PCR‐based gene targeting (Bähler *et al*, 1998). Marker‐less insertion at the endogenous locus was performed either by replacement of a counter‐selectable rpl42‐hphNT1 cassette in an rpl42::cyhR(sP56Q) background (Roguev *et al*, 2007) or by using CRISPR/Cas9 (Jacobs *et al*, 2014). Sequences used for targeting Cas9 are listed in Appendix Table S2. The *mad2* ^+^ *‐ymEGFP* strain contains a single, silent (AGG to AGA) PAM site mutation at amino acid position 173 of Mad2. The *mad3* ^+^ *‐ymEGFP* strain contains a single, silent (TTG to TTA) PAM site mutation at amino acid position 199 of Mad3. Yeast, monomeric‐enhanced GFP (ymEGFP) was derived from yEGFP (yeast codon‐optimized green fluorescent protein (Watson *et al*, 2008)) by mutation of Alanine 206 to Arginine (A206R), which is expected to reduce dimerization (Zacharias *et al*, 2002). Codon‐optimization used proprietary algorithms by two different companies, and sequences are listed in Appendix Table S3. The haploid strain with two differently tagged versions of *mad1* ^+^ has *mad1* ^+^ *‐ymEGFP* along with 110 bp upstream and 164 bp downstream of the coding sequence integrated between the *leu1* ^+^ and *apc10* ^+^ gene.

#### Yeast cultures

*Schizosaccharomyces pombe* cultures were grown at 30°C either in rich medium (yeast extract supplemented with 0.15 g/l adenine; YEA) or in Edinburgh minimal medium (EMM, MP Biomedicals, 4110012) supplemented with 0.2 g/l leucine, 0.15 g/l adenine or 0.05 g/l uracil if required (Petersen & Russell, 2016). When cultures in minimal medium were started at low concentration, “pre‐conditioned medium” was added to a maximum of 50%. Preconditioned medium was obtained by growing cells in EMM and then removing the cells by filtration. For growth assays, cells were grown in YEA to a concentration of around 1 × 10^7^ cells/ml, diluted to 4 × 10^5^ cells/ml in YEA and further diluted in a 1:5 dilution series. 10 μl were spotted on indicated plates. *S. cerevisiae* cultures were grown at 30°C in yeast extract supplemented with 20 mg/ml each of Bacto peptone and dextrose (YPD).

#### Cycloheximide treatment for determination of protein half‐lives

Cells were grown in EMM (plus supplements required for auxotrophic mutations) to a final concentration of around 1 × 10^7^ cells/ml. Cultures were diluted to 8 × 10^6^ cells/ml, transferred to a 30°C water bath for 30 min and a sample was taken prior to the addition of cycloheximide (CHX) to a final concentration of 1 mg/ml. Cells were collected at specified time points, spun down at 980 rcf, and frozen in liquid nitrogen before processing.

#### *In vitro* transcription and translation

The T7 promoter was appended 5′ of the *mad1* ^+^ transcription start site by PCR. Precise sequences are available in Appendix Table S6. Full‐length *mad1* ^+^ was amplified from cDNA generated using the SuperScript IV First Strand Synthesis System (ThermoFisher). *Mad1* fragments 3′ of the intron were amplified from genomic DNA. PCR fragments were purified using the Wizard SV Gel and PCR Clean‐Up System (Promega). *In vitro* transcription was carried out with the HiScribe T7 ARCA mRNA Kit (with tailing) (New England Biolabs) using between 25 and 70 ng/μl template DNA. Reactions were run at 32°C or 37°C for 2 h. RNA was purified using the Monarch RNA Cleanup Kit (New England Biolabs). RNAs were mixed and diluted as required before adding them to rabbit reticulocyte lysate (Promega). Translation reactions contained amino acid mix without Methionine, approx. 1 mCi/ml ^35^S‐Methionine/Cysteine mix (Perkin Elmer, NEG772007MC), 0.2 U/μl SUPERase•In RNase Inhibitor (ThermoFisher), and between 0.35 and 40 ng/μl RNA. Incubation was at 30°C for 1 h 30 min.

#### Denatured whole‐cell extracts

Cells were grown to a final concentration of around 1 × 10^7^ cells/ml and collected by centrifugation (1 × 10^8^ cells per sample). Supernatant was removed, and cells were washed with 1 ml of 20% trichloroacetic acid (TCA). Supernatant was removed, and cells were resuspended in 500 μl of water. 75 μl of NaOH/beta‐mercaptoethanol (final conc. = 0.22 M NaOH, 0.12 M b‐ME) was added, and samples incubated on ice for 15 min. 75 μl of 55% TCA was added and samples incubated on ice for another 10 min. Samples were spun at 16,900 rcf for 10 min at 4°C, and supernatant was removed. Pellets were resuspended in 100 μl sample buffer (50 μl of 2x HU buffer [8 M urea, 5% SDS (w/v), 200 mM Tris–HCl pH 6.8 (v/v), 20% glycerol (v/v), 1 mM EDTA (v/v), 0.1% (w/v) bromophenol blue], 40 μl water, and 10 μl of 1 M DTT) to a final concentration corresponding to 1 × 10^9^ cells/ml. Approximately 150 μl of acid‐washed beads (Sigma) were added before agitation in a ball mill (Mixer Mill 400; Retsch) for 2 min at 30 Hz. Tubes were pierced at the bottom, cell extract was collected from the beads by centrifugation at 2,350 rcf for 1 min and heated at 75°C for 5 min. Typically, the extract equivalent of 2–3 × 10^6^ cells was loaded for immunoblotting.

#### Immunoprecipitation or pull‐down from yeast cell extract

Asynchronously growing cultures were harvested, washed with deionized water, or with 20 mM Tris pH 7.5/150 mM NaCl, and frozen as droplets in liquid nitrogen. Cell powder was prepared from these droplets using a ball mill (Mixer Mill 400; Retsch) for 30 s at 30 Hz under cryogenic conditions. Cell powder was resuspended in lysis buffer (20 mM Tris pH 7.5, 150 mM NaCl, 5% glycerol, and 0.1% NP‐40), and protein concentration was determined by BCA assay (ThermoFisher). For immunoprecipitations, powder was resuspended to a final concentration of 15–20 mg/ml in lysis buffer supplemented with a 5–10x final concentration of protease inhibitor cocktail and a 1x final concentration of phosphatase inhibitor cocktail. Extracts were spun down for 10 min at 4°C and 16,900 rcf. For the input sample, supernatant was mixed with an equal volume of sample buffer (2x HU buffer with 200 mM DTT, or 2x NuPAGE LDS sample buffer with 10% beta‐mercaptoethanol) and heated for 3–5 min at 75°C. For immunoprecipitations, Protein G Dynabeads (ThermoFisher) were covalently coupled with anti‐GFP antibodies (Roche, 160 μg antibody per 1 ml bead suspension) or anti‐Mad1 antibodies (80 μg antibody per 1 ml bead suspension). Strep‐tag pull‐downs used MagStrep “type3” XT beads (IBA Lifesciences). Immunoprecipitations used around 30 μl bead suspension per 200 μl of extract and were performed for 10 min at 4°C on a rotating wheel. Strep pull‐downs used around 200 μl bead suspension per 200 μl of extract and were performed for 45 min to 1 h at 4°C on a rotating wheel. Beads were washed with lysis buffer (IPs), or with a more stringent wash buffer (20 mM Tris pH 7.5, 300 mM NaCl, 5% glycerol, 1% NP‐40) for some Strep pull‐downs. Elution from anti‐GFP or anti‐Mad1 beads was performed by the addition of 7–25 μl 100 mM citric acid and gentle agitation for 5 min at 4°C. Samples were neutralized by the addition of 1.5 M Tris pH 9.2, mixed with an equal volume of sample buffer and heated at 75°C for 3 min. Elution from MagStrep beads was performed with sample buffer and incubation at 95°C for 2 min, or 85°C for 5 min.

#### Immunoprecipitation or pull‐down after *in vitro* translation

*In vitro* translation reactions (IVTs) were diluted to 6‐ to 13‐times the original volume with either Tris buffer for immunoprecipitations (final concentration: 20 mM Tris pH 7.5, 150 mM NaCl, 0.1% NP‐40), or with sodium‐phosphate buffer for Ni‐NTA pull‐downs (final concentration: 50 mM sodium‐phosphate pH 8.0, 300 mM NaCl, 0.01% Tween‐20). Immunoprecipitations used 10 μl Dynabeads suspension, Ni‐NTA pull‐downs used 40 μl Ni‐NTA Dynabeads suspension per 15 μl original IVT (volume prior to dilution). Immunoprecipitations were processed as above, Ni‐NTA beads were washed with sodium‐phosphate buffer plus 10–20 mM imidazole and 0.1% NP‐40 and eluted with sodium‐phosphate buffer plus an additional 300 mM imidazole.

#### Immunoblotting

Proteins were separated by SDS‐PAGE (NuPAGE, Bis‐Tris, MOPS buffer, Thermo Fisher) and transferred onto a PVDF membrane (Immobilon‐P, Millipore) in a semidry blotting assembly (Amersham Biosciences TE‐70 ECL) using transfer buffer (39 mM glycine, 48 mM Tris base) with 10% methanol, 0.01% SDS, and 1:1,000 NuPAGE Antioxidant. Membranes were probed with mouse anti‐GFP (Roche, 11814460001), rabbit anti‐Cdc2 (CDK1, Santa Cruz, SC‐53), mouse anti‐Cdc13 (cyclin B, Novus, NB200‐576), rabbit anti‐Mad1 (Heinrich *et al*, 2013), rabbit anti‐Mad2 (Heinrich *et al*, 2013), rabbit anti‐Mad3 (Heinrich *et al*, 2013), rabbit anti‐Strep (Abcam, ab180957 and ab76949), or mouse anti‐tubulin (Sigma, T5168). Secondary antibodies were either anti‐mouse or anti‐rabbit conjugated to HRP (Dianova) and quantified by chemiluminescence using SuperSignal West Dura ECL (ThermoFisher) and imaged on a Bio‐Rad Gel Doc system. Chemiluminescence signals were quantified on nonsaturated images using Image Lab software (Bio‐Rad). Measurements from a reference dilution series were used to create a standard curve, which was used to determine the concentration of sample relative to the reference. Membranes with radioactive proteins were dried and exposed to a phosphorscreen (GE Healthcare), which was read‐out on a Typhoon phosphorimager (GE Healthcare/Cytiva).

#### Quantification of GFP fusion proteins in single cells (3D segmentation)

To quantify GFP fusion proteins in single cells, cells were grown in EMM (plus supplements that were required for auxotrophic mutations) at 30°C to a final concentration of 6–9 × 10^6^ cells/ml. Cultures of GFP‐positive and GFP‐negative cells were mixed at a 1:1 ratio to a final concentration of 2.5–6.0 × 10^6^ cells/ml and incubated for 30 min at 30°C. To ensure a uniform and flat imaging plane, cells were loaded into a Y04C microfluidics trapping plate (Millipore Sigma) and incubated inside a climate‐controlled microscope chamber for 2 h at 30°C with constant flow of fresh media. Imaging was performed on a DeltaVision Elite system equipped with a PCO edge sCMOS camera and an Olympus 60x/1.42 Plan APO oil objective. Images were acquired for ymEGFP, tdTomato, and brightfield as 7.2 μm or 10 μm stacks with images separated by 0.1 μm. The acquired image area was 1,024 × 1,024 pixels with 1 × 1 binning. All images were deconvolved using SoftWoRx software. To correct for uneven illumination, deconvolved fluorescence images were flatfielded individually for each channel using a custom FIJI script (Baybay *et al*, 2020).

The Pomegranate image analysis pipeline (Baybay *et al*, 2020) was used to segment nuclei (using TetR‐tdTomato‐NLS) and whole cells (using brightfield signal and spherical extrusion of the midplane segmentation) (Appendix Fig S1A). We corrected for chromatic aberration and for stretching of distances in the Z direction (Baybay *et al*, 2020). Further analysis was conducted in R (R Core Team, 2020), and figures were produced using the package ggplot2 (Wickham, 2016).

Only information from mono‐nucleated cells for which both the whole cell and the nucleus had been segmented was retained. Cells were excluded if one or more of the following conditions were met: the nuclear segmentation protruded beyond the three‐dimensional bounds of the cell; whole‐cell segmentation was cut‐off by more than two slices because insufficient slices in Z had been recorded; cell was at the image edge and incompletely recorded; the nucleus had an aspect ratio (diameter in Z to diameter in XY) of less than 0.8 or more than 1.2; cell volume was lower than the 0.1^st^ or higher than the 99.9^th^ percentile. Cells with or without GFP signal were distinguished by k‐means (k = 2) clustering (Appendix Fig S1D–F), except for Nmt1‐GFP, where the threshold for each image was set manually. One image, where the autofluorescence of GFP‐negative cells deviated by more than three standard deviations from that of other images, was excluded. One additional image, where the cells had visibly moved during acquisition, was also excluded.

To subtract autofluorescence and other background, we averaged the fluorescence intensity per cell or nuclear volume for GFP‐negative cells in an image and subtracted that value from the fluorescence intensity per cell or nuclear volume of each GFP‐positive cell in the image. For a rough estimate of absolute concentration in nanomolar, we used our previous estimate of about 70 nM Mad3‐GFP in the cell nucleus (Heinrich *et al*, 2013) and normalized all background‐subtracted data to this value.

Even after background subtraction, we observed some variation of mean intensities between single images (Appendix Fig S1F), and we could not distinguish whether these differences were a consequence of sampling or came from conditions on the microscope stage while recording the image. We therefore opted to determine the coefficient of variation (CV = standard deviation/mean) for each protein not across all images, but instead for each image separately; Fig 2A shows the variation across images.

Generalized linear mixed models were used to test for differences in whole‐cell GFP concentration between wild‐type and codon‐optimized Mad1, Mad2, and Mad3 from the single‐cell measurements. A separate model was fit for each gene and included whole‐cell GFP concentration as the response variable and genotype (wild‐type versus codon‐optimized) as a categorical fixed effect predictor variable. Two nested random effects variables, experimental replicate and image, were also included in the model (random intercepts only). To meet the model assumptions of normality and constant variance, GFP concentration was transformed with a Box Cox transformation using “optim.boxcox” from the boxcoxmix package. Wild‐type and codon‐optimized genotypes were determined to have significantly different GFP concentrations if the 95% bootstrap confidence interval for the genotype coefficient excluded 0.

#### Quantification of GFP in single cells (2D segmentation and projection)

For experiments evaluating fluorescence signals after replacing the coding sequences of *mad1* ^+^, *mad2* ^+^, and *mad3* ^+^ (Appendix Fig S5), quantification was performed on projections, using 2D segmentation of cells. Cells were grown in minimal medium, collected by centrifugation from liquid cultures, mounted in medium on a slide, and brightfield and fluorescence images were collected immediately at room temperature. At least two slides were prepared and imaged for each strain. Images were recorded on a Zeiss AxioImager M1, using Xcite Fire LED illumination (Excelitas), a Zeiss Plan‐Apochromat 63x/1.40 Oil DIC objective, and an ORCA‐Flash4.0LT sCMOS camera (Hamamatsu) with Z sections spaced by 0.2 μm.

Cells were segmented based on an in‐focus brightfield image using YeaZ (Dietler *et al*, 2020). Falsely segmented cells (e.g., background, or cells falsely combined into one) were manually excluded in Fiji. Only cells in the center of the image, where fluorescence illumination was homogeneous, were included. Flatfielding was not performed. The brightfield images were systematically shifted relative to the fluorescence images and we corrected for that error. Quantification of signals was performed on an average projection of the 23 most in‐focus Z‐slices (covering 4.6 μm, which is slightly larger than the width of a typical *S. pombe* cell). For each image, the median extracellular background in the same central area of the image was subtracted.

#### Single‐molecule mRNA FISH

For quantification of mRNA by single‐molecule fluorescent in‐situ hybridization, cultures of asynchronously dividing cells were grown to a concentration of about 1 × 10^7^ cells/ml in EMM. Typically, 2 × 10^8^ cells were fixed with 4% paraformaldehyde for 30 min before being washed three times with ice‐cold Buffer B (1.2 M sorbitol, 100 mM potassium phosphate buffer pH 7.5) and stored at 4°C before digestion of the cell wall. Cells were resuspended in spheroplast buffer (1.2 M sorbitol, 0.1 M potassium phosphate, 20 mM vanadyl ribonuclease complex [NEB S1402S], 20 μM beta‐mercaptoethanol) and digested with 0.002% 100 T zymolyase (US Biological Z1005) for approximately 45–75 min. Zymolyase reaction was quenched when the addition of water to the cells resulted in around 50% lysed cells. Reactions were quenched with 3 washes of Buffer B. Cell pellets were resuspended in 1 ml of 0.01% Triton X‐100 in 1x PBS for 20 min and washed three times with Buffer B. For hybridization of probes, approximately 20–25 ng of CAL Fluor red 610 probes targeting ymEGFP or *mad2* ^+^, or Quasar 570 probes targeting *mad1* ^+^ were mixed with 2 μl each of yeast tRNA (Life Technologies) and Salmon sperm DNA (Life Technologies) per reaction. For two‐color FISH experiments, 20–25 ng of each probe were combined, resulting in ~ 50 ng of total FISH probes per reaction. Sequences of probes are given in Appendix Table S4. Buffer F (20% formamide, 10 mM sodium‐phosphate buffer pH 7.2; 45 μl per reaction) was mixed with the probe solution, heated at 95°C for 3 min, and allowed to cool to room temperature before mixing with Buffer H (4x saline‐sodium citrate (SSC) buffer, 4 mg/ml acetylated BSA, 20 mM vanadyl ribonuclease complex; 50 μl per reaction). Each sample of digested cells was divided into two reactions, each of which was resuspended in 100 μl of this hybridization solution. Resuspended cells were incubated at 37°C overnight. Cells were washed with 10% formamide/2x SSC followed by 0.1% Triton X‐100/2x SSC). For DAPI staining, cells were incubated in 1x PBS with 1 μg/ml DAPI for 10 min and washed once more with 1x PBS. Cell pellets were mixed with SlowFade Diamond Antifade Mountant (Thermo Scientific, S36972) and mounted on DEPC‐cleaned slides using #1.5 glass coverslips. Imaging was performed on a Zeiss AxioImager M1 equipped with Xcite Fire LED illumination (Excelitas), a Zeiss α Plan FLUAR 100x/1.45 oil objective, and an ORCA‐Flash4.0LT sCMOS camera (Hamamatsu). Images were acquired for 6 μm in Z separated by 0.2 μm steps for each channel. Images of labeled RNA were captured with either an mCherry filter or a “gold FISH” filter (Chroma, 49,304). Additional data on the cell and nucleus were captured with GFP, DAPI, and CFP filters. Images were dark noise‐subtracted and flatfield‐corrected. A custom FIJI macro, using trainable WEKA segmentation (Arganda‐Carreras *et al*, 2017), was used to create two‐dimensional outlines of cells by CFP autofluorescence and of corresponding nuclei by DAPI. For analyses with cytoplasmic or nuclear RNA counts (except the mad1/mad2 co‐localization experiment; Fig 1F), nuclei were re‐segmented in three dimensions using a FIJI macro adapted from https://github.com/haesleinhuepf/cca_benchmarking (Robert Haase, MPI‐CBG, Dresden). Analysis was limited to cells whose nuclei were entirely contained within the image stack. RNA spot analysis was performed in FISHquant (Mueller *et al*, 2013). Spots were initially detected based on an automatic intensity threshold and filtered with an additional manual threshold following the suggestions of the FISHquant documentation. A subset of cells in each image was cross‐checked manually for successful RNA spot detection.

To measure co‐localization of *mad1* ^+^ and *mad2* ^+^ mRNA, a two‐color FISH experiment was performed targeting *mad1* ^+^ with gene‐specific probes and *mad2* ^+^‐*ymEGFP* with ymEGFP probes. The three‐dimensional coordinates of each spot were recorded and corrected for relative chromatic aberration in Z. Distances were then calculated from each mRNA to its nearest neighbor of the other species within the same cell. To determine a distance cut‐off for classifying RNA molecules as either co‐localized or unpaired, the same two probe sets were used in another two‐color FISH experiment in which both probes targeted *mad1* ^+^ *‐ymEGFP*. Nearest‐neighbor distances were calculated in the same way, and the distribution of these distances was used to determine the co‐localization distance cut‐off value. This cut‐off was applied to the distances in the original experiment to classify each *mad1* ^+^ or *mad2* ^+^ mRNA molecule as co‐localized or unpaired.

To test if *mad1* ^+^ mRNA forms dimers, we used RNA FISH experiments to measure spot intensities and counts of RNA in the cytoplasm in strains with the following genotypes: (1) untagged *mad1* ^+^ expressed from the endogenous locus, (2) untagged *mad1* ^+^ expressed from the endogenous locus and *mad1* ^+^‐ymEGFP expressed from the exogenous leu1^+^ locus, (3) endogenous *mad1* ^+^ deleted and *mad1* ^+^‐*ymEGFP* expressed from the exogenous leu1^+^ locus, and (4) *mad1* ^+^‐*ymEGFP* and *mad3* ^+^‐*ymEGFP* expressed from the endogenous loci. All samples were hybridized with a combination of *mad1*‐ and *ymEGFP*‐targeting probes in two‐color FISH experiments. FISH probe spots were quantified separately for each imaging channel. Colocalized spots of different colors were then paired using the same co‐localization method as described for mad1/mad2 co‐localization above. Intensity analysis used the amplitude of the point spread function fit to each spot provided by FISHquant (Mueller *et al*, 2013). Using the intensity of each spot after background filtering, also provided by FISHquant, yielded the same result. All images quantified from the experiment for each probe set, regardless of genotype, gave consistent distributions of spot intensities, except for one image: while the distributions of spot intensities were qualitatively the same and the counts of spots were consistent with the other images, both its *mad1* ^+^ and its GFP probe spot intensities were shifted substantially lower compared with the other images (including another image from the same slide and another slide prepared from the same FISH sample). Thus, we decided to remove it from the analysis.

Single‐cell RNA counts from FISH experiments were fit with generalized linear mixed models. All models used a Poisson error distribution and natural log link function. The models included the fixed effect cell length and up to three nested random effects (when present): biological replicate (strain), experimental replicate, and microscopy image. Random effects included both random slopes and intercepts for all three variables. As the relationship between mean RNA count and cell length was approximately linear, cell length was natural log transformed. The transformed cell length was then centered so that a cell of average length had a value of zero.

To test for differences in mean RNA levels between genotypes, the categorical fixed effect variable genotype was added to the model. The interaction between cell length and genotype was also included if a likelihood ratio test comparing models with and without the interaction term showed that it improved the model's ability to explain the data significantly (*P* < 0.05). *P*‐values for the likelihood ratio test were obtained both by comparing the test statistic to a chi‐square distribution and generating a null distribution by bootstrapping (1,000 replicates) using the “PBmodcomp” function from the package pbkrtest. In all cases, the results were consistent between the two methods. Only a few models required the interaction term: comparison of wild‐type with codon‐optimized mad2 (whole cell, cytoplasmic, and nuclear RNA counts), comparison of wild‐type with codon‐optimized *mad2* + *ste13Δ* (cytoplasmic RNA only), and comparison of untagged and GFP‐tagged *mad1* ^+^.

Genotype coefficients and corresponding 95% bootstrap confidence intervals presented in the paper were exponentiated (e^) and represent the ratio of expected RNA levels between the two genotypes in each comparison. RNA levels were considered significantly different between genotypes if the exponentiated confidence interval excluded 1.

#### Assay for spindle assembly checkpoint function using *nda3‐KM311*

Strains expressing the tubulin mutant *nda3‐KM311* were grown in EMM (plus supplements required for auxotrophic mutations) at 30°C to a concentration of 0.5–1.0 × 10^7^ cells/ml. Cells were diluted with EMM to a final concentration of 7.5 × 10^5^ or 1.5 × 10^6^ cells/ml. 300 μl of each strain were loaded into a lectin‐coated Ibidi μ‐Slide glass‐bottom chamber and incubated about 1 h at 16°C on the microscope stage prior to imaging. Cells were imaged at 16°C on a DeltaVision Elite system with a PCO edge sCMOS camera (PCO), an Olympus 60x/1.42 Plan APO oil objective, and EMBL environmental chamber. Images were acquired every 5 min for GFP and mCherry over an 18‐h period using an “optical axis integration” (sum projection) over a 3.2 μm Z‐distance.

Plo1‐mCherry localizes to spindle‐pole bodies during mitosis and was used to identify cells in mitosis. Kinetochores cluster with spindle‐pole bodies in *S. pombe* interphase (Funabiki *et al*, 1993) and dot‐like GFP signals were therefore measured in the direct vicinity to Plo1‐mCherry. An area of the same size for each cell was used to capture the kinetochore signal and was also used to measure the intensity in the nucleoplasm for background subtraction. GFP intensities from multiple cells were aligned to the time point of Plo1‐mCherry appearance and averaged for each time point.

#### Assay for spindle assembly checkpoint function using alp7Δ

Cells were grown in EMM at 25°C to a concentration of 0.5–1.0 × 10^7^ cells/ml, diluted to 1.5 × 10^6^ cells/ml, and 300 μl of this dilution were loaded into a lectin‐coated ibidi μ‐Slide glass‐bottom chamber. Cells were incubated on the microscope stage at 30°C for 35 min before imaging. Images were acquired at 30°C every 55 s to 1.5 min for 2–3.5 h using an “optical axis integration” (sum projection) over a 3.6 μm Z‐distance. Cells were segmented based on the brightfield image using YeaZ (Dietler *et al*, 2020). All pixels within the cell were quantified and the 0.1^st^ percentile value was subtracted from the 99.9^th^ percentile value to obtain the “maximal intensity.” The localization of Plo1‐tdTomato to spindle‐pole bodies or Mad1‐GFP to kinetochores (Appendix Fig S4F) is reflected in higher maximal intensities. Time in mitosis was determined from a custom Matlab script that detects strong increases and decreases in signal. Some cells could not be analyzed in an automated fashion (e.g., due to overlapping other cells) and were analyzed manually. The analysis mode is reported in the source data.

#### RNA preparation

Asynchronous *S. pombe* cultures were grown to a final concentration of approximately 0.7–1.5 × 10^7^ cells/ml at 30°C in either EMM with 0.2 g/l leucine or YEA. 1 × 10^8^ cells were collected by centrifugation, washed once with deionized water, and immediately flash‐frozen in liquid nitrogen and stored at −80°C before processing. RNA was extracted by resuspending samples in 700 μl of TES buffer (10 mM Tris HCL pH 7.5, 10 mM EDTA, 0.5% SDS) and adding 700 μl of acidic phenol chloroform (Fisher Scientific). Samples were immediately vortexed for 20 s and incubated for 1 h at 65°C. Following incubation, samples were cooled on ice for 1 min, vortexed for an additional 20 s, and centrifuged for 15 min at 16,000 rcf at 4°C. The RNA was further purified by twice mixing the aqueous supernatant with 700 μl of acidic phenol chloroform and centrifuging the solution in a 5Prime Phase Lock Gel Heavy 2 ml tube (Andwin Scientific) at 16,000 rcf to separate the phases. Following overnight ethanol precipitation, samples were centrifuged at 16,000 rcf for 10 min at 4°C and washed with one equivalent of 70% ethanol before additional centrifugation. Samples were left to air‐dry at room temperature and resuspended in nuclease free water before quantification. 50 μg of total RNA was subjected to DNase treatment (Roche, 10776785001) followed by ethanol precipitation.

#### Quantitative PCR (qPCR)

For quantitative PCR (qPCR), 1 μg of DNase‐treated total RNA was subjected to Superscript IV cDNA synthesis using oligo d(T)_20_ primers. Transcript abundance was quantified on a QuantStudio 6 Real‐Time PCR system using SYBR® Green PCR Master Mix (ThermoFisher) and gene‐specific primers (Appendix Table S5). To estimate relative expression, raw Ct values (2–3 technical replicates per sample) were averaged and normalized according to the following formula (Pfaffl, 2001; Hellemans *et al*, 2007):

$$\text{Relative Expression}=\frac{{\left(\text{efficienc}y_{\text{target}}+1\right)}^{\left(Ct_{\text{target control}}\;-\;Ct_{\text{target sample}}\right)}}{{\left(\prod {\left(\text{efficienc}y_{\text{reference}}+1\right)}^{\left(Ct_{\text{reference control}}\;-\;Ct_{\text{reference sample}}\right)}\right)}^{\frac{1}{n}}}$$

where “target” is the mRNA of interest, “reference” is the reference gene, “sample” is the sample of interest, and “control” is the control sample being normalized to. The denominator is the geometric mean of the reference genes (*act1* ^+^ and *cdc2* ^+^), and efficiencies were estimated from the slopes of four‐step, serial 1:5 dilution standard curves.

#### Determination of mRNA half‐life

The mRNA half‐life measurement procedure was adapted from published protocols (Duffy *et al*, 2015; Chan *et al*, 2018). Asynchronous *S. pombe* cultures were grown to a final density of approximately 0.7–0.9 × 10^7^ cells/ml at 30°C in EMM with 0.45 mM uracil and 1.5 mM leucine before collection. 4‐thiouracil (4tU) in DMSO (Chem‐Impex International, CMX‐21484) was added at 5 mM final concentration (Eser *et al*, 2016). Cells (5 × 10^7^ cells per sample) were collected by centrifugation, immediately flash‐frozen in liquid nitrogen, and stored at −80°C before processing. Samples were collected from the culture before addition of 4tU (time = 0) and at a series of time points after. For use as a spike‐in control, *S. cerevisiae* cultures were grown to a final density of 1.4–3.7 × 10^7^ cells/ml in YPD at 30°C, flash‐frozen, and stored at −80°C.

RNA extraction was performed as above except flash‐frozen samples were initially resuspended in 600 μl TES buffer and 100 μl of resuspended *S. cerevisiae* cells (approximately 5 × 10^7^ cells) were added as a spike‐in control. Care was taken to add the same amount of *S. cerevisiae* cells to all sample of a time series. After extraction, 200 μg of total RNA was subjected to DNase treatment. Following DNase treatment, 70 μg of RNA was biotinylated with MTSEA biotin‐XX (Biotium, 90066) as previously described (Duffy *et al*, 2015; Chan *et al*, 2018). 50 μg of biotinylated RNA was subjected to oligo d(T) selection using oligo d(T)_25_ magnetic beads (NEB, S1419S), substituting SDS and NaCl for the recommended LiDS and LiCl. For streptavidin selection of biotinylated RNA, 500 ng of the oligo d(T) selected mRNA was used. 25 μl of MyOne Streptavidin C1 Dynabeads (ThermoFisher, 65001) were washed with 75 μl of 0.1 M NaOH two times, followed by a single 0.1 M NaCl wash, and two additional washes with Buffer 3 (10 mM Tris HCl pH 7.4, 10 mM EDTA, 1 M NaCl). Streptavidin beads were blocked by resuspension in 50 μl of Buffer 3 and 5 μl of 50x Denhardt's reagent. Beads were incubated for 20 min with gentle agitation. Following blocking, beads were washed with 75 μl of Buffer 3 four times and resuspended in 75 μl of Buffer 3 with 4 μl of 5 M NaCl. 500 ng of mRNA was added to the beads and gently agitated for 15 min. Following incubation, beads were washed with 75 μl of Buffer 3 prewarmed to 65°C, once with Buffer 4 (10 mM Tris–HCl pH 7.4, 1 mM EDTA, 1% SDS) and twice with 10% Buffer 3. All flow‐through was pooled before addition of 20 μg of linear acrylamide (ThermoFisher) followed by ethanol precipitation.

Expression relative to the time = 0 sample was quantified using qPCR as described above except 50 ng of recovered unlabeled mRNA was used in the Superscript IV cDNA synthesis reaction and a single reference gene (*S. cerevisiae* ACT1 from the spiked in *S. cerevisiae* cells) was used to normalize expression.

To estimate mRNA half‐lives (HL), several different exponential decay models (adapted from (Chan *et al*, 2018)) were initially fit to each time series using nonlinear least squares regression:

fit1=2−tHL

fit2=efficiency*2−tHL+1−efficiency

fit3=mRNA0*2−tHL

Fit 1 is a simple one‐phase exponential decay model. Fit 2 incorporates an efficiency parameter to accommodate that mRNA levels may not decay to zero (Chan *et al*, 2018). Finally, to accommodate the effects of noninstantaneous labeling by 4tU on the decay curve, the fit 3 model was fit without the time = 0 measurements and instead allowed the mRNA level at time = 0 to be estimated as a separate free parameter (mRNA_0_). Qualitatively, fit 3 consistently fit the time series best, but all models yielded similar results. To test for statistically significant differences in mRNA half‐lives between *ste13* ^+^ and *ste13Δ* genotypes, we therefore proceeded with the fit 3 model, which was linearized, resulting in the equation

$$\ln\left({\text{mRNA}}_{t}\right)=\ln\left({\text{mRNA}}_{0}\right)+\frac{-\ln\left(2\right)}{\mathrm{HL}}t$$

and was fit to the combined *ste13* ^+^ and *ste13Δ* time series data for each gene (excluding time = 0) using a general linear mixed model. In the model, natural log transformed relative mRNA expression was modeled as a function of the continuous fixed effects variable time (minutes since 4tU addition), the categorical fixed effect genotype (*ste13* ^+^ vs. *ste13Δ*), the interaction of time and genotype, and the random effect experimental replicate. The random effect included both random slopes and intercepts to allow the decay rate to vary across experimental replicates.

In the model, half‐life is related to the change in expression with respect to time by the formula $\mathrm{HL}=-\ln\left(2\right)/\text{slope}$, where slope is the time coefficient for the genotype coded 0 and the sum of the time and interaction coefficients for the genotype coded 1. To simplify the extraction of half‐life estimates, models were fit with the genotype coded both ways: first *ste13* ^+^ = 0 and then *ste13Δ* = 0. Half‐life estimates for *ste13* ^+^ and *ste13Δ* genotypes were derived from the version of the model in which the genotype of interest was coded zero using the formula $-\ln\left(2\right)/\left[\text{time coefficient}\right]$. Similarly, the difference in half‐life between the two genotypes was estimated with the formula:

$$\frac{\ln\left(2\right)*\left[\text{interaction coefficient}\right]}{\left(\left[\text{time coefficient}\right]*\left[\text{time coefficient}+\text{interaction coefficient}\right]\right)}$$

Significance of the change in half‐life due to the deletion of *ste13* ^+^ was measured two ways. If the 95% bootstrap confidence interval for the interaction coefficient excluded 0, the slopes of expression with respect to time in the model, and thus half‐lives, were considered to be different. Similarly, if the 95% bootstrap confidence interval for the half‐life difference excluded 0, the change was considered significant.

#### Codon usage bias calculations

The “Codon occurrence to mRNA Stability Correlation coefficient” (CSC) for each codon was determined as in Presnyak *et al* (2015) by calculating a Pearson's correlation coefficient between the frequency of occurrence of individual codons in *S. pombe* mRNAs and the half‐lives of these mRNAs. Coding sequences for *S. pombe* (protein‐coding genes, excluding dubious and transposons) were downloaded from Pombase (ASM294v2.62, Release date 2017‐01‐30) (Lock *et al*, 2019). From this list, we excluded “Genome location: mitochondrial,” “Genome location: mating_type_region,” and “sequence error in genomic data” (PBO:0000129). Five genes lacking start or stop codons were additionally excluded, resulting in a final list of 5,016 genes. We used mRNA half‐life data from either Hasan *et al* (2014) or Eser *et al* (2016), which are the most recent and comprehensive datasets for *S. pombe*. Of the 5,016 genes in our list, 4,615 were measured in at least one study and 3,900 in both. Both studies used metabolic labeling and the half‐lives correlate with each other (Pearson's correlation coefficient 0.50; Spearman's rank correlation 0.81).

A previous study (Harigaya & Parker, 2016) had used the Spearman's correlation coefficient to determine CSC values for *S. pombe*, because of outliers in the half‐life data. We instead removed outliers from the half‐life data and used the Pearson's correlation coefficient. A comparison between the different strategies is shown in Appendix Fig S4C and D. Our criteria for removing outliers were as follows: (i) a value that was more than 10 interquartile ranges above the upper quartile (which removed three genes, based on their value in the Eser *et al*, 2016 data) and (ii) a deviation in rank position of > 2,500 between the two datasets (which removed 13 genes). After the removal of outliers, the Pearson's correlation coefficient between the two mRNA half‐life datasets was 0.80, the Spearman's rank correlation 0.82. Using either the Hasan *et al*, 2014 or the Eser *et al*, 2016 half‐life data for CSC calculation yielded highly similar results (Appendix Fig S4C and D). When not otherwise indicated, CSC values obtained from Eser *et al*, 2016 (the more recent study) were used. The CSC_g_ value for each gene was determined as the arithmetic mean of all codons, excluding the stop codon.

The percentage of optimal codons based on the “classical translational efficiency” (cTE) used the optimality table for *S. pombe* from Pechmann & Frydman (2013). For tAI (tRNA adaptation index), we used the values determined by Tuller *et al* (2010) and also reported in Pechmann & Frydman (2013). The tAI_g_ value for each gene was determined as the geometric mean of all codons, excluding the stop codon.

CSC values for budding yeast were taken from Carneiro *et al* (2019) and only values derived from mRNA half‐life measurements within the last 10 years were included (Becskei: Baudrimont *et al*, 2017; Coller: Presnyak *et al*, 2015; Cramer: Sun *et al*, 2012; Gresham: Neymotin *et al*, 2014; Struhl: Geisberg *et al*, 2014; Weis: Munchel *et al*, 2011). CSC values derived from the Struhl study are shown in gray in Appendix Fig S7, since they differed from the ones based on the other mRNA half‐life datasets (Carneiro *et al*, 2019). CSC values for human cells were taken from Wu *et al* (2019), Narula *et al* (2019), and Forrest *et al* (2020). CSC values from the Forrest study are shown in gray in Appendix Fig S7, as they were based on fewer mRNA half‐life data and differed from the other two studies in their trend. The multiple CSC values from Wu *et al* (2019) and Narula *et al* (2019), respectively, were averaged, and the mean value obtained in each study was used. Mad1 and Mad2 sequences from opisthokonts were taken from Vleugel *et al* (2012). The human Mad1 sequence was swapped for the canonical isoform (UniProt, Q9Y6D9, MD1L1_HUMAN), the *S. pombe* Mad1 sequence was shortened N‐terminally by 13 amino acids to start with what is now considered the correct start codon (pombase.org). All sequences shorter than 600 amino acids were omitted for Mad1. Protein sequences were aligned using MAFFT (G‐INS‐I, using DASH and Mafft‐homologs [100 homologs, E = 1e‐30]) (Katoh *et al*, 2019). Sequences from all species other than *S.c*., *S.p*., and *H.s*. were deleted for the display of conservation shown in Appendix Fig S7. The moving average of CSC values across nine codons was plotted along the length of the aligned sequence. The null distribution of the moving average was obtained by randomizing the codon order 10,000 times. Observed values that deviated by more than two standard deviations from the null mean were marked with filled circles.

#### Gene expression model—Simulations and theoretical predictions

Protein noise predictions (Fig 2C; Appendix Fig S2B and C) were made by assuming a constitutively active promoter, and only considering stochastic mRNA and protein synthesis and degradation and ignoring cell growth and division. The coefficient of variation (CV = standard deviation/mean) for protein is calculated as:

$${\mathrm{CV}}_{P}=\sqrt{\frac{1}{P}+\frac{1}{M}\frac{k_{\text{degP}}}{\left(k_{\text{degP}}+k_{\text{degM}}\right)}}$$

where *P* is the mean protein number per cell, *M* the mean mRNA number per cell, *k*_degM_ the mRNA degradation rate, and *k*_degP_ the protein degradation rate (Swain, 2004). For the predictions in Appendix Fig S2C, we assumed a mean protein number of 6,000 per cell, mean mRNA numbers of 1 to 1,000, and we varied RNA degradation rate in a range corresponding to half‐lives of 1 to 60 min, and protein degradation rate in a range corresponding to half‐lives of 15 to 600 min, which we consider a physiologically plausible range. Predictions were excluded when mRNA synthesis or protein synthesis rates became unrealistically high. We assumed this to be the case when mRNA synthesis rate was higher than 25 min^−1^ or protein synthesis rate higher than 20 mRNA^−1^ min^−1^. Assuming a gene with characteristics similar to a SAC gene (mean protein number = 6,000, mean mRNA number = 3.5, protein half‐life = 360 min, mRNA half‐life = 4 min) yields a CV prediction of 0.0575. In the figure, we labeled CV predictions less than 0.06 in light gray (low noise) and those equal or higher than 0.06 in dark gray (high noise).

The stochastic simulation of mRNA and protein numbers (Fig 2B) used the same simple underlying model and the Gillespie algorithm in a Matlab script written by Daniel Charlebois and available on MathWorks (“Gillespie's Direct Method Stochastic Simulation Algorithm“).

#### Statistical tests

Data processing performed in R used the packages tidyverse (Wickham *et al*, 2019), alphashape3d, boxcoxmix, broom, broom.mixed, DescTools, geometry, Irescale, mclust, nabor, plyr, readxl, rgl, sf, shotGroups, and spatstat. Statistical tests were performed in Prism (GraphPad), or in R using the packages lme4 (Bates *et al*, 2015), MASS, pbkrtest, and stats. Figure plots were generated in Prism (GraphPad) or in R using the packages Cairo, cowplot, egg, ggplot2, gridExtra, plotly, and lemon.

General linear mixed models and generalized linear mixed models were fit using the functions “lmer” and “glmer,” respectively, from the lme4 package. Default function settings were used except for the optimizer in “glmer,” which was set to “bobyqa.” Bootstrapping using the function “bootMer” (10,000 replicates, lme4 package) was used to obtain 95% confidence intervals for fixed effects model coefficients and mRNA half‐life estimates, and 95% confidence bands for predicted regression curves. Wilcoxon rank sum tests, and *t*‐tests were performed using “wilcox.test” and “t.test,” respectively, from the package stats. Poisson distributions were fit to data frequency distributions using “fitdistr” from the package MASS.

Sample sizes were not predetermined. Blinding was not performed, as most analyses were run in an automated fashion.

## Data availability

This study includes no data deposited in external repositories.

## Author contributions

**Eric Esposito:** Conceptualization; formal analysis; investigation; visualization; writing – original draft. **Douglas E Weidemann:** Conceptualization; formal analysis; investigation; visualization; writing – original draft. **Jessie M Rogers:** Formal analysis; investigation; visualization; writing – review and editing. **Claire M Morton:** Formal analysis; investigation. **Erod Keaton Baybay:** Investigation; methodology; writing – review and editing. **Jing Chen:** Investigation; methodology; writing – review and editing. **Silke Hauf:** Conceptualization; formal analysis; supervision; funding acquisition; investigation; visualization; writing – original draft.

## Disclosure and competing interests statement

The authors declare that they have no conflict of interest.

## Supporting information



Appendix

Expanded View Figures PDF

Source Data for Expanded View and Appendix

Source Data for Figure 1

Source Data for Figure 2

Source Data for Figure 3

Source Data for Figure 4

Source Data for Figure 5

Source Data for Figure 6

Source Data for Figure 7
