# Supplementary material for: Mitotic checkpoint gene expression is tuned by codon usage bias
Source: EMBO J. 2022 Jul 11;41(15):e107896. doi: 10.15252/embj.2021107896 (PMC9340482; doi:10.15252/embj.2021107896)
Supplement: Supplementary file 1 — Appendix [file EMBJ-41-e107896-s004.pdf]

Appendix

Mitotic checkpoint gene expression is tuned by coding sequences

Esposito, Weidemann, et al.

Figure S1. Image analysis using the Pomegranate pipeline to determine GFP intensities and noise 2
Figure S2. Additional data on Mad1, Mad2, and Mad3 protein concentrations and half-life 3
Figure S3. Codon optimality of *mad1*, *mad2*, and *mad3* before and after codon-optimization 4
Figure S4. Additional data on Mad1-co-GFP protein half-life and function 6
Figure S5. Sequences surrounding the *mad1*$^{+}$ coding region are insufficient for proper expression............................ 8
Figure S6. Assessing dimerization of Mad1 C-terminal fragments in yeast cell extract and in vitro translation............ 9
Figure S7. CSC along Mad1 and Mad2 in *S. cerevisiae*, *S. pombe*, and *H. sapiens* and synonymous mutations observed in cancer 10
Table S1 - Yeast strains 11
Table S2 - sgRNA targeting sequences 13
Table S3 - codon-optimized SAC gene sequences... 14
Table S4 - FISH probes.. 15
Table S5 - qPCR primers 16
Table S6 - Mad1 sequences for in vitro transcription and translation 17

Figure S1

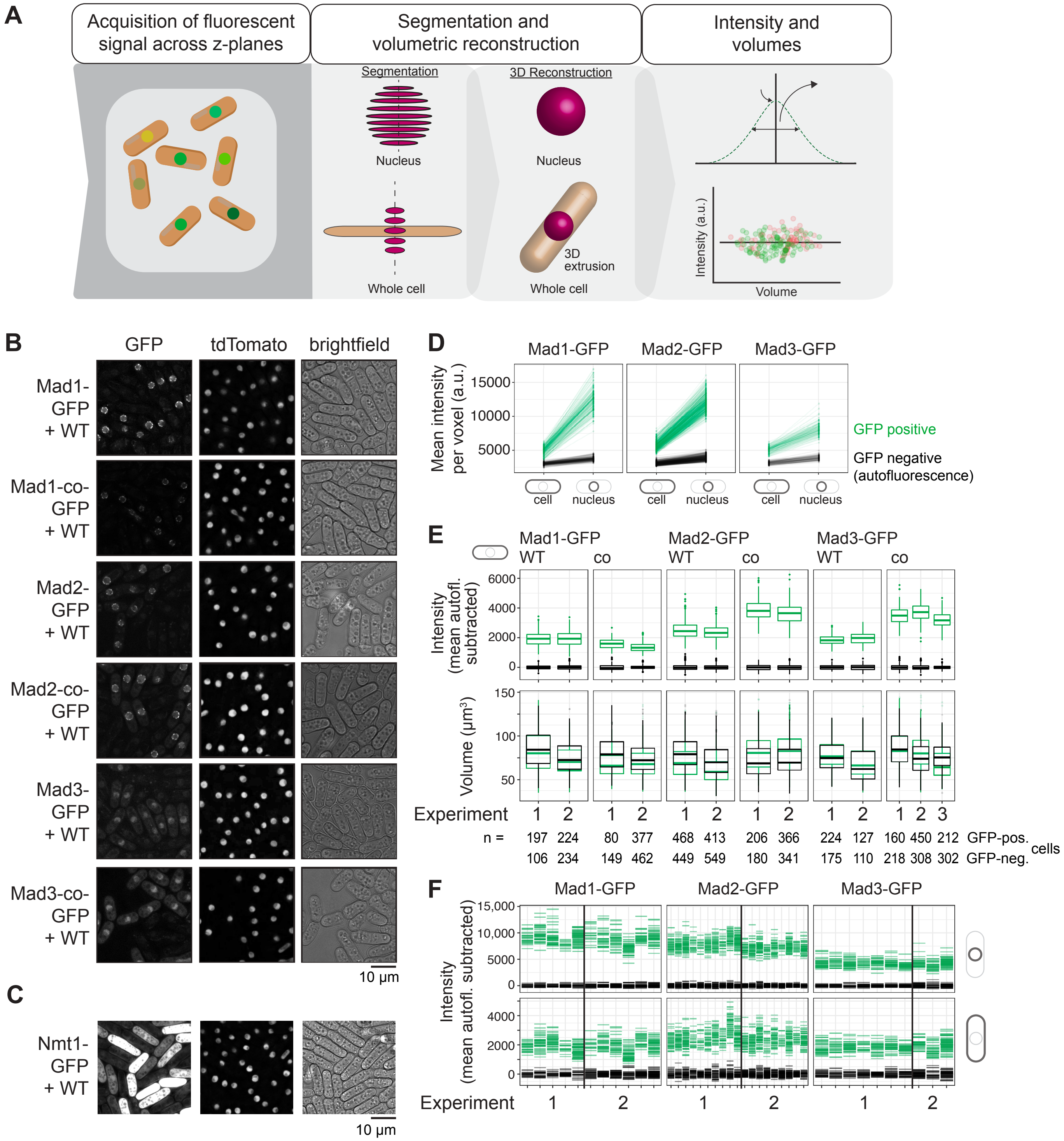

Figure S1. Image analysis using the Pomegranate pipeline to determine GFP intensities and noise. **(A)** Schematic overview of the Pomegranate pipeline (Baybay *et al.*, 2020): cells are segmented in two dimensions (2D) based on the brightfield image, nuclei are segmented in three dimensions (3D) based on tdTomato-NLS signal. To collect signal from the entire cell, the most in-focus 2D cell segmentation is extended into 3D by spherical extrusion. Signals are averaged from across all z-sections to obtain cellular and nuclear intensities. **(B, C)** Example pictures from live-cell imaging. The strains to be quantified are mixed with wild-type cells (WT, not expressing GFP) to subtract autofluorescence. Cells are trapped in a microfluidics channel. **(D)** GFP-expressing (green) and GFP-negative wild-type cells (black) are separated based on their distinct intensities in the green channel (a.u. = arbitrary units). For checkpoint proteins, k-means clustering with k = 2 was used. For Nmt1-GFP, the populations were split manually. **(E)** Volumes between GFP-positive (green) and GFP-negative (black) cells are similar, but intensities differ. For intensity, the mean signal intensity of the GFP-negative cells in each image was subtracted. (WT = wild-type coding sequence, co = codon-optimized coding sequence) **(F)** Fluorescence intensities of GFP-positive (green) and GFP-negative (black) cells in different images and experiments to demonstrate variability. Black vertical lines separate experiments on different days. Top: nuclear intensities; bottom: whole-cell intensities.

Figure S2

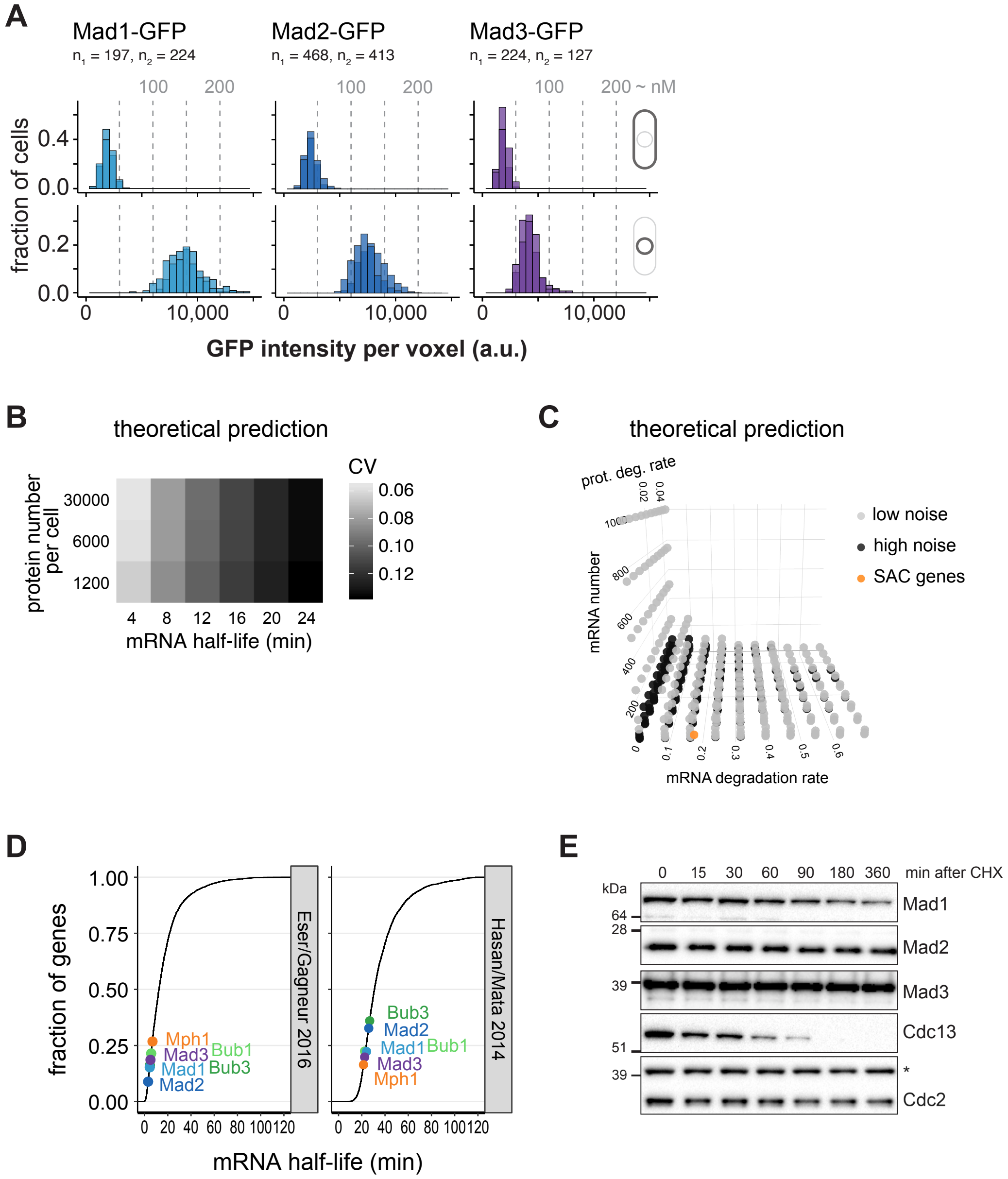

Figure S2. Additional data on Mad1, Mad2, and Mad3 protein concentrations and half-life.
(A) Concentration of GFP measured by live-cell imaging in whole cells (top) or nuclei (bottom). Two replicate experiments are overlaid. Estimates for concentration in nM are derived from comparison to previous absolute quantification of a *mad3*$^{+}$-GFP<<kanR strain and are very rough estimates only. **(B)** Theoretical prediction for the coefficient of variation (CV = std / mean) of the protein number per cell, assuming different mRNA half-lives, using the same underlying model as in Figure 1B and C. Protein half-life was set to 6 hours and protein synthesis rate was adjusted to reach each specified mean on the y-axis. Synthesis rate for mRNA was adjusted to maintain a mean mRNA number per cell of 3.5. **(C)** Simulated protein noise for different mRNA numbers, mRNA degradation rates and protein degradation rates. Noise was labeled as low when it was similar or lower than that of SAC genes and high otherwise. The mRNA degradation rate was varied in a range corresponding to half-lives of 1–60 minutes. The protein degradation rate was varied in a range corresponding to half-lives of 15–600 minutes. Missing points (e.g. high mRNA numbers at high mRNA degradation rates) are not included, because they would require non-physiologically high transcription or translation rates. The position where SAC genes are found in this grid is marked in orange. **(D)** Cumulative distribution of the mRNA half-lives of protein-coding *S. pombe* genes, measured by Eser *et al.* (2016) or Hasan *et al.* (2014). Position of spindle assembly checkpoint genes marked in color. **(E)** Immunoblot of protein extracts harvested at the indicated times after translation shut-off by cycloheximide. One of the experiments quantified in Figure 2E. Asterisk indicates a cross-reaction of the anti-Cdc2 antibody.

Figure S3

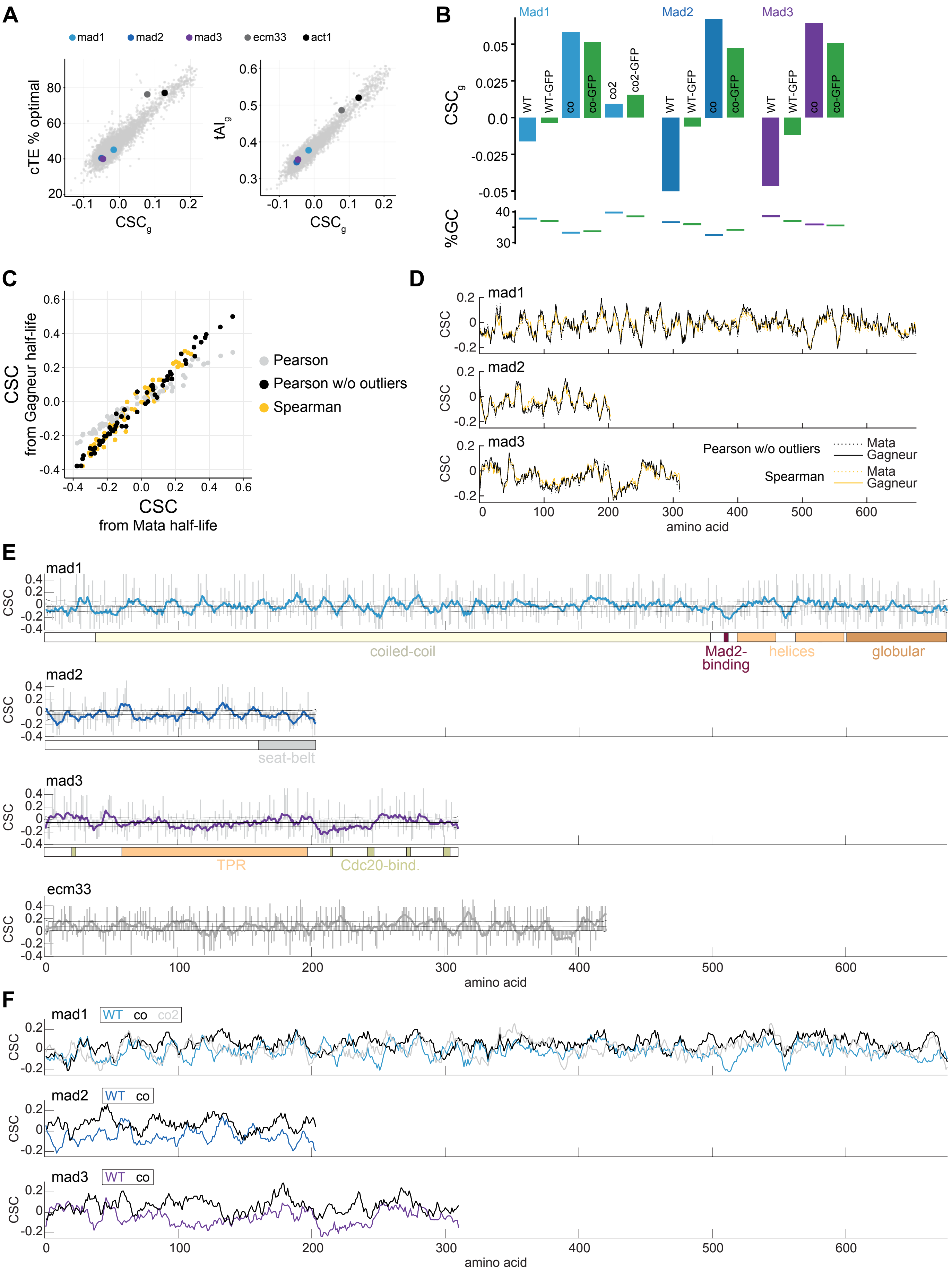

A
mad1 mad2 mad3 ecm33 act1
cTE % optimal
tAI$_g$
CSC$_g$
B
Mad1 Mad2 Mad3
WT WT-GFP co co-GFP co2 co2-GFP
CSC$_g$
%GC
C
CSC from Gagneur half-life
CSC from Mata half-life
Pearson
Pearson w/o outliers
Spearman
D
mad1 mad2 mad3
CSC
Pearson w/o outliers Mata Gagneur
Spearman Mata Gagneur
amino acid
E
mad1
coiled-coil
Mad2-binding
helices
globular
mad2
seat-belt
mad3
TPR
Cdc20-bind.
ecm33
amino acid
F
mad1 WT co co2
mad2 WT co
mad3 WT co
amino acid

Figure S3. Codon optimality of *mad1*, *mad2*, and *mad3* before and after codon-optimization.
(A) Comparison between CSC_g and other measures of codon optimality across protein-coding *S. pombe* genes. Genes relevant in this study are highlighted. **(B)** CSC_g and GC content (%GC) for *mad1*, *mad2* and *mad3* before and after codon-optimization, and without or with taking the GFP tag into account. **(C)** CSC of the 61 amino acid-coding codons derived using mRNA half-life data from either the Mata group (Hasan *et al.*, 2014) or the Gagneur group (Eser *et al.*, 2016), and determined using different correlation methods (color), as explained in the methods section. **(D)** Moving average of the CSC across 9 codons along the length of each SAC gene, using codon CSC values derived from different datasets or with different correlation methods as in (C). **(E)** CSC value of each codon (grey bars) and moving average across 9 codons (colored line) along the length of each gene. For the SAC genes, functional and structural motifs are shown at the bottom. The middle black line represents the mean CSC across the gene (=CSC_g). The black lines above and below were obtained by randomly permuting the CSC values along the gene 10,000-times and determining the moving average across 9 codons (as for the original data). Shown is +/- 1 standard deviation of this randomized data. **(F)** Moving average of the CSC across 9 codons along the length of each SAC gene for both the wild-type and codon-optimized versions.

Figure S4

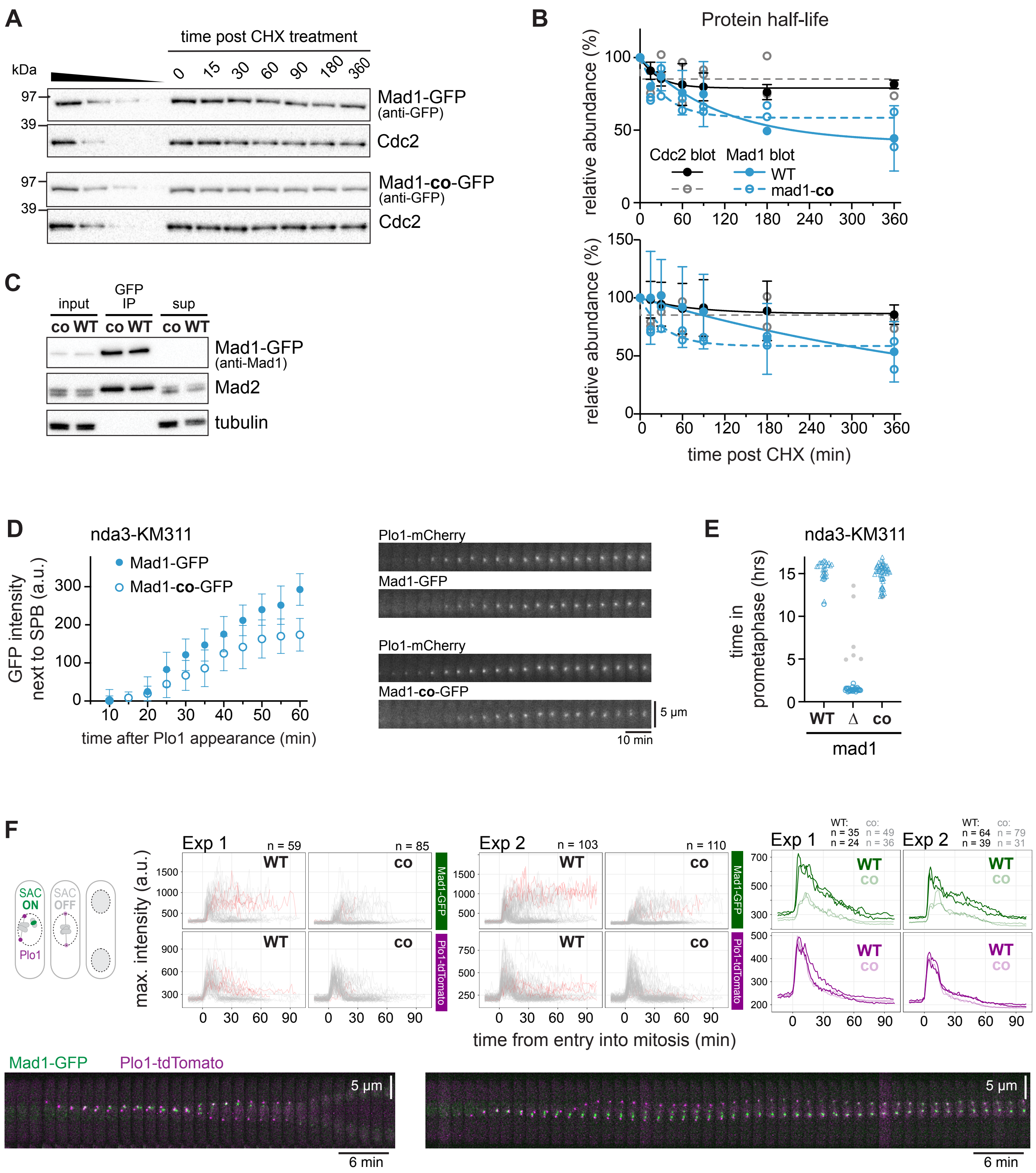

A
time post CHX treatment
kDa
0 15 30 60 90 180 360
97
39
Mad1-GFP (anti-GFP)
Cdc2
Mad1-co-GFP (anti-GFP)
B
Protein half-life
relative abundance (%)
Cdc2 blot
Mad1 blot
WT
mad1-co
time post CHX (min)
C
input
GFP IP
sup
co WT
Mad1-GFP (anti-Mad1)
Mad2
tubulin
D
nda3-KM311
Mad1-GFP
Mad1-co-GFP
GFP intensity next to SPB (a.u.)
time after Plo1 appearance (min)
Plo1-mCherry
5 µm
10 min
E
time in prometaphase (hrs)
WT Δ co
mad1
F
SAC ON
SAC OFF
Plo1
Exp 1
Exp 2
n = 59
n = 85
n = 103
n = 110
max. intensity (a.u.)
Mad1-GFP
Plo1-tdTomato
time from entry into mitosis (min)
WT: n = 35 n = 24
co: n = 49 n = 36
WT: n = 64 n = 39
co: n = 79 n = 31
6 min

Figure S4. Additional data on Mad1-co-GFP protein half-life and function.
(A) Immunoblot of protein extracts at the indicated times after translation shut-off by cycloheximide. One of the experiments quantified in (B). The first four lanes contain a 1:1 dilution series of the 0 min sample. Mad1-GFP and Mad1-co-GFP were probed with anti-GFP. Cdc2 serves as control. **(B)** Top: Protein abundances after translation shut-off with cycloheximide (n = 3 experiments for wild-type (WT) cells, error bars = std; n = 2 experiments for mad1-co expressing cells). Lines indicate fit to a one-phase exponential decay. Extracts were probed for Mad1 and Cdc2. A representative immunoblot is shown in (A). Bottom: Same data, but now including one experiment for Mad1 WT cells, where levels for both Mad1 and Cdc2 at time points 15 min to 180 min were higher than at the 0 min time point. **(C)** Anti-GFP immunoprecipitation (IP) of Mad1-GFP (WT) and Mad1-co-GFP. Input, IP, and supernatant after IP (sup) were probed with antibodies against Mad1, Mad2 and tubulin. **(D)** The accumulation of Mad1-GFP at kinetochores in early mitosis was quantified in cells expressing the cold-sensitive tubulin mutant *nda3-KM311* and *plo1*$^{+}$*-mCherry*, imaged at the restrictive temperature of 16ºC. Mad1-GFP signals adjacent to spindle pole bodies (marked with Plo1-mCherry) were interpreted as kinetochore localization (a.u. = arbitrary units; error bars = s.d.; n = 13 and 12 cells). One out of two experiments is shown. **(E)** Cells expressing the cold-sensitive tubulin mutant *nda3-KM311*, *plo1*$^{+}$*-mCherry*, and the indicated versions of *mad1* were analyzed by live-cell imaging at the restrictive temperature of 16ºC, similar to the experiment in (D). The time that each cell spent in prometaphase was determined by localization of Plo1 to spindle pole bodies (circle). Cells for which mitosis time could be measured precisely are indicated by open, blue circles, cells that had not yet exited mitosis when filming stopped by open, blue triangles, and cells that died during mitosis by filled, gray circles. n = 29, 32 and 53 cells. **(F)** Additional data for the experiment in Fig 6F. Live-cell imaging of *alp7*Δ cells expressing wild-type (WT) or codon-optimized (co) *mad1*-GFP as well as *plo1*-tdTomato. Maximum GFP and tdTomato signals in cells were quantified in two experiments (using different strains with identical genotype). Number of cells is shown at the top. Maximum Mad1-GFP signal in the cell reports on Mad1 kinetochore localization (and hence spindle assembly checkpoint activation); maximum Plo1-tdTomato signal in the cell reports on Plo1 spindle pole body localization (and hence on whether the cell is in mitosis or not). Entry into mitosis was judged by an increase in maximal Plo1 signal, and curves were aligned to that time point. Left and middle panels show single cells; cells that had not exited mitosis by the end of the movie are shown in red, all others in gray. Panel on the right shows the average across all cells. Each experiment had two rounds of imaging, which are shown separately. When values were not available for a given time point for a given cell (e.g. because the movie had ended after the cell exited mitosis), the minimum signal observed for this cell was used in the averaging. Kymographs of representative cells are shown at the bottom. The cell on the left silences the spindle assembly checkpoint and exits mitosis; the cell on the right maintains spindle assembly checkpoint activation until the end of the movie.

Figure S5

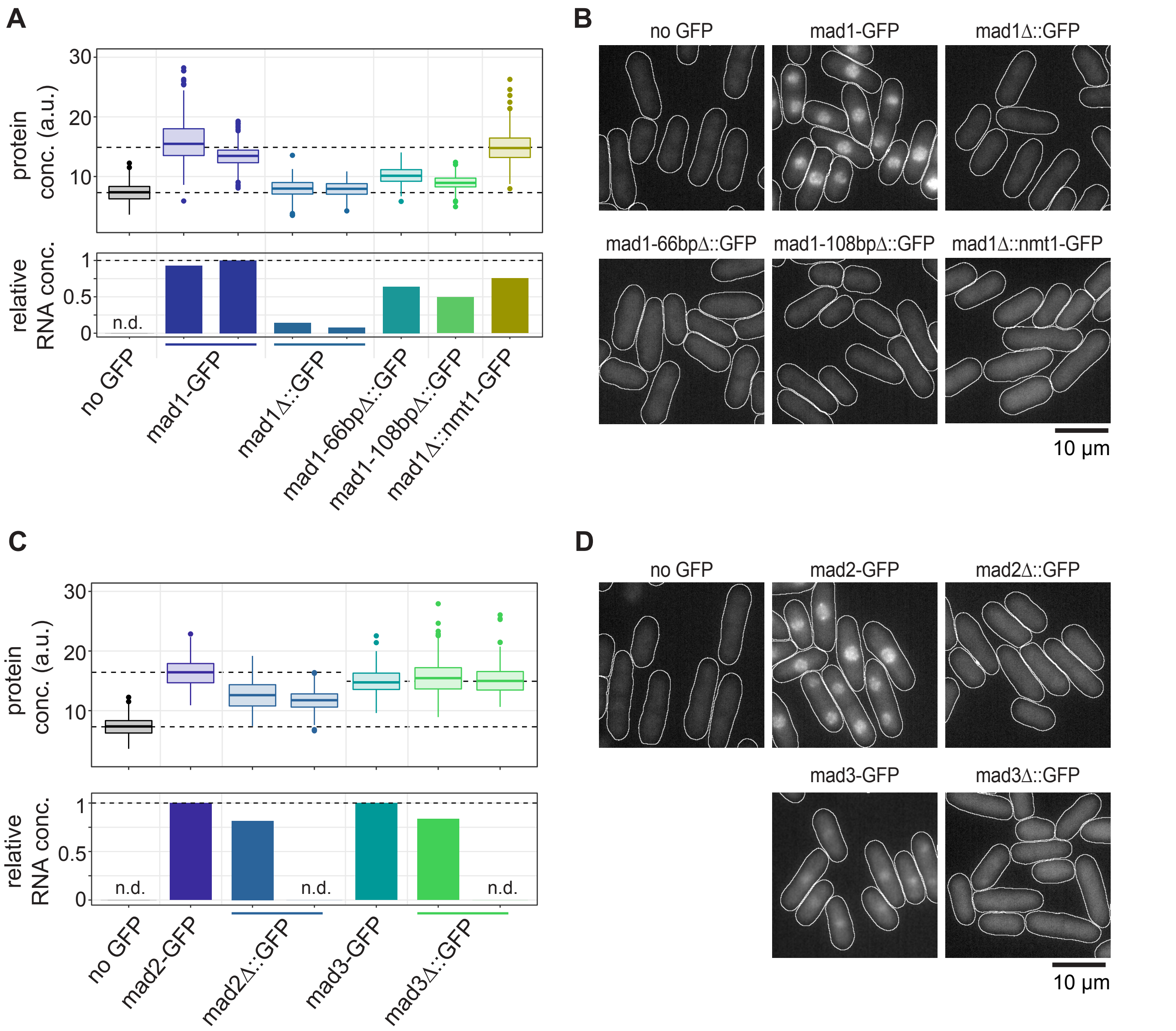

Figure S5. Sequences surrounding the *mad1*$^+$ coding region are insufficient for proper expression.
(A) The indicated strains were tested for GFP expression by qPCR (bottom panel) and by live-cell microscopy (top panel; a.u. = arbitrary units). *Mad1* was either fused to GFP *(mad1*-GFP), or the coding sequence of *mad1*$^+$ was replaced with GFP *(mad1Δ::*GFP), replaced with the 5' region of *mad1*$^+$ (66 bp or 108 bp) followed by GFP, or replaced with *nmt1*$^+$-GFP *(mad1Δ::nmt1*-GFP). A strain not expressing any GFP was used as reference. Boxplots show median and interquartile range (IQR); lines extend to values no further than 1.5 times the IQR from the first and third quartile, respectively. For microscopy: no GFP: n = 634 cells; *mad1*-GFP: n = 525 and 391; *mad1Δ::*GFP: n = 497 and 360; *mad1-66bpΔ::*GFP: n = 293; *mad1-108bpΔ::*GFP: n = 173; *mad1Δ::nmt1*-GFP: n = 419 cells. **(B)** Representative live-cell microscopy images from the experiment in (A). Cell shapes are outlined in white. **(C)** Similar to (A), but for strains in which *mad2*$^+$ or *mad3*$^+$ were either fused to GFP *(mad2*-GFP, *mad3*-GFP), or the coding sequence was replaced with GFP *(mad2Δ::*GFP, *mad3Δ::*GFP). The strain not expressing any GFP is the same as in (A). For microscopy: no GFP: n = 634 (same strain and experiment as in A); *mad2*-GFP: n = 333; *mad2Δ::*GFP: n = 299 and 358; *mad3*-GFP: n = 355; *mad3Δ::*GFP: n = 608 and 297 cells. **(D)** Representative live-cell microscopy images from the experiment in (C). Cell shapes are outlined in white.

Figure S6

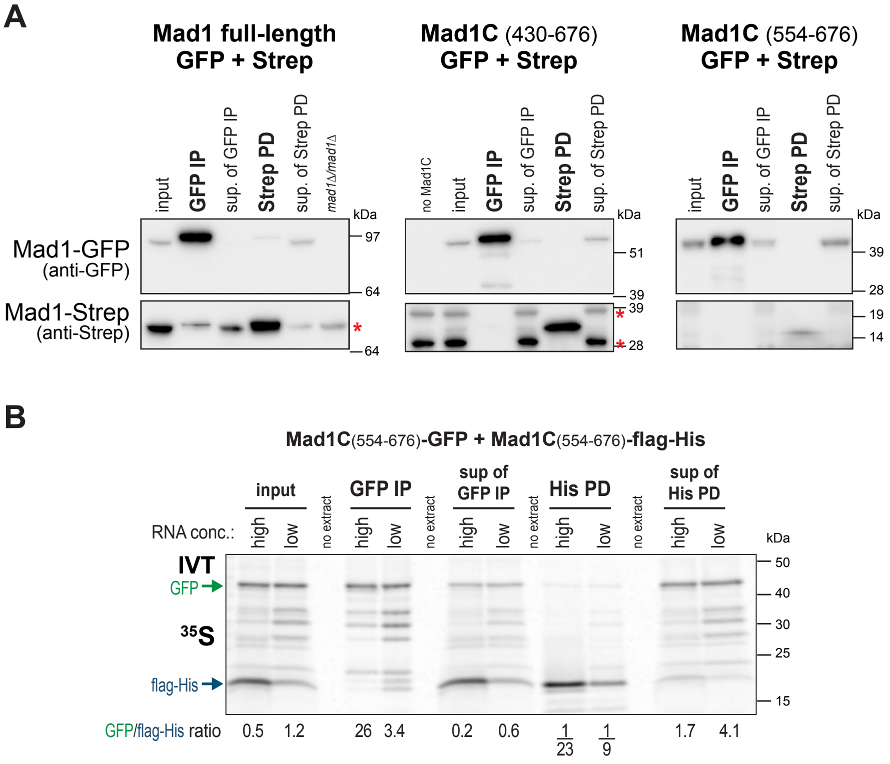

Figure S6. Assessing dimerization of Mad1 C-terminal fragments in yeast cell extract and *in vitro* translation.
(A) Diploid strains, expressing one copy of GFP-tagged and one copy of Strep-tagged Mad1. Full-length Mad1 (left panel) is expressed from the endogenous locus. The C-terminal fragments (middle and right panel) are expressed under the *mad3*$^{+}$ promoter from the exogenous *leu1* locus; in this case, the endogenous copy of *mad1*$^{+}$ was deleted. GFP immunoprecipitation (IP) or Strep pull-down (PD) from the same extract are shown, probed with anti-GFP and anti-Strep; input is 3 % of extract used for IP/PD, sup = supernatant. Red asterisks indicate prominent cross-reactions of the anti-Strep antibody, one of which overlaps with full-length Mad1-Strep. **(B)** Mad1 C-terminal fragments tagged with either GFP or flag-His were *in vitro* transcribed, mixed, and translated in the presence of ^{35}S-labelled Methionine and Cysteine. The in vitro translation was split and subjected to GFP immunoprecipitation (IP) or His pull-down (PD). Input (8 % of extract used for IP/PD) and supernatant (sup) samples after IP/PD are loaded as well. Shown is the autoradiograph after SDS-PAGE. The higher RNA conc. is 40 ng/µL *mad1*C-GFP and 30 ng/µL *mad1*C-flag-His, the lower RNA conc. is 1.14 ng/µL *mad1*C-GFP and 0.86 ng/µL *mad1*C-flag-His. Radioactive bands corresponding to Mad1C-GFP and Mad1-flag-His were quantified and the ratio is given under each lane. For the His PD, the ratio is given as a fraction. One out of two experiments with similar results.

Figure S7

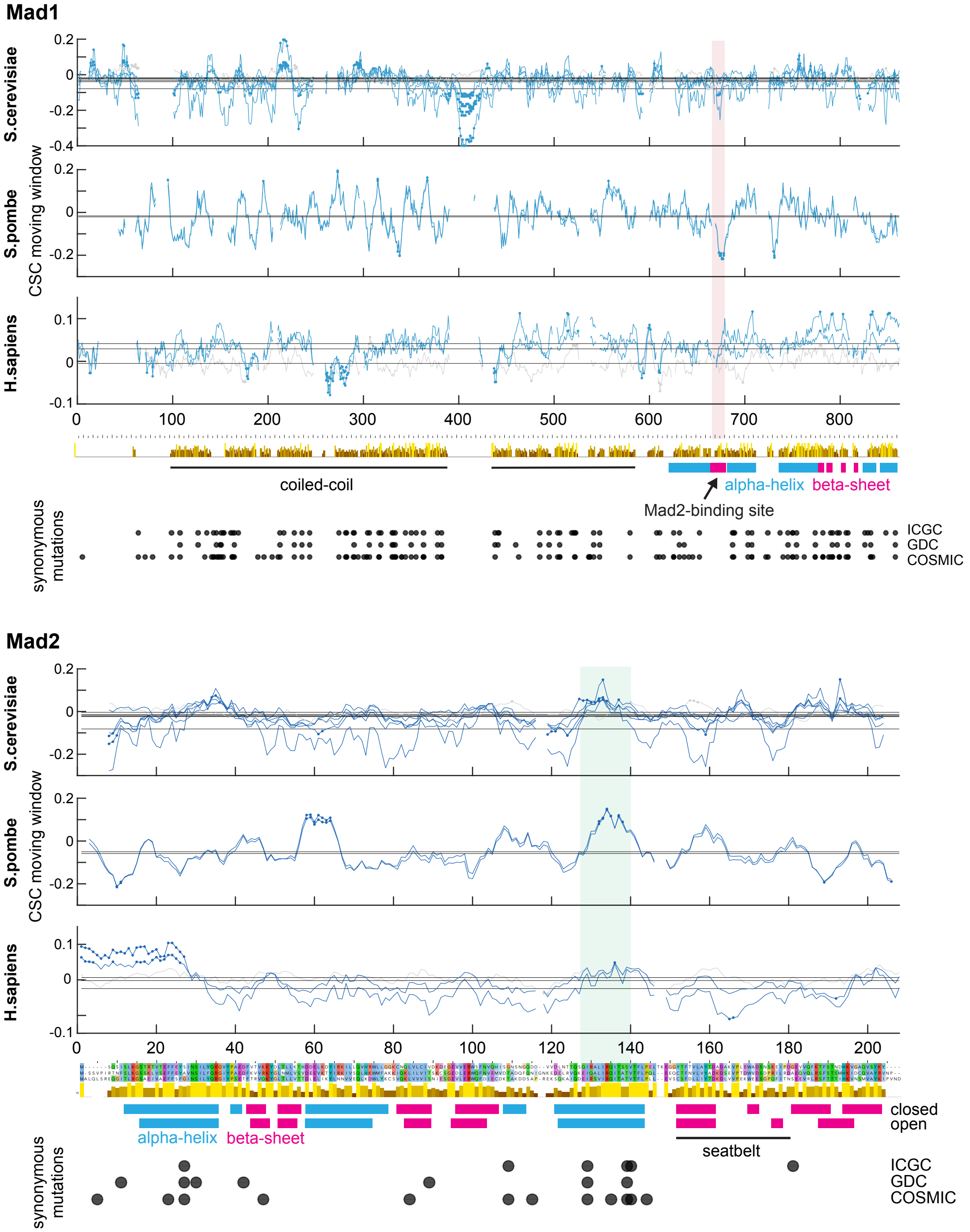

Figure S7. CSC along Mad1 and Mad2 in *S. cerevisiae*, *S. pombe*, and *H. sapiens* and synonymous mutations observed in cancer.

Mad1 and Mad2 were aligned using MAFFT. Yellow/brown bars beneath each panel indicate conservation. Blue and light gray lines show the moving CSC average across 9 codons. CSC values were derived from different mRNA half-life datasets, as explained in the Methods section under 'Codon usage bias calculations', resulting in the multiple lines for each species. Datasets that were based on less data, or showed a different tendency from others are shown in light gray (see Methods for details). Filled circles in the curves indicate positions where the value deviates by more than two standard deviations from the mean for this particular dataset and gene. The thin horizontal lines indicate the mean of CSC values across the gene for each dataset. Secondary structure elements come from solved structures, whose protein data bank (PDB) identifiers are given in brackets. Red-shaded region: Mad2-binding site in Mad1 with markedly low CSC values in *S. pombe* and in one of the *S. cerevisiae* datasets. Green-shaded region: Conserved region in Mad2 with commonly high CSC values in these three organisms. Grey dots at the bottom indicate the position of synonymous mutations found in human cancer databases. In the ICGC and COSMIC data, synonymous mutations are enriched in the Mad2 region marked in green ($p < 0.005$ by chi-square test).

Table S1 - Yeast strains

Strain Number	Mating type	Genotype
**Figure 1B, 2A; S1, S2A (protein concentrations and noise by live-cell imaging)**		
SW184	*h-*	*leu1 Z<<natR<<Padh31-tetR-tdTomato*
SW178	*h+*	*mad1+-ymEGFP Z<<natR<<Padh31-tetR-tdTomato*
SW180	*h+*	*mad2+-ymEGFP Z<<natR<<Padh31-tetR-tdTomato*
SW182	*h+*	*mad3+-ymEGFP Z<<natR<<Padh31-tetR-tdTomato*
SW200	*h?*	*nmt1+-ymEGFP<<kanMX6 Z<<natR<<Padh31-tetR-tdTomato*
**Figure 1C,D; EV1C (WT mRNA numbers by smFISH)**		
SW206	*h+*	*mad1+-ymEGFP*
SW642	*h+*	*mad1+-ymEGFP*
SW130	*h+*	*mad2+-ymEGFP*
SW132	*h+*	*mad3+-ymEGFP*
SW132'	*h+*	*mad3+-ymEGFP*
**Figure 1E; EV1E (mRNA numbers by smFISH, tagged vs. untagged)**		
SW642	*h+*	*mad1+-ymEGFP*
SW130	*h+*	*mad2+-ymEGFP*
JY001	*h-*	wild type
**Figure 1F; EV1D (test of mRNA co-localization and comparison of endogenous and GFP FISH probes)**		
SW642	*h+*	*mad1+-ymEGFP*
SW130	*h+*	*mad2+-ymEGFP*
**Figure 2D (mRNA half-life)**		
JY743	*h-*	*leu1 ura4-D18*
ST932		*S. cerevisiae MET17pr-Fluc::lys2Δ (AMV54, Buchler lab)*
**Figure 2E; S2E (protein half-life)**		
JY001	*h-*	wild type
SU228	*h+*	*bub1+-ymEGFP*
SL221	*h-*	*leu1 ade6-M216 cut1+-GFP<<kanR*
**Figure 3D; EV2B, EV3 (mad2 and mad3 mRNA numbers by smFISH after codon-optimization and/or ste13 deletion)**		
SW130	*h+*	*mad2+-ymEGFP*
SW131	*h+*	*mad2-codonOpt-ymEGFP*
SW132	*h+*	*mad3+-ymEGFP*
SW132'	*h+*	*mad3+-ymEGFP*
SW133	*h+*	*mad3-codonOpt-ymEGFP*
SW153	*h+*	*mad2+-ymEGFP ste13Δ::kanMX6*
SW153'	*h+*	*mad2+-ymEGFP ste13Δ::kanMX6*
SW154	*h+*	*mad3+-ymEGFP ste13Δ::kanMX6*
SW154'	*h+*	*mad3+-ymEGFP ste13Δ::kanMX6*
SW157	*h+*	*mad2+-ymEGFP ste13Δ::kanMX6*
SW158	*h+*	*mad3+-ymEGFP ste13Δ::kanMX6*
SW193	*h+*	*mad2-codonOpt-ymEGFP ste13Δ::kanMX6*
SW193'	*h+*	*mad2-codonOpt-ymEGFP ste13Δ::kanMX6*
SW194	*h+*	*mad3-codonOpt-ymEGFP ste13Δ::kanMX6*
SW194'	*h+*	*mad3-codonOpt-ymEGFP ste13Δ::kanMX6*
**Figure 3E, 5D; EV2C (mad2 and mad3 mRNA half-life after ste13 deletion)**		
JY743	*h-*	*leu1 ura4-D18*
ST932		*S. cerevisiae MET17pr-Fluc::lys2Δ (AMV54, Buchler lab)*
SW239	*h-*	*leu1 ura4-D18 ste13Δ::kanMX6*
**Figure 4A,C (Mad2 and Mad3 protein levels after codon-optimization by immunoblotting)**		
SU257	*h+*	*leu1+<< Park1-mCherry cut11+-mCherry<<hph mad2+-ymEGFP*
SU263	*h+*	*leu1+<< Park1-mCherry cut11+-mCherry<<hph mad2-codonOpt-ymEGFP*
SU131	*h-*	*leu1 ura4-D18 mad2+-ymEGFP*
SU128	*h-*	*leu1 ura4-D18 mad2-codonOpt-ymEGFP*
SU294	*h+*	*leu1+<< Park1-mCherry cut11+-mCherry<<hph mad3+-ymEGFP*
SW120	*h+*	*leu1+<< Park1-mCherry cut11+-mCherry<<hph mad3-codonOpt-ymEGFP*
SW121	*h+*	*leu1+<< Park1-mCherry cut11+-mCherry<<hph mad3-codonOpt-ymEGFP*
**Figure 4B,C (Mad2 and Mad3 protein levels after ste13 deletion by immunoblotting)**		
JY001	*h-*	wild type
JY002	*h+*	wild type
SW148	*h+*	*ste13Δ::kanMX6*
**Figure 4D,E; S1, S2A (Mad2 and Mad3 protein levels after codon-optimization by live-cell imaging)**		
SW184	*h-*	*leu1 Z<<natR<<Padh31-tetR-tdTomato*
SW180	*h+*	*mad2+-ymEGFP Z<<natR<<Padh31-tetR-tdTomato*
SW181	*h+*	*mad2-codonOpt-ymEGFP Z<<natR<<Padh31-tetR-tdTomato*
SW182	*h+*	*mad3+-ymEGFP Z<<natR<<Padh31-tetR-tdTomato*
SW183	*h+*	*mad3-codonOpt-ymEGFP Z<<natR<<Padh31-tetR-tdTomato*
**Figure 5B; EV4A,B, EV5A (mad1 mRNA numbers by smFISH after codon-optimization and/or ste13 deletion)**		
SW206	*h+*	*mad1+-ymEGFP*
SW642	*h+*	*mad1+-ymEGFP*
SW129	*h+*	*mad1+-codonOpt-ymEGFP*
SW152	*h+*	*mad1+-ymEGFP ste13Δ::kanMX6*
SW156	*h+*	*mad1+-ymEGFP ste13Δ::kanMX6*
SW192	*h+*	*mad1-codonOpt-ymEGFP ste13Δ::kanMX6*
SW248	*h+*	*mad1-codonOpt-ymEGFP ste13Δ::kanMX6*
SW618	*h+*	*mad1+-codonOpt2-ymEGFP*
SW618'	*h+*	*mad1+-codonOpt2-ymEGFP*
**Figure 5C,D; EV4D,E (mad1, ecm33, and act1 mRNA half-life after ste13 deletion)**		
JY743	*h-*	*leu1 ura4-D18*
ST932		*S. cerevisiae MET17pr-Fluc::lys2Δ (AMV54, Buchler lab)*
SW239	*h-*	*leu1 ura4-D18 ste13Δ::kanMX6*
**Figure 6A,C (Mad1 protein levels after codon-optimization by immunoblotting)**		
SW106	*h+*	*leu1+<<Park1-mCherry cut11+-mCherry<<hph mad1+-ymEGFP*
SU282	*h+*	*leu1+<<Park1-mCherry cut11+-mCherry<<hph mad1-codonOpt-ymEGFP*
SU499	*h-*	*leu1 ura4-D18 mad1+-ymEGFP*

SU136	*h-*	*ura4-D18 mad1+-codonOpt-ymEGFP*
**Figure 6B,C (Mad1 protein levels after ste13 deletion by immunoblotting)**		
JY001	*h-*	wild type
JY002	*h+*	wild type
SW148	*h+*	*ste13Δ::kanMX6*
**Figure 6D,E; S1, S2A (Mad1 protein levels after codon-optimization by live-cell imaging)**		
SW184	*h-*	*leu1 Z<<natR<<Padh31-tetR-tdTomato*
SW178	*h+*	*mad1+-ymEGFP Z<<natR<<Padh31-tetR-tdTomato*
SW179	*h+*	*mad1-codonOpt-ymEGFP Z<<natR<<Padh31-tetR-tdTomato*
**Figure 6F; S4F (Spindle assembly checkpoint-mediated mitotic delay in cells expressing wild-type or codon-optimized Mad1)**		
SX073	*h?*	*alp7Δ::hygR plo1+-tdTomato<<kanR mad1+-ymEGFP*
SX075	*h?*	*alp7Δ::hygR plo1+-tdTomato<<kanR mad1-codonOpt-ymEGFP*
SX074	*h?*	*alp7Δ::hygR plo1+-tdTomato<<kanR mad1+-ymEGFP*
SX076	*h?*	*alp7Δ::hygR plo1+-tdTomato<<kanR mad1-codonOpt-ymEGFP*
**Figure 7A (Test for Mad1 co-translational assembly in haploid strain with tagged and untagged copies of mad1)**		
SU164	*h+*	*leu1<<110nt-mad1+-ymEGFP-164nt*
**Figure 7B; EV6B (Test for Mad1 co-translational assembly in diploid strain with two differently tagged copies of mad1)**		
SW623	*h+/h-*	*leu1/leu1 ade6-M210/ade6-M216 ura4+/ura4-D18 mad1+-TEV-Strep2/mad1+-ymEGFP*
**Figure 7E; EV6D (mad1 mRNA dimerization experiment)**		
JY265	h-	*leu1*
SX411	h-	*leu1<<110nt-mad1+-ymEGFP-164nt*
SX413	h-	*leu1<<110nt-mad1+-ymEGFP-164nt mad1Δ::ura4+*
SW176	h+	*mad1+-ymEGFP mad3+-ymEGFP*
**Figure 7F (mad1 mRNA dimerization experiment)**		
SW176	h+	*mad1+-ymEGFP mad3+-ymEGFP*
**Figure EV1A (Immunoblot of strains traditionally tagged or tagged by scar-free genome editing)**		
SW206	*h+*	*mad1+-ymEGFP*
SK578	*h+*	*leu1 mad1+-GFP<<kanR cut11+-mCherry<<hph*
SW130	*h+*	*mad2+-ymEGFP*
SK580	*h+*	*mad2+-GFP<<kanR cut11+-mCherry<<hph*
SW132	*h+*	*mad3+-ymEGFP*
SK581	*h+*	*mad3+-GFP<<kanR cut11+-mCherry<<hph*
**Figure EV1B (Growth assay of strains with tagged mad1, mad2 and mad3, and ste13 deletion strain)**		
SW206	*h+*	*mad1+-ymEGFP*
SW129	*h+*	*mad1-codonOpt-ymEGFP*
SW148	*h+*	*ste13Δ::kanMX6*
JY001	*h-*	wild type
PV273	*h+*	*leu1 ade6-M216 nda3-KM311*
ST042'	*h-*	*leu1 ura4-D18 mad1Δ::ura4+*
SW130	*h+*	*mad2+-ymEGFP*
SW131	*h+*	*mad2-codonOpt-ymEGFP*
SW132	*h+*	*mad3+-ymEGFP*
SW133	*h+*	*mad3-codonOpt-ymEGFP*
**Figure EV2A (Growth assay of ste13 deletion strain)**		
JY001	*h-*	wild type
SW147	*h-*	*ste13Δ::kanMX6*
**Figure EV4C, EV5B (Untagged mad1 mRNA numbers by smFISH with or without ste13 deletion)**		
SW130	*h+*	*mad2+-ymEGFP*
SW193'	*h+*	*mad2-codonOpt-ymEGFP ste13Δ::kanMX6*
SW194'	*h+*	*mad3-codonOpt-ymEGFP ste13Δ::kanMX6*
SW153'	*h+*	*mad2+-ymEGFP ste13Δ::kanMX6*
**Figure S4A,B (Mad1 protein half-life after codon-optimization)**		
SW199	*h?*	*mad1-codonOpt-ymEGFP mad2-codonOpt-ymEGFP mad3-codonOpt-ymEGFP*
SW234	*h+*	*mad1+-ymEGFP mad2+-ymEGFP mad3+-ymEGFP*
**Figure S4C (Test for Mad2-association of codon-optimized Mad1)**		
SW206	*h+*	*mad1+-ymEGFP*
SW129'	*h+*	*mad1-codonOpt-ymEGFP*
**Figure S4D,E (Test for kinetochore association and checkpoint function of codon-optimized Mad1)**		
SW603	*h-*	*mad1+-ymEGFP plo1+-mCherry<<natR nda3-KM311*
SW605	*h-*	*mad1Δ::ymEGFP plo1+-mCherry<<natR nda3-KM311*
SW601	*h+*	*mad1-codonOpt-ymEGFP plo1+-mCherry<<natR nda3-KM311*
**Figure S5A,B (Replacement of mad1 coding sequence with GFP or GFP fusions)**		
JY001	*h-*	wild type
SW642	*h+*	*mad1+-ymEGFP*
SW206	*h+*	*mad1+-ymEGFP*
SW201	*h-*	*mad1Δ::ymEGFP*
SW203	*h-*	*mad1Δ::ymEGFP*
SW207	*h+*	*mad1Δ::mad1(66bp)-ymEGFP*
SW221	*h+*	*mad1Δ::mad1(108bp)-ymEGFP*
SU200	*h?*	*mad1Δ::nmt1+-ymEGFP*
**Figure S5C,D (Replacement of mad2 and mad3 coding sequences with GFP)**		
SW130'	*h+*	*mad2+-ymEGFP*
SW139	*h+*	*mad2Δ::ymEGFP*
SW140'	*h+*	*mad2Δ::ymEGFP*
SW132	*h+*	*mad3+-ymEGFP*
SU186	*h+*	*mad3Δ::ymEGFP*
SU188	*h+*	*mad3Δ::ymEGFP*
**Figure S6A (Interaction of Mad1 C-terminal fragments)**		
SW623	*h+/h-*	*leu1/leu1 ade6-M210/ade6-M216 ura4+/ura4-D18 mad1+-TEV-Strep2/mad1+-ymEGFP*
		leu1+<<Pmad3-mad1-430-676-ymEGFP/leu1+<<Pmad3-mad1-430-676-Strep ade6-M216/ade6-M210
SX058	*h+/h-*	*mad1Δ::ura4+/mad1Δ::ura4+*
		leu1+<<Pmad3-mad1-554-676-ymEGFP/leu1+<<Pmad3-mad1-554-676-Strep ade6-M216/ade6-M210
SX060	*h+/h-*	*mad1Δ::ura4+/mad1Δ::ura4+*

Table S2 - sgRNA targeting sequences

gene	targeting.sequence
mad1	GCGCTCTCCGAGATTAGCAT
mad2	ATTGGGTAGACAGTGACCCT
mad2	GTTCAGGTGAAGATTTAGAG
mad3	GCAATTTACTCACCGTTGGT
leu1 intergenic	GTAAGTACACAGCGACAACT

Table S3 - codon-optimized SAC gene sequences

gene	company	sequence
**mad1-codonOpt**	Genscript	ATGGCGGATTCTCCTAGaGATCCtTTCCAATCACGTTCACAATTACCTAGATTTTTAGCAACATCAGTTAAGAAACCAAAC<br>CTTAAGAAACCTTCTGTTAACTCTGCTAAtGGTATGTGAAACTTATATTTGACGTTTGTTTATGGATCTGACACCCTGTAG<br>AAACTAAAAATCCTAAGCTTGCTTCTCTTGAATTTCAATTGGAAAATCTTAAAAATGATCTTAAGCGTAAGGAACTTGAAT<br>TTGAACGTGAACAAATTGAACTTCAACGTAAATTGGCTGAAGAACATGAACAAAAGAATTCTTTGCAACTTCGTCTTACT<br>TTGGTTGAAAAGCAACTTGAAGAACAATCTACTTCTTATCAAAAGGAAATTGAAGAAGTTCGTAATGAAAAAGAAGCTA<br>CTCAAGTTAAGATTCATGAACTTCTTGATGCTAAGTGGAAGGAAATTGCTGAATTGAAGACTCAAATTGAAAAGAATGA<br>TCAAGCTCTTTCTGAAAAGAATCATGAAGTTATGGTTTCTAACCAAGCTTTGCAAATGAAGGATACTAATCTTACTAATTT<br>GGAAAAGCTTTTTGCTGATTCTCGTGAACAATTGGAAACTAAGTGTAAGGAATTAGCTGCTGCTGAACAACAACTTCAA<br>GAATTGTCTGTTCATAATCAACAACTTGAAGAATCTATTAAACAAGTTTCTTCTTCTATTGAATTGGAAAAGATTAATGCT<br>GAACAACGTCTTCAAATTTCTGAACTTGAAAAGCTTAAAGCTGCTCAAGAAGAACGTATTGAAAAGCTTTCTTCTAACAA<br>CCGTAATGTTGAAATTCTTAAAGAAGAAAAGAATGATCTTGAATCTAAGCTTTACCGTTTTGAAGAATACCGTGATAAGG<br>TTGCTACTCTTGAACTTGAAAACGAAAAGATTCAAACTGAACTTAACTCTTGGAAATCTTTGATTACTAATGAATTACCTA<br>CTCCTGAAGCTGTTTCTAATAAGTTGGTTTTTCTTCAAAACACTAATGCTAATCTTGGTGAACGTGTTTCTTCTTTGGAAT<br>CTCAACTTTCTAATAAGCCTGCTAATCAACCTCTTGGTGCTAATGAAAAAGATGCTGCTCATATTACTGAATTGGAAACT<br>AAGCTTAAGGAATTGCATGAACAAAATCGTCGTTTACAACGTCAAAAATCTCTTGCTACTCAAGAAATTGATTTGCTTCG<br>TGAAAATTTGAAGTCTTACGATGATGAAGAAGCTATTCTTTCTGAAAAGAATACTGATATGAAGAAACTTGAACGTATTG<br>AAGGTTTAGTTAAGCTTGTTGATGAATACAAGTTGAAATTAGAATCTATGCCTGTTTCTCTTGATGTTGATGAAACTTCTG<br>ATGAAGTTTCTTTGCAAAAACGTCGTCGTAAAAATGAACATAAGGATGCTGGTTACGTTACTGAACTTTATCGTAAAAAT<br>CAACATCTTTTGTTTCAAGTTAAAGAAAAGACTAACATTGAAGCTTTTCTTCGAGAACAAATTATTACTTTGGAATCTTCT<br>ATTGCTACTCTTCGTCAAGAATTGGCTCAAGTTACTGAAATTAATTCTTGTCGTGTTCTTCAACATCGTTCTAACCCTACT<br>CTTAAGTACGAACGTATTAAAGCTGCTCAATTGGAAATGCTTAACGCTGAAAACTCTGCTCTTAAGGCTCTTCTTGAAGA<br>TAAGAAAGTTGATTGTCTTCCTATTCAATCTTTTAAAATTGCTGAACGTAAAGCTCTTGATCTTAAGAAAGAAGTTGCTGA<br>ACGTGAAAAACGTATTCAACGTCTTAAGGAAATTTTCTCTGTTAAGTCTTTGGAATTTCGTGAAGCTGTTTTCTCTCTTTTT<br>GGTTACAAGTTGGATTTTATGCCTAACGGTTCTGTTCGTGTTACTTCTACTTACTCTCGTGAAGATAACACTGCTTTTATT<br>TTTGATGGTGAATCTTCTACTATGAAATTGGTTGGTAATCCTTCTGGTCCTGAATTTGAACGTTTAATTCGTTTTTGGTGT<br>GATGAACGTAAAACTATTCCTGGTATGCTTGCTGCTCTTACTCTTGAACTTCTTGATAAAAATGAT
**mad1-codonOpt2**	IDT	ATGGCAGACTCTCCTCGCGACCCCTTTCAATCCAGATCACAATTACCCAGATTCCTTGCAACTAGTGTTAAGAAACCTAA<br>TCTTAAAAAACCCTCAGTTAATTCTGCTAATGGTATGTGAAACTTATATTTGACGTTTGTTTATGGATCTGACACCCTGTA<br>GAAACAAAAAATCCTAAATTAGCATCACTTGAGTTTCAGTTGGAAAACTTAAAAAATGATTTGAAGCGCAAGGAACTTG<br>AGTTTGAACGTGAGCAGATTGAGTTGCAACGCAAATTGGCAGAAGAACACGAGCAAAAAAATTCATTGCAGTTAAGAT<br>TGACCTTAGTGGAAAAGCAATTGGAAGAGCAATCAACATCTTATCAAAAGGAGATCGAGGAAGTGAGAAACGAGAAA<br>GAAGCTACTCAAGTAAAGATTCACGAGTTACTTGATGCCAAATGGAAAGAAATTGCTGAGTTAAAAACCCAAATAGAAA<br>AGAATGATCAGGCACTTTCCGAAAAAAATCACGAGGTAATGGTATCCAATCAAGCCTTGCAGATGAAGGATACGAATCT<br>TACAAACTTAGAAAAGTTATTTGCTGATTCTCGCGAGCAGTTAGAGACCAAATGTAAAGAGTTAGCTGCTGCCGAGCAA<br>CAATTACAGGAATTATCTGTACATAACCAACAATTGGAAGAGTCTATCAAACAGGTATCTTCTTCCATCGAATTGGAGAA<br>AATTAATGCTGAGCAACGATTACAGATCTCAGAGTTAGAGAAACTTAAAGCAGCACAAGAAGAGCGAATTGAGAAGCT<br>TTCATCAAACAATCGCAACGTAGAGATTTTGAAGGAGGAGAAAAACGATTTAGAGAGTAAGTTGTACCGATTTGAAGA<br>GTATCGAGATAAGGTAGCTACTTTGGAATTGGAGAACGAGAAAATCCAGACAGAGTTGAATAGTTGGAAAAGTCTTAT<br>AACTAACGAGTTGCCCACTCCCGAAGCTGTCAGTAATAAGCTTGTATTCTTGCAGAATACCAACGCAAATTTAGGAGAG<br>CGAGTATCCTCTTTAGAGTCACAGTTGTCAAATAAGCCTGCCAACCAACCCTTGGGTGCCAATGAAAAGGACGCTGCCC<br>ACATCACCGAGTTGGAAACAAAGCTTAAGGAATTACACGAGCAAAACAGACGTCTTCAACGACAAAAGAGTTTGGCTA<br>CTCAGGAGATTGACTTGTTGAGAGAAAATTTAAAATCATATGATGATGAAGAGGCCATACTTTCAGAGAAAAATACCGA<br>TATGAAAAAATTGGAACGCATCGAAGGCTTAGTAAAACTTGTAGATGAGTACAAATTGAAGCTTGAATCAATGCCCGTC<br>TCTCTTGACGTGGATGAAACTAGTGACGAAGTGTCATTACAAAAGCGAAGACGTAAAAACGAACATAAAGACGCTGGC<br>TATGTGACGGAGTTGTACCGCAAAAACCAACACTTGTTGTTTCAGGTGAAAGAGAAGACAAATATAGAAGCCTTCTTAC<br>GAGAACAAATCATTACGTTGGAATCCTCAATCGCTACATTAAGACAGGAGCTTGCTCAGGTAACGGAGATAAACAGTTG<br>TCGCGTCCTTCAGCATCGTTCCAATCCCACTTTGAAGTACGAAAGAATAAAGGCTGCACAACTTGAGATGTTGAACGCA<br>GAAAACAGTGCTCTTAAGGCCTTGTTGGAGGATAAGAAGGTGGACTGTCTTCCCATCCAGAGTTTTAAGATAGCTGAAA<br>GAAAAGCATTGGATTTGAAAAAGGAAGTTGCAGAGAGAGAAAAACGCATTCAGCGTTTAAAGGAGATCTTTTCAGTCA<br>AATCCTTAGAGTTCCGTGAGGCTGTCTTTTCATTATTTGGATATAAGTTGGACTTCATGCCTAACGGAAGTGTGCGTGTA<br>ACTTCCACGTATTCTCGAGAGGACAATACCGCTTTTATATTCGATGGAGAGTCATCAACCATGAAGTTGGTGGGTAACC<br>CATCTGGACCAGAATTCGAGCGATTGATACGATTCTGGTGTGATGAGAGAAAAACCATTCCTGGCATGTTAGCCGCTTT<br>GACCCTTGAGTTATTAGATAAAAATGAC
**mad2-codonOpt**	Genscript	ATGTCTTCTGTTCCTATTCGTACTAACTTTTCTCTTAAGGGTTCTTCTAAACTTGTTTCTGAATTTTTTGGTTTGTAAATGTT<br>TCAAATATGCCATGCTAACTTAATGAAGAATACGCTGTTAACTCTATTTTGTTTCAACGTGGTATCTATCCTGCTGAAGAT<br>TTTAAGGTTGTTCGTAAGTACGGTTTAAACATGCTTGTTTCTGTTGATGAAGAAGTTAAAACTTATATTCGTAAAATTGTT<br>TCTCAATTACACAGTGAGTTGTTGTTCTATTATCACTTTTGGAATTAACAAATTTTTAGAATGGATGTTTGCTAAGAAAATT<br>CAAAAGCTTATTCTTGTTATTACTTCTAAGTGTTCTGGTGAAGATTTGGAACGTTGGCAATTCAATGTTGAAATGGTTGAT<br>ACTGCTGATCAATTTCAAAACATTGGTAATAAGGAAGATGAACTTCGTGTTCAAAAGGAAATTCAAGCTCTTATTCGTCA<br>AATTACTGCTACTGTTACTTTTCTTCCTCAACTTGAAGAACAATGTACTTTTAATGTTTTAGTTTATGCTGATAAAGATTCT<br>GAAGTTCCTACTGATTGGGTTGATTCTGATCCTCGTATTCTTCGTGATGCTGAACAAGTTCAATTGCGTTCTTTTTCTACT<br>TCTATGCATAAGATTGATTGTCAAGTTGCTTATCGTGTTAATCCT
**mad3-codonOpt**	IDT	ATGGAACCACTTGATGCAGGTAAAAACTGGGTTCATATGGATGTTATTGAACAATCTAAGGAAAACATTGAACCACGAA<br>AGGCAGGTCATTCTGCTTCTGCTTTGGCTAAGTCTTCTTCTCGTAACCATACTGAAAAAGAAGTTGCTGGTTTACAAAAA<br>GAACGTATGGGTCATGAGCGTAAGATTGAAACTTCTGAATCTCTTGATGATCCTTTGCAAGTTTGGATTGATTACATTAA<br>GTGGACTCTTGATAACTTTCCTCAAGGTGAAACTAAGACTTCTGGTTTGGTTACTTTACTTGAACGTTGTACTCGTGAATT<br>TGTTCGTAACCCTTTGTACAAGGATGATGTTCGTTATCTTCGTATTTGGATGCAATACGTTAACTACATTGATGAACCTGT<br>TGAATTATTTTCTTTTCTTGCTCATCATCATATTGGTCAAGAATCTTCTATTTTCTATGAAGAATACGCTAACTACTTTGAAT<br>CTCGTGGTTTATTTCAAAAGGCTGATGAAGTTTACCAAAAGGGTAAACGTATGAAGGCTAAGCCTTTTCTTCGTTTTCAA<br>CAAAAGTACCAACAATTCACTCATCGTTGGCTTGAATTTGCTCCTCAATCTTTTTCTTCTAACACTAACTCTGTTAACCCTC<br>TTCAAACTACTTTTGAATCTACTAACATTCAAGAAATTTCTCAATCTCGTACTAAGATTTCTAAGCCTAAGTTTAAATTTTC<br>TGTTTATTCTGATGCTGATGGTTCTGGTAAAGATGGTCAACCTGGTACTTGGCAAACTTTGGGTACTGTTGATCAACGTC<br>GTAAGGAAAACAACATTTCTGCTACTTCTTGGGTTGGTGAAAAATTGCCTTTAAAATCTCCTCGTAAGTTAGATCCATTG<br>GGAAAGTTTCAAGTTCATTGCGATGAAGAAGTTTCAAAGGAA

Table S4 - FISH probes

mad2	ymEGFP	mad1
cgttcttatgggaacgctag	cagtgaataattcttcacctt	gaacggatccctaggagaatc
gtttggaagaacctttcagt	tcaaccaaaattgggacaaca	aatctaggcaactgtgaacgc
aaaggattgagttcaccgca	gaccattaacatcaccatcta	atttggtttcttaacgcttgt
gtcttctgctgggtaaattc	ccttcaccggagacagaaaat	gcagaattaacagaaggcttc
tccatatttccgaacaactt	gtcaatttaccgtaagtagca	agctagtttgggatttttttgt
tcatctacactgacaagcat	atggaactggcaatttaccag	ccttccgctttaaatcatttt
tcgaatgtaagtcttgacct	aaagtagtgactaaggttggc	actcaatttgttcacgctcaa
ttgcaaacatccatttgtgt	tgttgtttcatatgatctggg	ttcttctgcaagttttctttg
cgctctaaatcttcacctga	ctggcatggcagacttgaaaa	gctgtaacgaattcttctgtt
catctccacattaaactgcc	gttctttcttgaacataacct	agttgcttttcaactagagtt
gaaattgatcagctgtgtca	agttaccgtcatctttgaaaa	gataagaagtagactgctcct
atcttctttgttgccaatgt	ttgacttcagctctggtcttg	ccttttcatttcttacttctt
tttctttttgtactcgcagt	taactaaggtatcaccttcaa	ttgcatcaagtagttcatgga
tgtagcagtgatttgacgaa	atacctttttaattcgattcta	ttcaactctgcaatctctttc
ctagttgaggcaaaaaggtc	cctaaaatgttaccatcttct	gatcattcttttctatctggg
acgttaaacgtacactgttc	gagagttatagttgtattcca	acttcatgattcttttcactt
gtctttatcagcgtatacca	gtcagccatgatgtaaacatt	tgcaaagcttgatttgagacc
cccaatctgttggaacttcg	actttgataccattcttttgt	gtttgtaagattggtatcctt
aaaatcctagggtcactgtc	gttgtgtctaattttgaagtt	ggaatccgcaaagagtttttc
ttgaacttgttcagcatctc	attgaacagaaccatcttcaa	ttccttacacttcgtttcaag
attttgtgcatactcgtact	ttttgttgataatggtcagct	gaaagctcttgtaattgctgc
ggattcactcgatatgcaac	agactggaccatcaccaattg	tcttccaattgctgattatga
	aagtaatggttgtctggtaac	aactagaaacctgcttgatgg
	ggataaacgagattgagtgga	gcatttattttttccagttca
	ctctcttttcgtttggatctt	cgctaatttgaagacgttgct
	aattctaacaagaccatgtgg	cgcagcttttaatttttccaa
	ggtaataccagcagcagtaac	gatagcttttcaattcgttct
	ttgtacaattcatccatacca	ccttgagaatttcaacattcc
		ttggactccaaatcgtttttc
		aaccttatccctatattcttc
		tcattttcgagttcaagggta
		gggttggtaactcgttagtaa
		acgagtttgtttgaaacagct
		ttagcattggtattctgtaga
		taaactagaaacgcgctctcc

Table S5 - qPCR primers

Target	Forward	Reverse	
act1	CCAAATCCAACCGTGAGAAGA	GTACGACCAGAGGCATACAAAG	
cdc2	GGTATCGTGCTCCTGAAGTATTG	CAGAGTCACCGGGAAATAATGG	
ecm33	ATCATTCGCTCTCACTCTTCTTT	GTACCTTGGGCGGAGATATTG	
mad1	CCTAATGGGAGTGTTCGTGTTA	CCTGATGGATTACCAACCAATTTC	
mad2	TTAGAGCGGTGGCAGTTTAAT	CTCGCAGTTCATCTTCTTTGTTG	
mad3	CGGATGGTTCTGGAAAGGAT	CTACCCAAGAAGTAGCCGATATG	
S.c. ACT1	TGGATTCCGGTGATGGTGTT	TCAAAATGGCGTGAGGTAGAGA	Chan et al. 2018
ymEGFP	TGAAGGTGAAGGTGATGCTAC	CTAAGGTTGGCCATGGAACT	

Table S6 - Mad1 sequences for in vitro transcription and translation

gene	nucleotide sequence
Mad1-flag-His	gaaattaatacgactcactataggGACTACATCCATGGCGGATTCTCCTAGGGATCCGTTCCAATCGCGTTCACAGTTGCCTAGATTTCTAGCAACAAGCGTTAAGAAACCA<br>AATTTGAAGAAGCCTTCTGTTAATTCTGCTAATGAAACAAAAAATCCCAAACTAGCTTCTTTAGAATTTCAACTAGAAAACCTAAAAAATGATTTAAAGCGGAAGGA<br>ACTGGAATTTGAGCGTGAACAAATTGAGTTGCAAAGAAAACTTGCAGAAGAACATGAACAGAAGAATTCGTTACAGCTGCGTCTAACTCTAGTTGAAAAGCAACT<br>GGAGGAGCAGTCTACTTCTTATCAAAAAGAAATAGAAGAAGTAAGAAATGAAAAGGAAGCCACTCAGGTGAAGATCCATGAACTACTTGATGCAAAATGGAAAGAG<br>ATTGCAGAGTTGAAAACCCAGATAGAAAAGAATGATCAAGCTTTAAGTGAAAAGAATCATGAAGTAATGGTCTCAAATCAAGCTTTGCAAATGAAGGATACCAATC<br>TTACAAACCTTGAAAAACTCTTTGCGGATTCCCGCGAACAGCTTGAAACGAAGTGTAAGGAACTTGCGGCTGCCGAGCAGCAATTACAAGAGCTTTCAGTTCAT<br>AATCAGCAATTGGAAGAGTCCATCAAGCAGGTTTCTAGTTCTATTGAACTGGAAAAAATAAATGCTGAGCAACGTCTTCAAATTAGCGAGTTGGAAAAATTAAAAG<br>CTGCGCAGGAAGAACGAATTGAAAAGCTATCATCCAATAATAGGAATGTTGAAATTCTCAAGGAAGAGAAAAACGATTTGGAGTCCAAACTTTATCGTTTTGAAGA<br>ATATAGGGATAAGGTTGCTACCCTTGAACTCGAAAATGAAAAAATTCAAACTGAATTAAATTCTTGGAAGAGTTTAATTACTAACGAGTTACCAACCCCTGAAGCT<br>GTTTCAAACAAACTCGTGTTTCTACAGAATACCAATGCTAATCTCGGAGAGCGCGTTTCTAGTTTAGAGAGTCAATTGTCTAATAAGCCTGCAAACCAGCCGCTT<br>GGTGCAAATGAAAAAGACGCCGCTCATATTACCGAACTTGAAACAAAGCTTAAAGAACTTCATGAGCAGAACCGACGTCTACAAAGACAAAAATCTTTGGCAACC<br>CAAGAAATTGACCTTTTAAGGGAAAACCTTAAAAGCTATGATGATGAGGAGGCGATTTTGAGTGAAAAAAATACTGATATGAAGAAGTTGGAAAGAATTGAAGGTC<br>TGGTCAAGCTGGTTGATGAATATAAGCTAAAGCTTGAAAGTATGCCTGTATCTCTTGATGTAGATGAGACTTCAGATGAGGTCTCATTACAAAAGAGACGCAGGA<br>AAAACGAACATAAAGATGCTGGTTACGTAACGGAGCTATATCGTAAGAATCAGCATTTGCTTTTTCAAGTTAAAGAGAAAACCAACATTGAAGCCTTTCTAAGGGA<br>GCAGATTATTACATTGGAATCCTCCATAGCAACTTTACGTCAAGAACTGGCACAAGTTACTGAAATAAACTCATGTCGAGTATTACAGCATCGATCCAATCCTAC<br>TTTGAAATATGAGAGGATTAAAGCTGCGCAATTGGAGATGCTGAATGCTGAGAACTCAGCTTTGAAAGCTCTTTTGGAAGATAAAAAGGTTGATTGTCTACCTATA<br>CAATCATTTAAAATAGCTGAAAGAAAGGCCTTGGATTTAAAGAAAGAAGTAGCCGAGAGAGAAAAGAGAATTCAACGGTTAAAGGAGATTTTTTCAGTAAAGTCTTT<br>GGAATTTCGCGAAGCTGTTTTTTCGCTTTTTGGATATAAGCTCGATTTTATGCCTAATGGGAGTGTTCGTGTTACATCCACATATTCTCGTGAAGATAATACCGC<br>TTTTATATTTGATGGCGAATCCAGCACCATGAAATTGGTTGGTAATCCATCAGGCCCCGAATTTGAGCGCCTTATACGATTTTGGTGTGATGAACGCAAAACAAT<br>ACCAGGCATGTTAGCGGCTCTAACGTTAGAGCTTCTTGACAAAAATGATggctctGATTATAAGGATGATGACGATAAACCCtccGACTACAAGGACGACGATGATAA<br>ACATCACCACCATCACCACggaTGATTAACTTACGCCTTAAGCATCATCTTACAGGCGATGAATATGTACACTAACAAATACGCATAATCAAAATGTGAAAAGTAAT<br>ACTTTTTTATGAAATAGATTGTTTAACTAATAATATCAATATATCCTTC
Mad1 untagged	gaaattaatacgactcactataggGACTACATCCATGGCGGATTCTCCTAGGGATCCGTTCCAATCGCGTTCACAGTTGCCTAGATTTCTAGCAACAAGCGTTAAGAAACCA<br>AATTTGAAGAAGCCTTCTGTTAATTCTGCTAATGAAACAAAAAATCCCAAACTAGCTTCTTTAGAATTTCAACTAGAAAACCTAAAAAATGATTTAAAGCGGAAGGA<br>ACTGGAATTTGAGCGTGAACAAATTGAGTTGCAAAGAAAACTTGCAGAAGAACATGAACAGAAGAATTCGTTACAGCTGCGTCTAACTCTAGTTGAAAAGCAACT<br>GGAGGAGCAGTCTACTTCTTATCAAAAAGAAATAGAAGAAGTAAGAAATGAAAAGGAAGCCACTCAGGTGAAGATCCATGAACTACTTGATGCAAAATGGAAAGAG<br>ATTGCAGAGTTGAAAACCCAGATAGAAAAGAATGATCAAGCTTTAAGTGAAAAGAATCATGAAGTAATGGTCTCAAATCAAGCTTTGCAAATGAAGGATACCAATC<br>TTACAAACCTTGAAAAACTCTTTGCGGATTCCCGCGAACAGCTTGAAACGAAGTGTAAGGAACTTGCGGCTGCCGAGCAGCAATTACAAGAGCTTTCAGTTCAT<br>AATCAGCAATTGGAAGAGTCCATCAAGCAGGTTTCTAGTTCTATTGAACTGGAAAAAATAAATGCTGAGCAACGTCTTCAAATTAGCGAGTTGGAAAAATTAAAAG<br>CTGCGCAGGAAGAACGAATTGAAAAGCTATCATCCAATAATAGGAATGTTGAAATTCTCAAGGAAGAGAAAAACGATTTGGAGTCCAAACTTTATCGTTTTGAAGA<br>ATATAGGGATAAGGTTGCTACCCTTGAACTCGAAAATGAAAAAATTCAAACTGAATTAAATTCTTGGAAGAGTTTAATTACTAACGAGTTACCAACCCCTGAAGCT<br>GTTTCAAACAAACTCGTGTTTCTACAGAATACCAATGCTAATCTCGGAGAGCGCGTTTCTAGTTTAGAGAGTCAATTGTCTAATAAGCCTGCAAACCAGCCGCTT<br>GGTGCAAATGAAAAAGACGCCGCTCATATTACCGAACTTGAAACAAAGCTTAAAGAACTTCATGAGCAGAACCGACGTCTACAAAGACAAAAATCTTTGGCAACC<br>CAAGAAATTGACCTTTTAAGGGAAAACCTTAAAAGCTATGATGATGAGGAGGCGATTTTGAGTGAAAAAAATACTGATATGAAGAAGTTGGAAAGAATTGAAGGTC<br>TGGTCAAGCTGGTTGATGAATATAAGCTAAAGCTTGAAAGTATGCCTGTATCTCTTGATGTAGATGAGACTTCAGATGAGGTCTCATTACAAAAGAGACGCAGGA<br>AAAACGAACATAAAGATGCTGGTTACGTAACGGAGCTATATCGTAAGAATCAGCATTTGCTTTTTCAAGTTAAAGAGAAAACCAACATTGAAGCCTTTCTAAGGGA<br>GCAGATTATTACATTGGAATCCTCCATAGCAACTTTACGTCAAGAACTGGCACAAGTTACTGAAATAAACTCATGTCGAGTATTACAGCATCGATCCAATCCTAC<br>TTTGAAATATGAGAGGATTAAAGCTGCGCAATTGGAGATGCTGAATGCTGAGAACTCAGCTTTGAAAGCTCTTTTGGAAGATAAAAAGGTTGATTGTCTACCTATA<br>CAATCATTTAAAATAGCTGAAAGAAAGGCCTTGGATTTAAAGAAAGAAGTAGCCGAGAGAGAAAAGAGAATTCAACGGTTAAAGGAGATTTTTTCAGTAAAGTCTTT<br>GGAATTTCGCGAAGCTGTTTTTTCGCTTTTTGGATATAAGCTCGATTTTATGCCTAATGGGAGTGTTCGTGTTACATCCACATATTCTCGTGAAGATAATACCGC<br>TTTTATATTTGATGGCGAATCCAGCACCATGAAATTGGTTGGTAATCCATCAGGCCCCGAATTTGAGCGCCTTATACGATTTTGGTGTGATGAACGCAAAACAAT<br>ACCAGGCATGTTAGCGGCTCTAACGTTAGAGCTTCTTGACAAAAATGATTGATTAACTTACGCCTTAAGCATCATCTTACAGGCGATGAATATGTACACTAACAA<br>ATACGCATAATCAAAATGTGAAAAGTAATACTTTTTTATGAAATAGATTGTTTAACTAATAATATCAATATATCCTTC
Mad1-GFP	gaaattaatacgactcactataggGACTACATCCATGGCGGATTCTCCTAGGGATCCGTTCCAATCGCGTTCACAGTTGCCTAGATTTCTAGCAACAAGCGTTAAGAAACCA<br>AATTTGAAGAAGCCTTCTGTTAATTCTGCTAATGAAACAAAAAATCCCAAACTAGCTTCTTTAGAATTTCAACTAGAAAACCTAAAAAATGATTTAAAGCGGAAGGA<br>ACTGGAATTTGAGCGTGAACAAATTGAGTTGCAAAGAAAACTTGCAGAAGAACATGAACAGAAGAATTCGTTACAGCTGCGTCTAACTCTAGTTGAAAAGCAACT<br>GGAGGAGCAGTCTACTTCTTATCAAAAAGAAATAGAAGAAGTAAGAAATGAAAAGGAAGCCACTCAGGTGAAGATCCATGAACTACTTGATGCAAAATGGAAAGAG<br>ATTGCAGAGTTGAAAACCCAGATAGAAAAGAATGATCAAGCTTTAAGTGAAAAGAATCATGAAGTAATGGTCTCAAATCAAGCTTTGCAAATGAAGGATACCAATC<br>TTACAAACCTTGAAAAACTCTTTGCGGATTCCCGCGAACAGCTTGAAACGAAGTGTAAGGAACTTGCGGCTGCCGAGCAGCAATTACAAGAGCTTTCAGTTCAT<br>AATCAGCAATTGGAAGAGTCCATCAAGCAGGTTTCTAGTTCTATTGAACTGGAAAAAATAAATGCTGAGCAACGTCTTCAAATTAGCGAGTTGGAAAAATTAAAAG<br>CTGCGCAGGAAGAACGAATTGAAAAGCTATCATCCAATAATAGGAATGTTGAAATTCTCAAGGAAGAGAAAAACGATTTGGAGTCCAAACTTTATCGTTTTGAAGA<br>ATATAGGGATAAGGTTGCTACCCTTGAACTCGAAAATGAAAAAATTCAAACTGAATTAAATTCTTGGAAGAGTTTAATTACTAACGAGTTACCAACCCCTGAAGCT<br>GTTTCAAACAAACTCGTGTTTCTACAGAATACCAATGCTAATCTCGGAGAGCGCGTTTCTAGTTTAGAGAGTCAATTGTCTAATAAGCCTGCAAACCAGCCGCTT<br>GGTGCAAATGAAAAAGACGCCGCTCATATTACCGAACTTGAAACAAAGCTTAAAGAACTTCATGAGCAGAACCGACGTCTACAAAGACAAAAATCTTTGGCAACC<br>CAAGAAATTGACCTTTTAAGGGAAAACCTTAAAAGCTATGATGATGAGGAGGCGATTTTGAGTGAAAAAAATACTGATATGAAGAAGTTGGAAAGAATTGAAGGTC<br>TGGTCAAGCTGGTTGATGAATATAAGCTAAAGCTTGAAAGTATGCCTGTATCTCTTGATGTAGATGAGACTTCAGATGAGGTCTCATTACAAAAGAGACGCAGGA<br>AAAACGAACATAAAGATGCTGGTTACGTAACGGAGCTATATCGTAAGAATCAGCATTTGCTTTTTCAAGTTAAAGAGAAAACCAACATTGAAGCCTTTCTAAGGGA<br>GCAGATTATTACATTGGAATCCTCCATAGCAACTTTACGTCAAGAACTGGCACAAGTTACTGAAATAAACTCATGTCGAGTATTACAGCATCGATCCAATCCTAC<br>TTTGAAATATGAGAGGATTAAAGCTGCGCAATTGGAGATGCTGAATGCTGAGAACTCAGCTTTGAAAGCTCTTTTGGAAGATAAAAAGGTTGATTGTCTACCTATA<br>CAATCATTTAAAATAGCTGAAAGAAAGGCCTTGGATTTAAAGAAAGAAGTAGCCGAGAGAGAAAAGAGAATTCAACGGTTAAAGGAGATTTTTTCAGTAAAGTCTTT<br>GGAATTTCGCGAAGCTGTTTTTTCGCTTTTTGGATATAAGCTCGATTTTATGCCTAATGGGAGTGTTCGTGTTACATCCACATATTCTCGTGAAGATAATACCGC<br>TTTTATATTTGATGGCGAATCCAGCACCATGAAATTGGTTGGTAATCCATCAGGCCCCGAATTTGAGCGCCTTATACGATTTTGGTGTGATGAACGCAAAACAAT<br>ACCAGGCATGTTAGCGGCTCTAACGTTAGAGCTTCTTGACAAAAATGATGTTtctaaaggtgaagaattattcactggtgttgtcccaattttggttgaattagatggtgatgttaatggtcacaaattttctgtctccgg<br>tgaaggtgaaggtgatgctacttacggtaaattgaccttaaaatttatttgtactactggtaaattgccagttccatggccaaccttagtcactactttaacttatggtgttcaatgtttttctagatacccagatcaTatgaaacaacatgactttttcaagtctgccat<br>gccagaaggttatgttcaagaaagaactatttttttcaaagatgacggtaactacaagaccagagctgaagtcaagtttgaaggtgataccttagttaatagaatcgaattaaaaggtattgattttaaagaagatggtaacattttaggtcacaaattggaatac<br>aactataactctcacaatgtttacatcatggctgacaaacaaaagaatggtatcaaagttaacttcaaaattagacacaacattgaagatggttctgttcaattagctgaccattatcaacaaaatactccaattggtgatggtccagtcttgttaccagacaac<br>cattacttatccactcaatctCGTttatccaaagatccaaacgaaaagagagaccacatggtcttgttagaatttgttactgctgctggtattacccatggtatggatgaattgtacaaataaTTAACTTACGCCTTAAGCATCATCTTA<br>CAGGCGATGAATATGTACACTAACAAATACGCATAATCAAAATGTGAAAAGTAATACTTTTTTATGAAATAGATTGTTTAACTAATAATATCAATATATCCTTC
Mad1C-554-676-GFP	gaaattaatacgactcactataggGACTACATCCATGGCGATACAATCATTTAAAATAGCTGAAAGAAAGGCCTTGGATTTAAAGAAAGAAGTAGCCGAGAGAGAAAAGAGAAT<br>TCAACGGTTAAAGGAGATTTTTTCAGTAAAGTCTTTGGAATTTCGCGAAGCTGTTTTTTCGCTTTTTGGATATAAGCTCGATTTTATGCCTAATGGGAGTGTTCGT<br>GTTACATCCACATATTCTCGTGAAGATAATACCGCTTTTATATTTGATGGCGAATCCAGCACCATGAAATTGGTTGGTAATCCATCAGGCCCCGAATTTGAGCGC<br>CTTATACGATTTTGGTGTGATGAACGCAAAACAATACCAGGCATGTTAGCGGCTCTAACGTTAGAGCTTCTTGACAAAAATGATGTTtctaaaggtgaagaattattcactggtgtt<br>gtcccaattttggttgaattagatggtgatgttaatggtcacaaattttctgtctccggtgaaggtgaaggtgatgctacttacggtaaattgaccttaaaatttatttgtactactggtaaattgccagttccatggccaaccttagtcactactttaacttatggtgttcaa<br>tgtttttctagatacccagatcaTatgaaacaacatgactttttcaagtctgccatgccagaaggttatgttcaagaaagaactatttttttcaaagatgacggtaactacaagaccagagctgaagtcaagtttgaaggtgataccttagttaatagaatcgaatt<br>aaaaggtattgattttaaagaagatggtaacattttaggtcacaaattggaatacaactataactctcacaatgtttacatcatggctgacaaacaaaagaatggtatcaaagttaacttcaaaattagacacaacattgaagatggttctgttcaattagctgac<br>cattatcaacaaaatactccaattggtgatggtccagtcttgttaccagacaaccattacttatccactcaatctCGTttatccaaagatccaaacgaaaagagagaccacatggtcttgttagaatttgttactgctgctggtattacccatggtatggatgaa<br>ttgtacaaataaTTAACTTACGCCTTAAGCATCATCTTACAGGCGATGAATATGTACACTAACAAATACGCATAATCAAAATGTGAAAAGTAATACTTTTTTATGAAATAGA<br>TTGTTTAACTAATAATATCAATATATCCTTC
Mad1C-554-676-flag-Hi	gaaattaatacgactcactataggGACTACATCCATGGCGATACAATCATTTAAAATAGCTGAAAGAAAGGCCTTGGATTTAAAGAAAGAAGTAGCCGAGAGAGAAAAGAGAAT<br>TCAACGGTTAAAGGAGATTTTTTCAGTAAAGTCTTTGGAATTTCGCGAAGCTGTTTTTTCGCTTTTTGGATATAAGCTCGATTTTATGCCTAATGGGAGTGTTCGT<br>GTTACATCCACATATTCTCGTGAAGATAATACCGCTTTTATATTTGATGGCGAATCCAGCACCATGAAATTGGTTGGTAATCCATCAGGCCCCGAATTTGAGCGC<br>CTTATACGATTTTGGTGTGATGAACGCAAAACAATACCAGGCATGTTAGCGGCTCTAACGTTAGAGCTTCTTGACAAAAATGATggctctGATTATAAGGATGATGAC<br>GATAAACCCtccGACTACAAGGACGACGATGATAAACATCACCACCATCACCACggaTGATTAACTTACGCCTTAAGCATCATCTTACAGGCGATGAATATGTACAC<br>TAACAAATACGCATAATCAAAATGTGAAAAGTAATACTTTTTTATGAAATAGATTGTTTAACTAATAATATCAATATATCCTTC
