# Supplementary material for: Mitotic checkpoint gene expression is tuned by codon usage bias
Source: EMBO J. 2022 Jul 11;41(15):e107896. doi: 10.15252/embj.2021107896 (PMC9340482; doi:10.15252/embj.2021107896)
Supplement: Supplementary file 2 — Expanded View Figures PDF [file EMBJ-41-e107896-s011.pdf]

Expanded View Figures

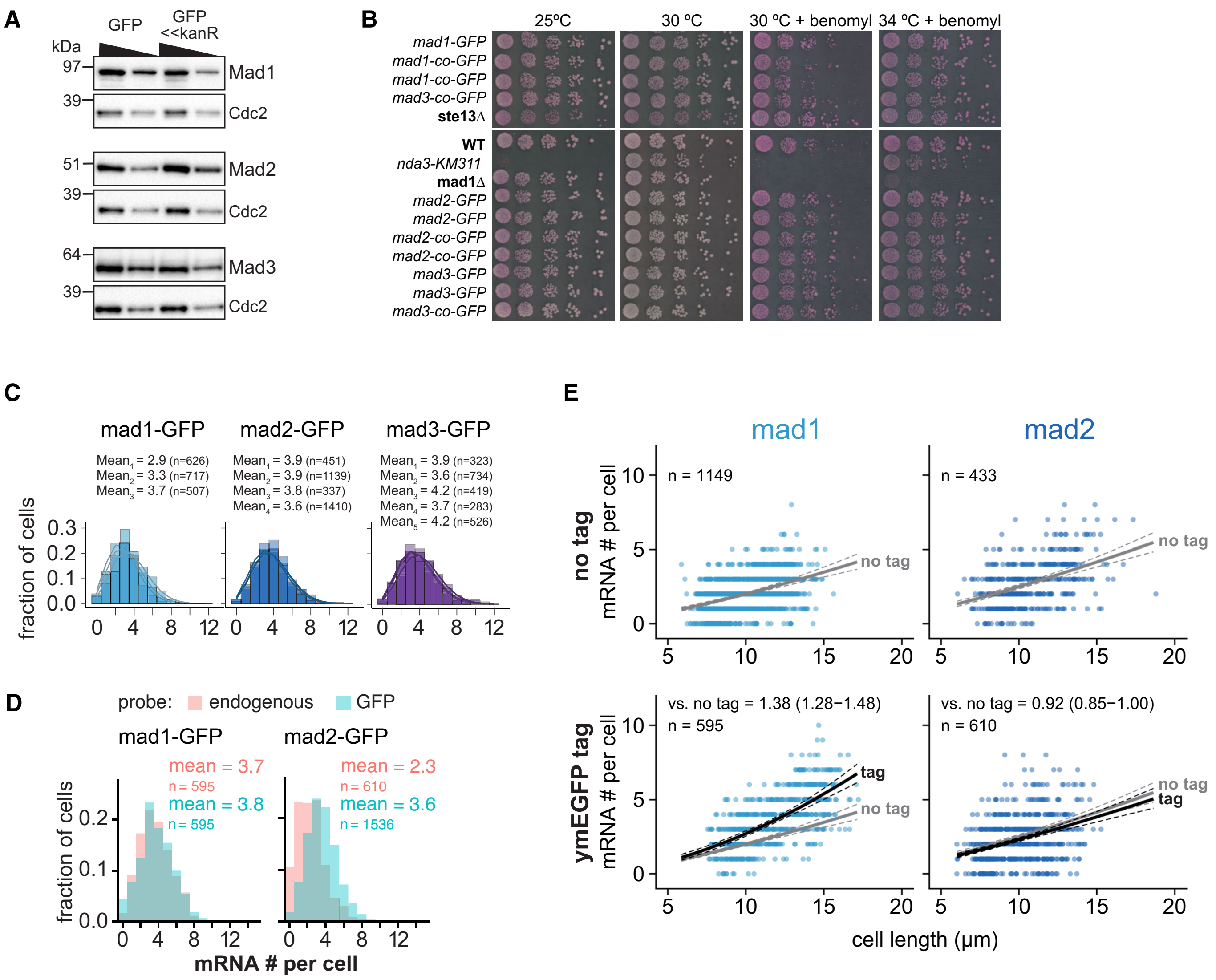

Figure EV1. Additional data on Mad1, Mad2, and Mad3 tagging and mRNA numbers.

- A Immunoblot comparing expression of *mad1*$^{+}$, *mad2*$^{+}$, and *mad3*$^{+}$ tagged at the endogenous locus either by marker-less insertion of yeast codon-optimized monomeric enhanced GFP (ymEGFP, here: GFP) or conventionally with GFP-S65T and a kanamycin-resistance cassette (GFP<<kanR). Antibodies against the endogenous proteins were used. Cdc2 was probed as loading control. A 1:1 dilution is loaded in the second lane for each sample.
- B Growth assay for the indicated strains on rich medium plates without (left side) or with benomyl (right side). The agar contains Phloxine B, which stains dead cells.
- C Frequency distribution of mRNA numbers per cell. Data from individual experiments which are shown combined in Fig 1. Probes were against the GFP portion of each fusion gene. Curves show fit to a Poisson distribution.
- D Frequency distribution of mRNA numbers per cell using probes against the endogenous gene or against GFP in strains expressing a GFP fusion of either *mad1*$^{+}$ or *mad2*$^{+}$. The comparison illustrates that for *mad2*$^{+}$-GFP either the endogenous probe is less sensitive, or there is considerable mRNA degradation from the 5′ end leading to fewer detected spots with a probe on the endogenous gene than on the 3′ end GFP tag.
- E Same experiment as Fig 1E. Scatter plots of whole-cell mRNA counts versus cell length. Solid lines are regression curves from generalized linear mixed model fits (gray for no tag, black for GFP-tagged gene). Dashed lines represent 95% bootstrap confidence bands for the regression curves. Model estimates of the ratio of tagged to untagged mRNA levels with bootstrap 95% confidence intervals are included in the plots. One experiment with probes against *mad1*$^{+}$ or *mad2*$^{+}$ coding sequences, respectively.

Source data are available online for this figure.

 © 2022 The Authors.

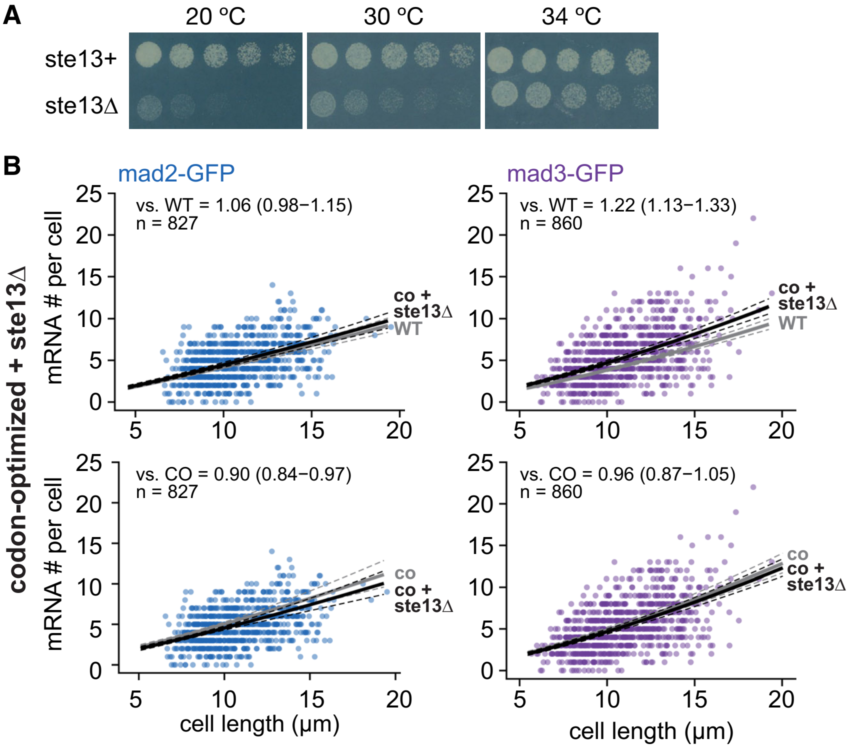

C

mRNA half-life
ste13⁺* vs *ste13Δ

	interaction coefficient (95 % bootstrap confidence interval)	half-life difference (95 % bootstrap confidence interval)	conclusion
mad2	0.039 (0.007 – 0.072)	6.1 min (1.0 – 17.2)	significant
mad3	0.064 (0.021 – 0.107)	4.5 min (1.4 – 13.4)	significant

Figure EV2. Additional data on *mad2⁺* and *mad3⁺* mRNA numbers and half-lives after codon-optimization or *ste13⁺* deletion.

A Growth assay for wild-type and *ste13Δ* cells on minimal medium plates.

B Scatter plots of whole-cell mRNA counts versus cell length for cells expressing codon-optimized mad2- or mad3-GFP and deleted for *ste13⁺*. Solid lines are regression curves from generalized linear mixed model fits; black for the genotype shown, gray for the respective reference: wild type (WT) in the first row, codon-optimized (co) in the second row. Dashed lines represent 95% bootstrap confidence bands for the regression curves. Model estimates of the mRNA level relative to the reference with bootstrap 95% confidence intervals in parentheses are included in the plots. Control curves for upper panels from wild-type *mad2⁺* and *mad3⁺* data in Fig 3D, and for lower panels from codon-optimized mad2 and mad3 data in Fig 3D. Two to five replicates per genotype.

C Statistical significance for mRNA half-life changes after deletion of *ste13⁺*. First and second columns show estimates and 95% bootstrap confidence intervals for the model interaction coefficient and the half-life difference, respectively. The change in half-life after deletion of *ste13⁺* was considered significant if the 95% bootstrap confidence intervals for the interaction coefficient and the half-life difference excluded 0.

Source data are available online for this figure.

© 2022 The Authors.

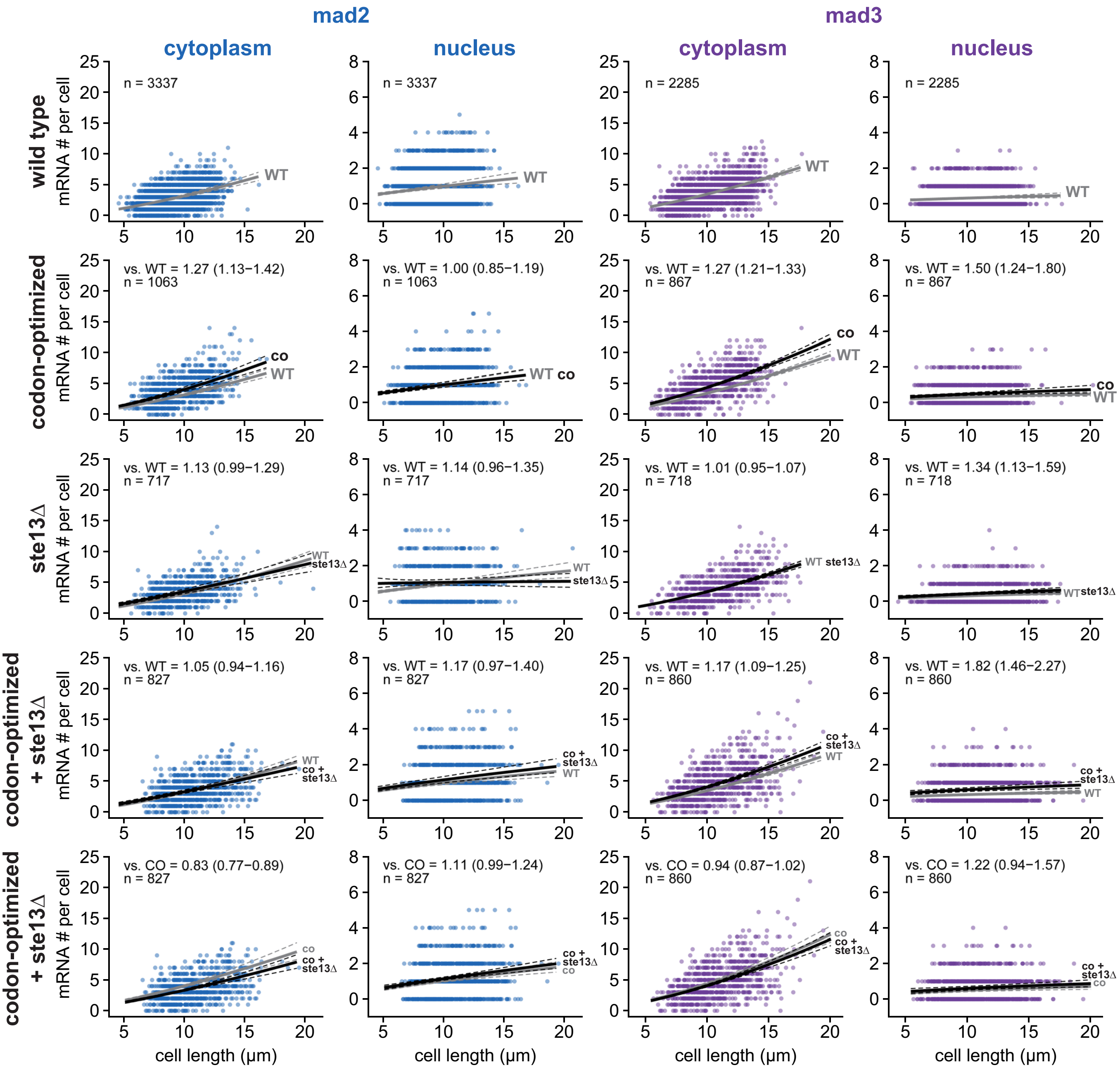

Figure EV3. Cytoplasmic and nuclear FISH data for *mad2*$^{+}$ and *mad3*$^{+}$.

Scatter plots of cytoplasmic and nuclear mRNA counts versus cell length for *mad2*$^{+}$ and *mad3*$^{+}$. Solid lines are regression curves from generalized linear mixed model fits; gray is wild type (WT) in rows 1–4 and codon-optimized (co) in row 5, black is the genotype indicated. Dashed lines represent 95% bootstrap confidence bands for the regression curves. Model estimates for the mRNA ratio between the genotype indicated on the left and the respective reference are included in the plots with bootstrap 95% confidence intervals in parentheses. Same experiments as whole-cell data in Figs 1D and 3D, and EV2B. Two to five replicates per genotype.

Source data are available online for this figure.

© 2022 The Authors.

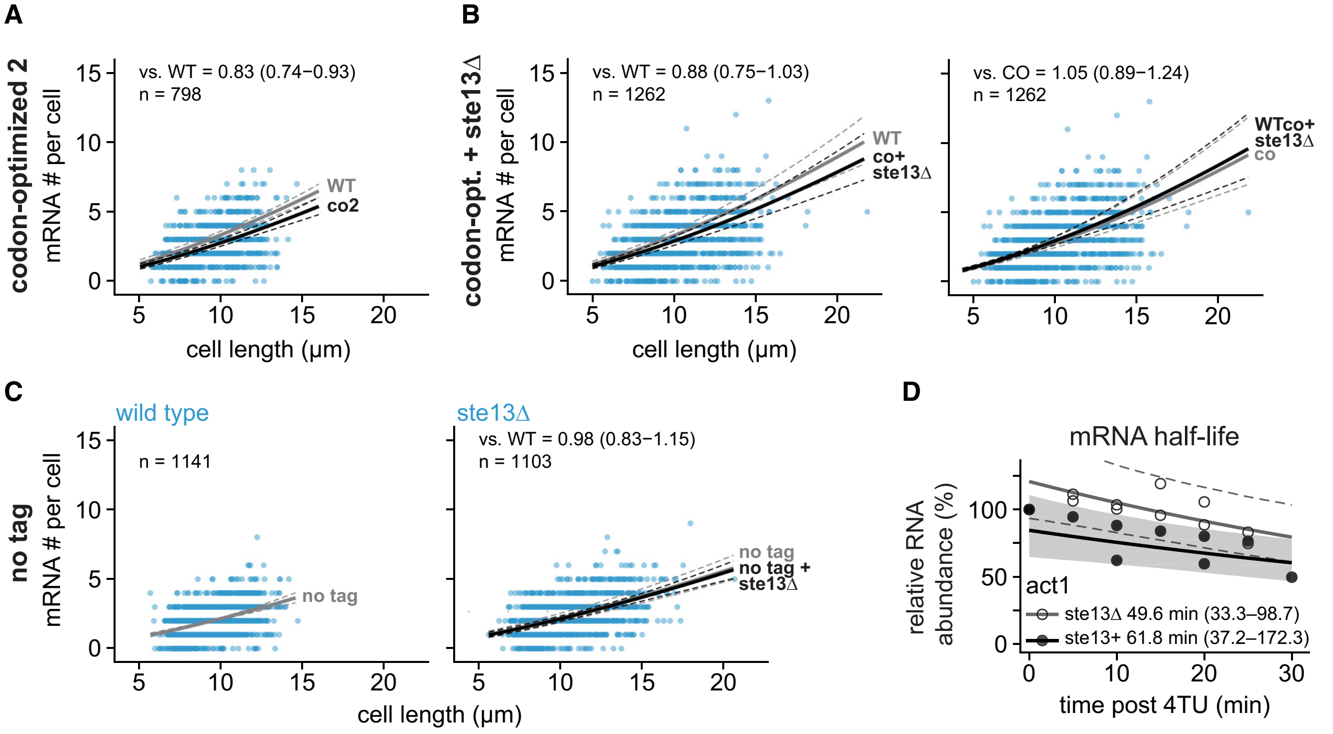

E

mRNA half-life
ste13⁺* vs *ste13Δ

	interaction coefficient (95 % bootstrap confidence interval)	half-life difference (95 % bootstrap confidence interval)	conclusion
mad1	0.052 (-0.003 – 0.108)	4.2 min (-0.2 – 16.5)	not significant
ecm33	0.077 (0.055 – 0.099)	6.5 min (4.1 – 10.5)	significant
act1	-0.003 (-0.013 – 0.007)	-12.2 min (-120.7 – 42.5)	not significant

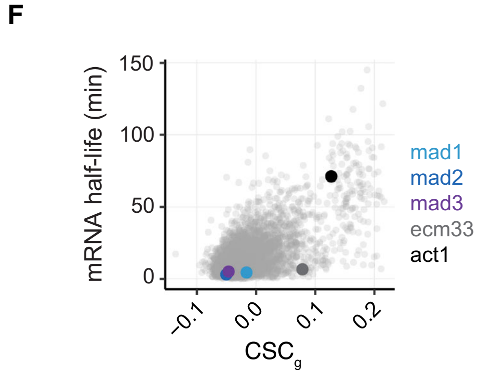

Figure EV4. Additional data on *mad1*$^+$ mRNA number and half-life after codon-optimization or *ste13*$^+$ deletion.

A Scatter plot of whole-cell mRNA counts versus cell length. Solid lines are regression curves from generalized linear mixed model (GLMM) fits; black for the genotype shown (co2), gray for the wild-type (WT) reference. Dashed lines represent 95% bootstrap confidence bands for the regression curves. Model estimates for the mRNA ratio between the genotypes indicated are included in the plots with bootstrap 95% confidence intervals in parentheses. Two to three replicates per genotype.

B As in (A). Black regression line for the genotype shown (co + *ste13Δ*), gray for the respective reference, wild type (WT) or codon-optimized (co). Two to three replicates per genotype.

C Scatter plots for whole-cell mRNA counts of untagged *mad1*$^+$ in *ste13*$^+$ (left) or *ste13Δ* (right) cells, similar to (A). The regression curve for untagged *mad1*$^+$ in *ste13*$^+$ is shown in gray, that for untagged *mad1*$^+$ in *ste13Δ* in black. Probes were against the *mad1*$^+$ coding sequence. One to three replicates per genotype.

D Time course of RNA abundances by qPCR following metabolic labeling and removal of the labeled pool (two independent experiments). Solid lines are regression curves from GLMM fits (black = *ste13*$^+$, gray = *ste13Δ*), excluding the measurements at $t = 0$ to accommodate for noninstantaneous labeling by 4tU. Shaded area is 95% bootstrap confidence band for the *ste13*$^+$ curve and dashed lines indicate 95% bootstrap confidence band for the *ste13Δ* curve. Half-life estimates with 95% bootstrap confidence intervals are included on the plot. The *ste13*$^+$ data are the same as in Fig 2.

E Statistical significance for mRNA half-life changes after deletion of *ste13*$^+$. First and second columns show estimates and 95% bootstrap confidence intervals for the model interaction coefficient and the half-life difference, respectively. The change in half-life after deletion of *ste13*$^+$ was considered significant if the 95% bootstrap confidence intervals for the interaction coefficient and the half-life difference excluded 0.

F CSC$_g$ values (this study) and mRNA half-lives (from Eser *et al*, 2016) for protein-coding *S. pombe* genes with the indicated genes highlighted.

Source data are available online for this figure.

© 2022 The Authors.

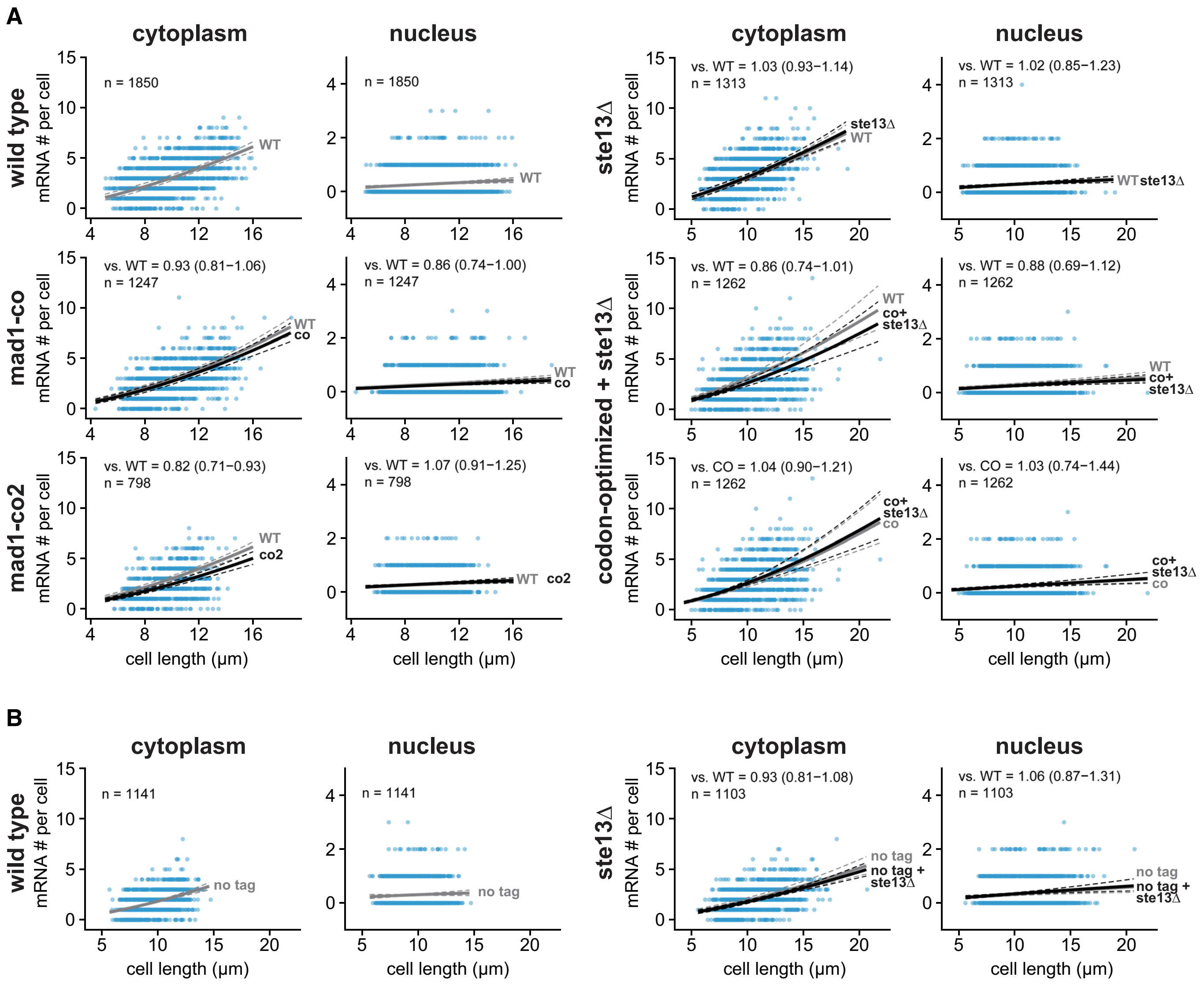

Figure EV5. Cytoplasmic and nuclear FISH data for *mad1*$^+$.

A Scatter plots of cytoplasmic and nuclear mRNA counts versus cell length for *mad1*$^+$. Solid lines are regression curves from generalized linear mixed model fits; gray is wild-type (WT) *mad1*$^+$-GFP for all panels, except the bottom row on the right side, where it is codon-optimized mad1-GFP (co), black is the genotype indicated on the left. Dashed lines represent 95% bootstrap confidence bands for the regression curves. Model estimates for the mRNA ratio between the genotype indicated on the left and the respective reference are included in the plots with bootstrap 95% confidence intervals in parentheses. Same experiments as whole-cell data in Figs 1D and 5B, and EV4A and B. Two to three replicates per genotype.

B Similar to (A) but for untagged *mad1*$^+$. Solid lines are regression curves from generalized linear mixed model fits; gray is untagged wild-type *mad1*$^+$ for all panels, black is untagged *mad1*$^+$ in *ste13Δ*. One to three replicates per genotype. Same experiments as whole-cell data in Fig EV4B.

Source data are available online for this figure.

© 2022 The Authors.

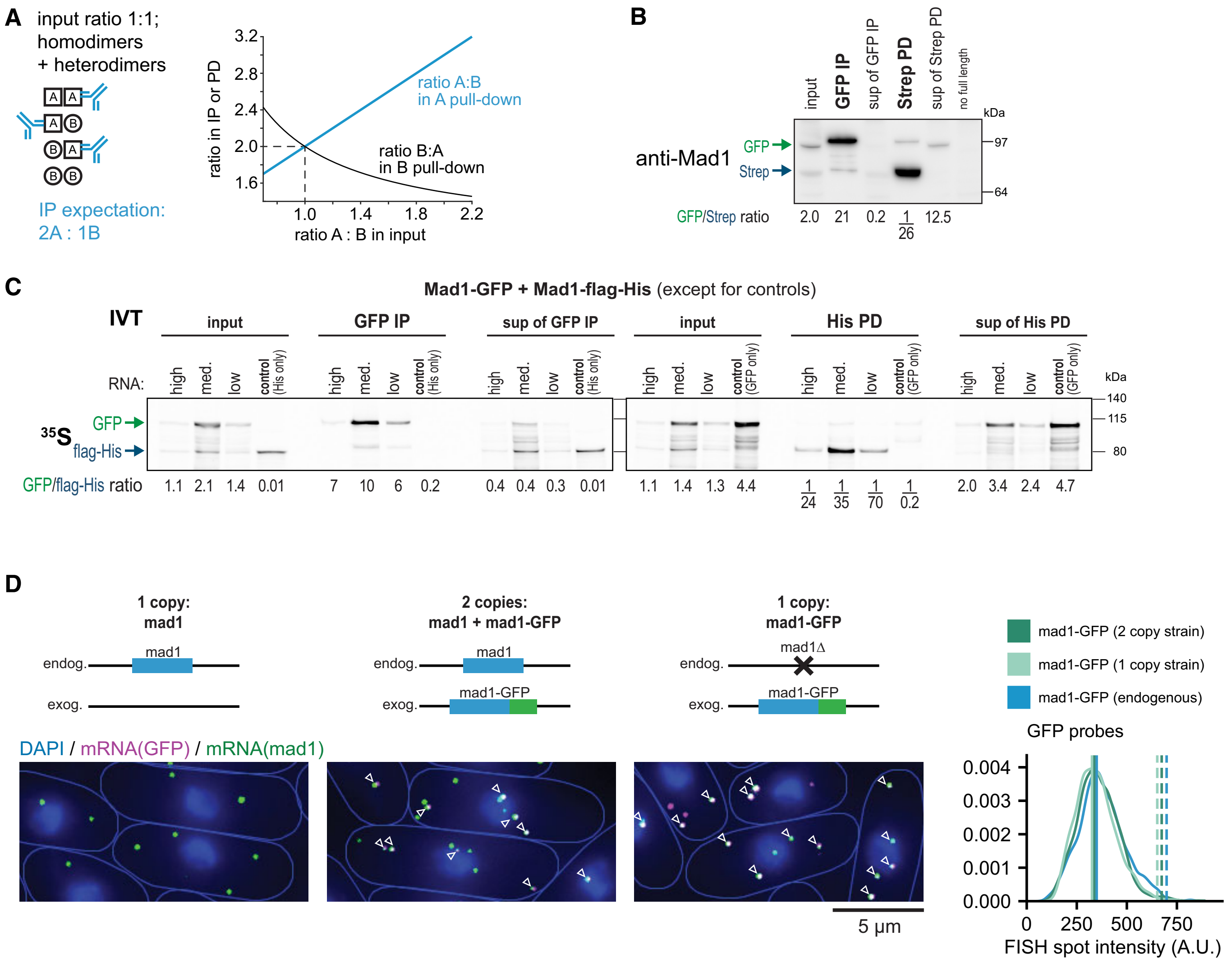

Figure EV6. Additional experiments supporting that Mad1 homodimers assemble co-translationally.

A Theoretical considerations: if, for two different copies of Mad1, homodimer and heterodimer formation was equally likely, and the ratio in the input was 1:1, one would expect a ratio of 2:1 in the pull-down. Expectations for other input ratios are shown in the graph. For typical input ratios in our experiments, the maximum expected ratio in IP/PD is around 4:1, whereas we typically observe 10:1 or higher.

B Replicate experiment for Fig 7C; one of the experiments quantified at the bottom of Fig 7C. Anti-GFP immunoprecipitation (IP) and Strep pull-down (PD) from extracts of diploid cells expressing Mad1-GFP and Mad1-Strep from the two endogenous loci; membrane probed with anti-Mad1; input is 3% of extract used for IP/PD, sup = supernatant. Numbers at the bottom show the quantification of the Mad1-GFP to Mad1-Strep ratio. The last lane contains extract of a diploid strain with both copies of endogenous *mad1*$^{+}$ deleted.

C *In vitro* translation (IVT) of Mad1-GFP and Mad1-flag-His in the presence of ^{35}S-labeled Methionine and Cysteine, followed by GFP immunoprecipitation (IP) or His pull-down (PD); input is 10% of extract used for IP/PD, sup = supernatant. IVTs with only Mad1-flag-His, or only Mad1-GFP were used to control for the specificity of the IP/PD. High RNA conc. is 40 ng/µl mad1-GFP and 35 ng/µl mad1-flag-His; the medium and low concentrations are 1:10 and 1:100 dilutions of the "high" mix. Shown is the autoradiograph after SDS-PAGE with quantification of the Mad1-GFP to Mad1-flag-His ratio. One out of two experiments with similar results.

D Same experiment as in Fig 7E. Representative images from each strain with co-localizing *mad1*$^{+}$ and GFP spots marked by arrowheads. Right side: Intensity of cytoplasmic GFP mRNA spots in the different strains. Vertical solid line: peak of each density plot; dashed line: theoretical position of a double-intensity peak. Number of spots analyzed: *mad1*$^{+}$-GFP (2 copy strain) = 1,178, *mad1*$^{+}$-GFP (1 copy strain) = 1,796, *mad1*$^{+}$-GFP expressed from the endogenous locus (not shown schematically on the left) = 987.

Source data are available online for this figure.

© 2022 The Authors.
