# Supplementary material for: Mitotic checkpoint gene expression is tuned by codon usage bias
Source: EMBO J. 2022 Jul 11;41(15):e107896. doi: 10.15252/embj.2021107896 (PMC9340482; doi:10.15252/embj.2021107896)

Mad1-GFP (mixed with cells not expressing GFP)

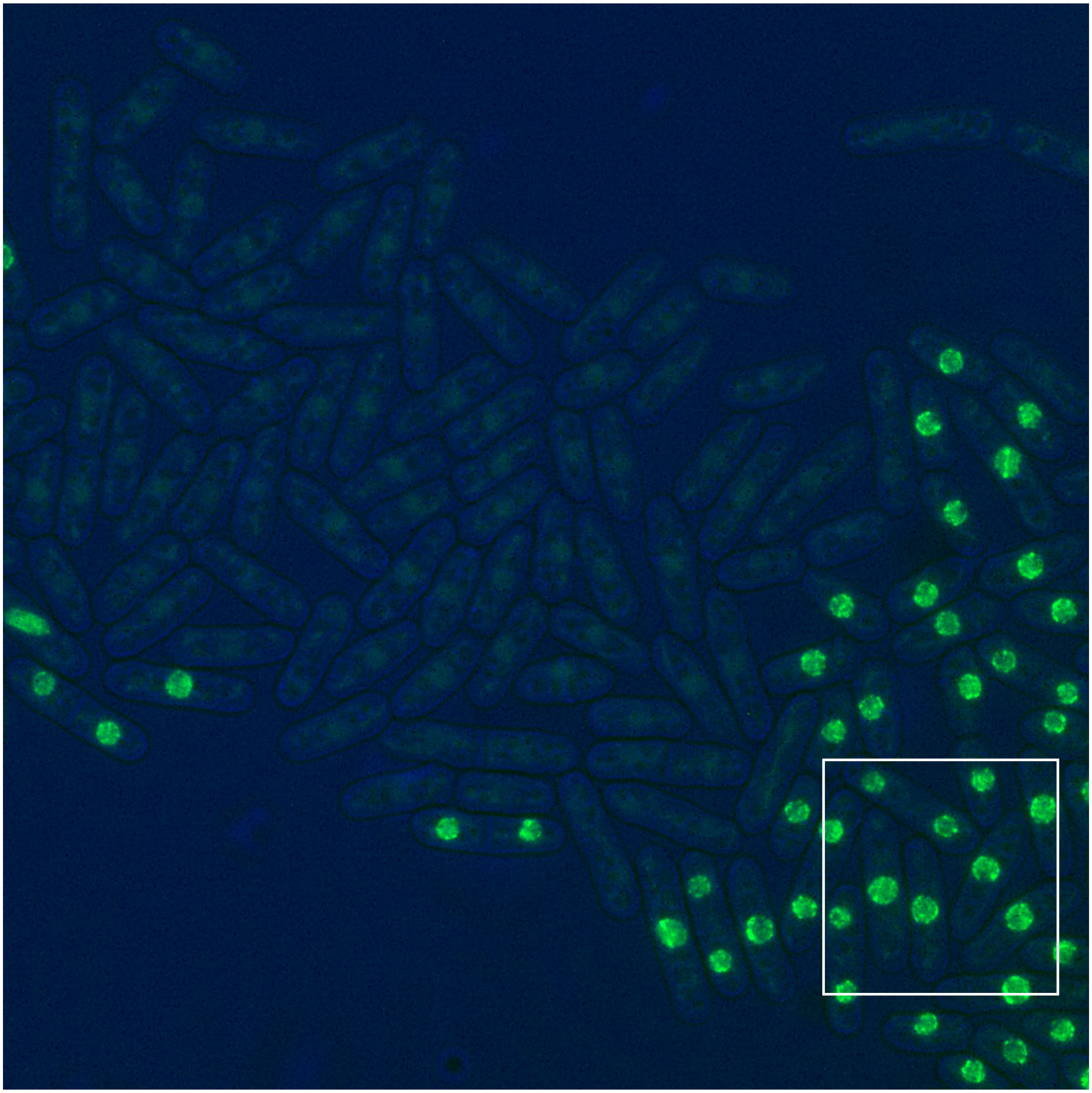

Mad2-GFP (mixed with cells not expressing GFP)

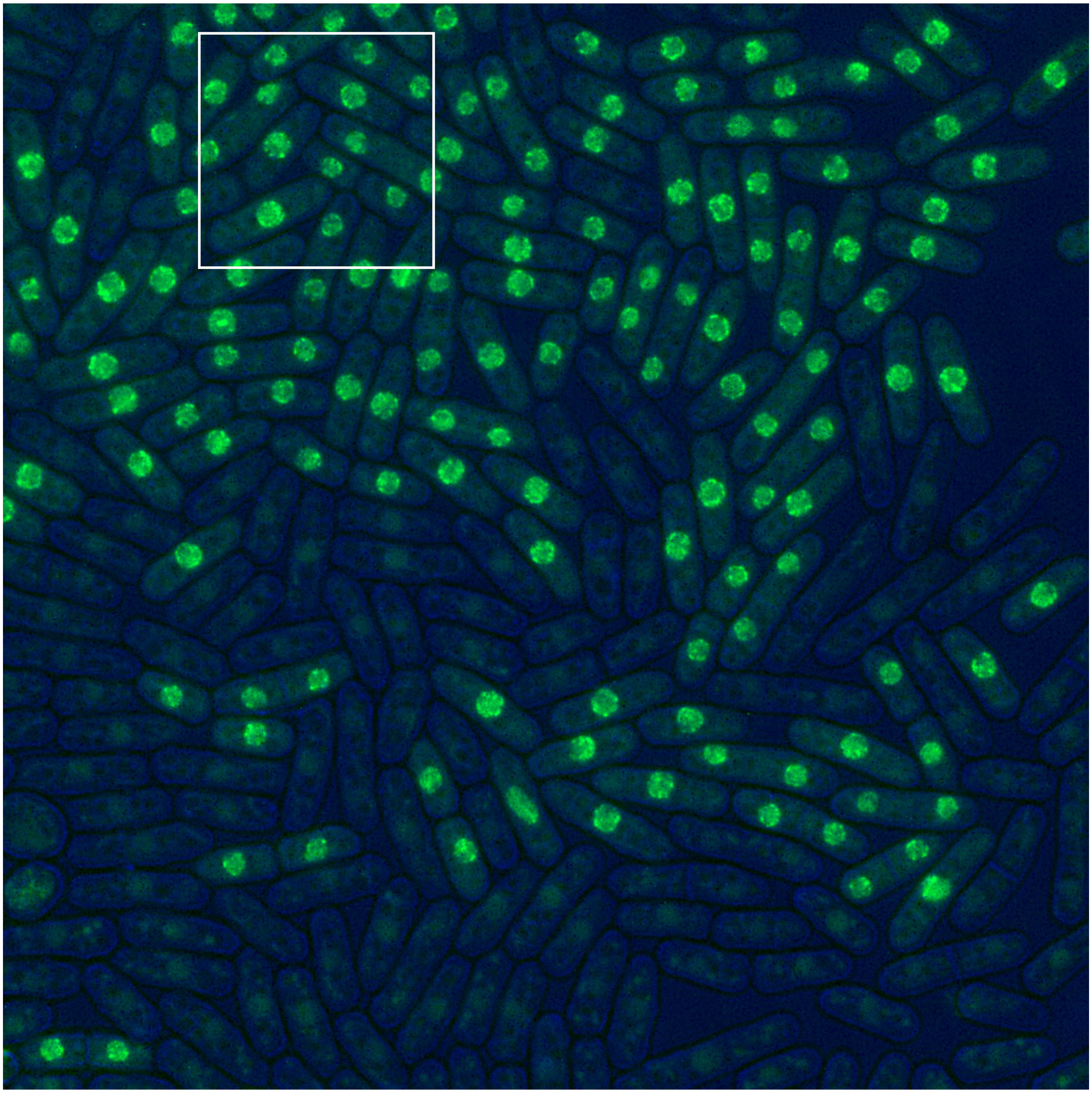

Mad3-GFP (mixed with cells not expressing GFP)

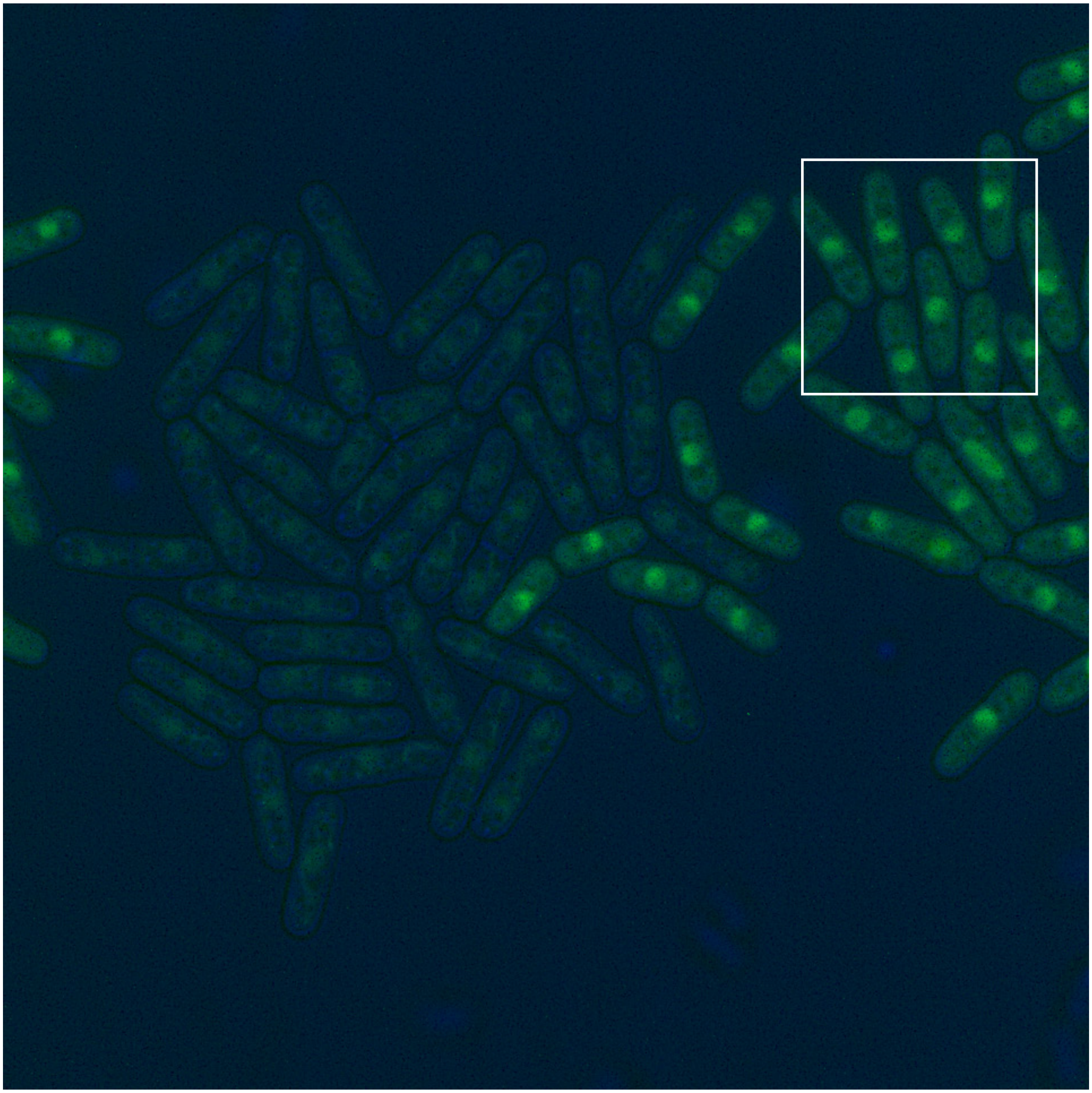

Supplement: Supplementary file 4 — Source Data for Figure 1 [file EMBJ-41-e107896-s005.zip › source_data_fig1/SourceData_Fig_1B_uncropped.pdf]
