# Supplementary figures and images for: Mitotic checkpoint gene expression is tuned by codon usage bias
Source: EMBO J. 2022 Jul 11;41(15):e107896. doi: 10.15252/embj.2021107896 (PMC9340482; doi:10.15252/embj.2021107896)

mad1-GFP

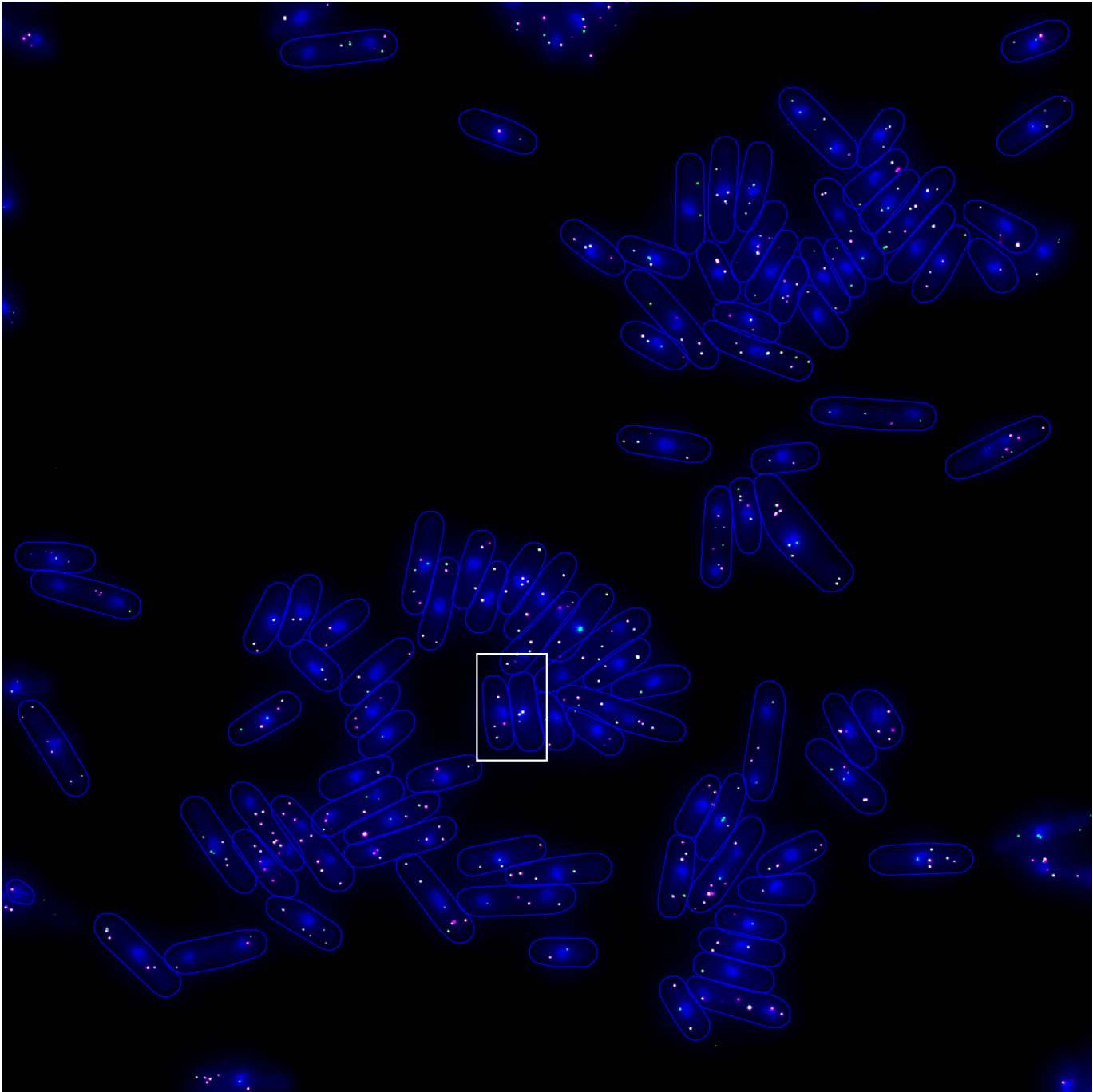

mad1 mad2-GFP

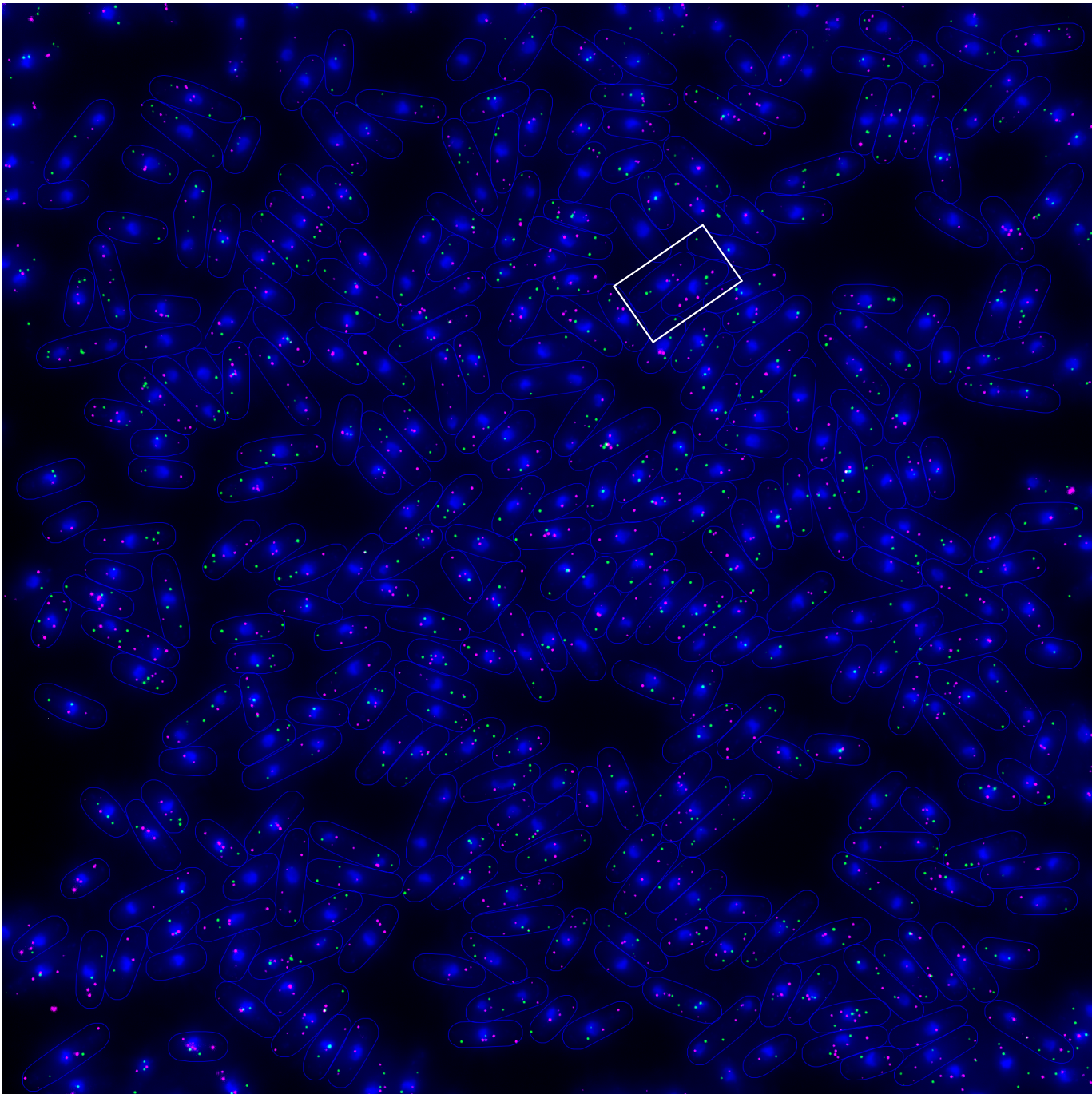

Supplement: Supplementary file 4 — Source Data for Figure 1 [file EMBJ-41-e107896-s005.zip › source_data_fig1/SourceData_Fig1F_uncropped.pdf]

Mad1-GFP

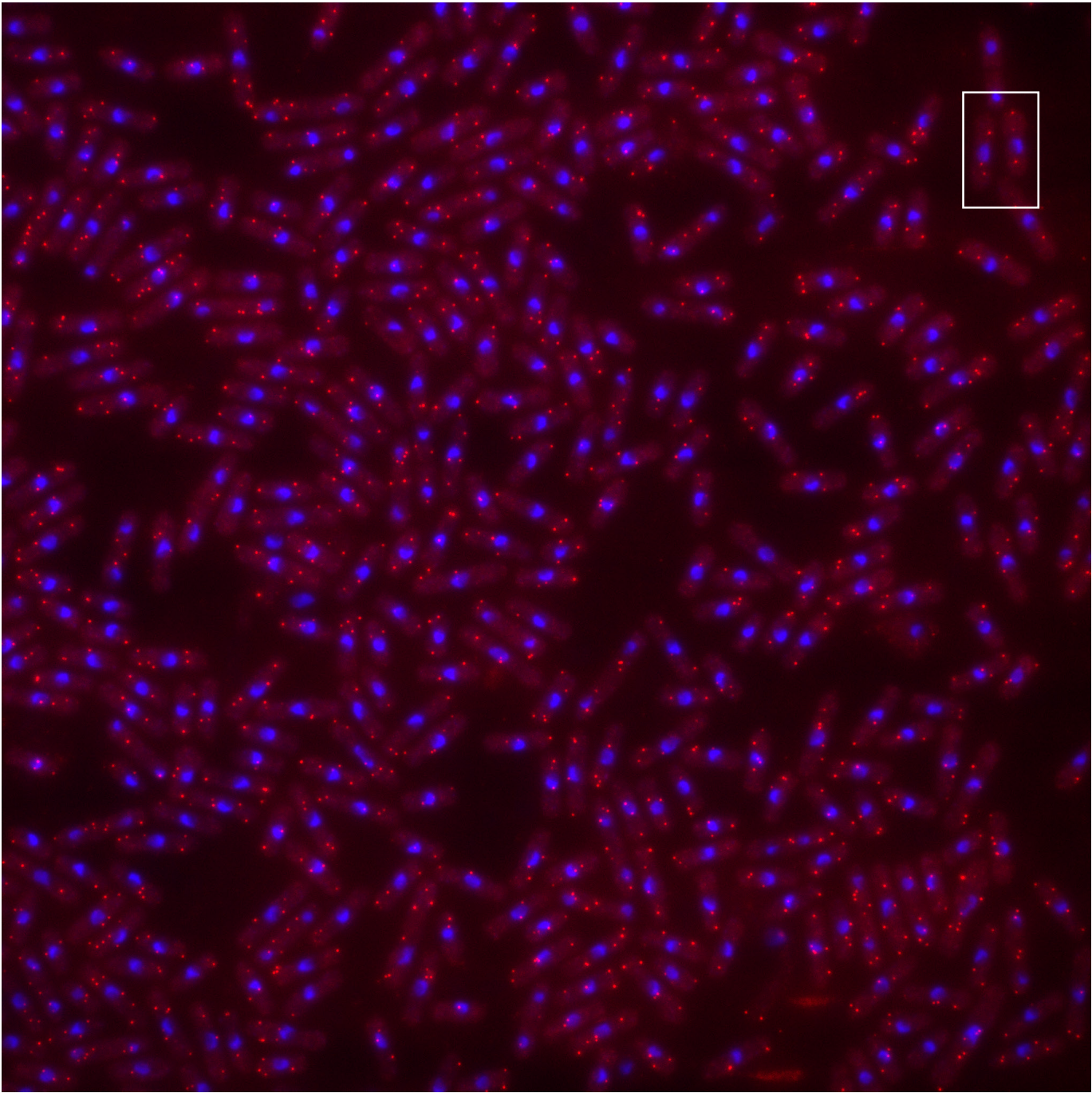

Mad2-GFP

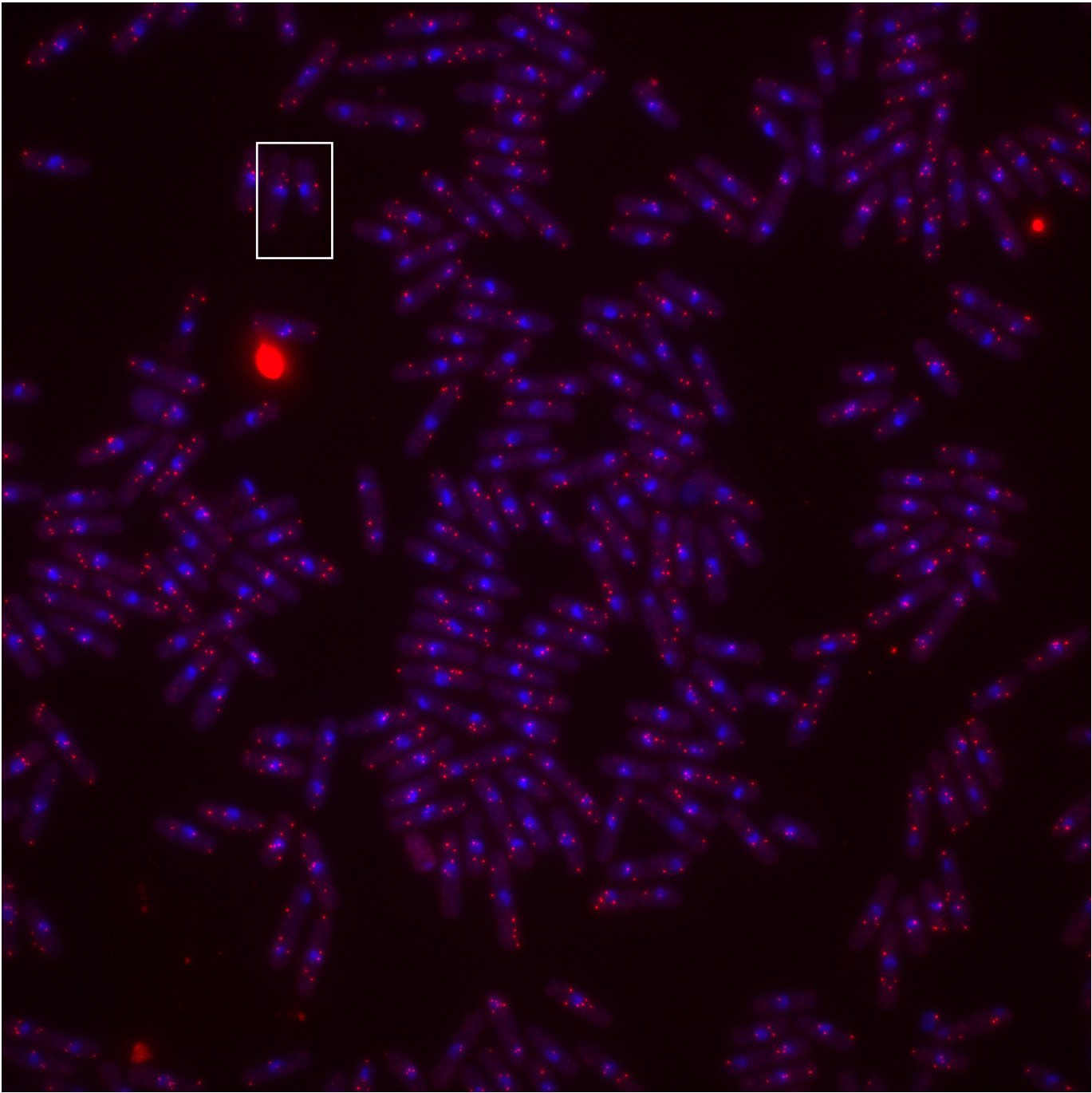

Mad3-GFP

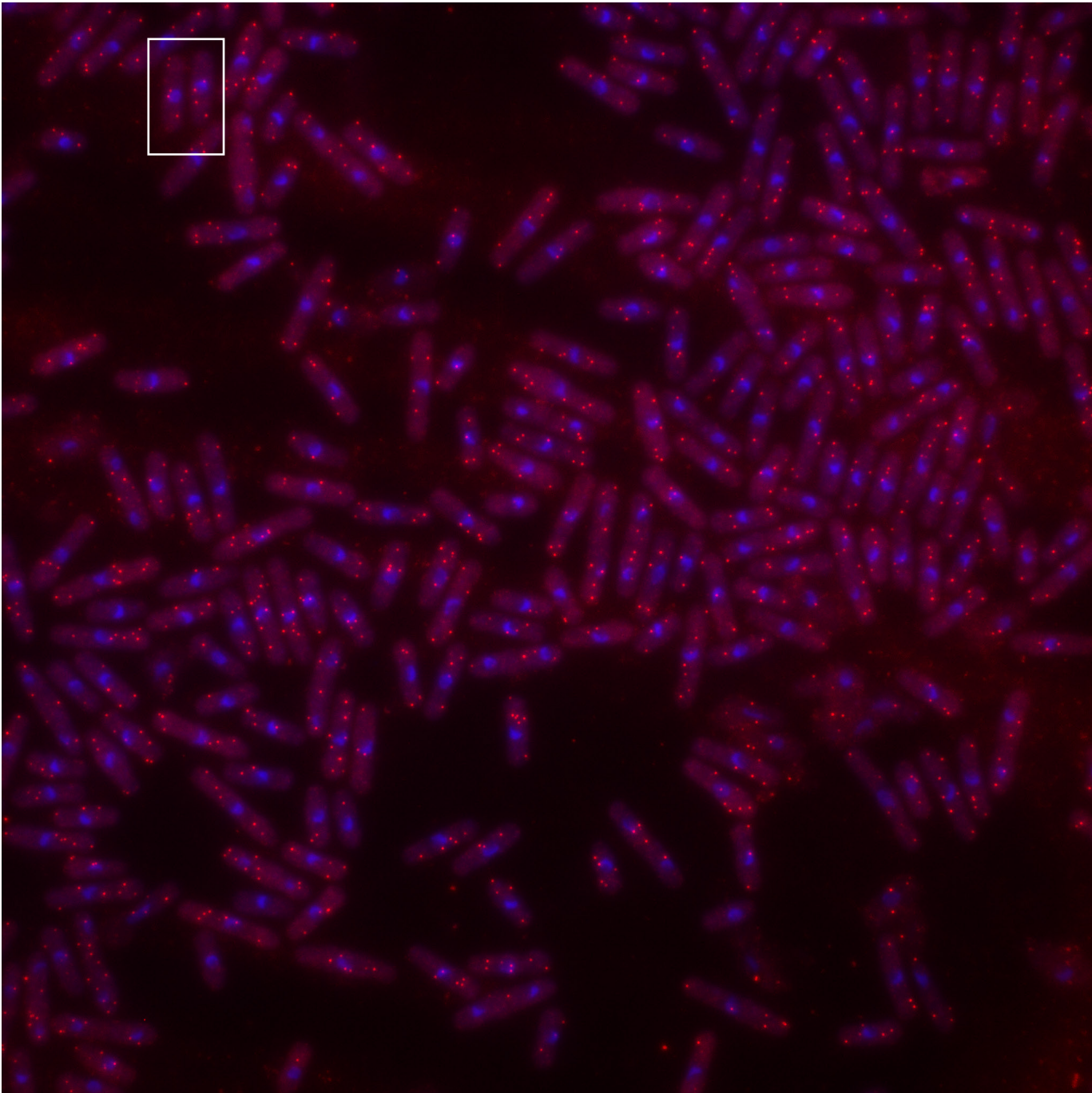

Supplement: Supplementary file 4 — Source Data for Figure 1 [file EMBJ-41-e107896-s005.zip › source_data_fig1/SourceData_Fig_1C_uncropped.pdf]

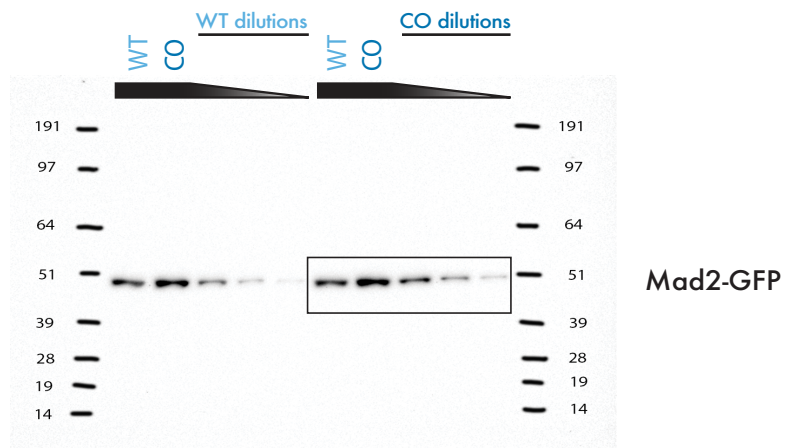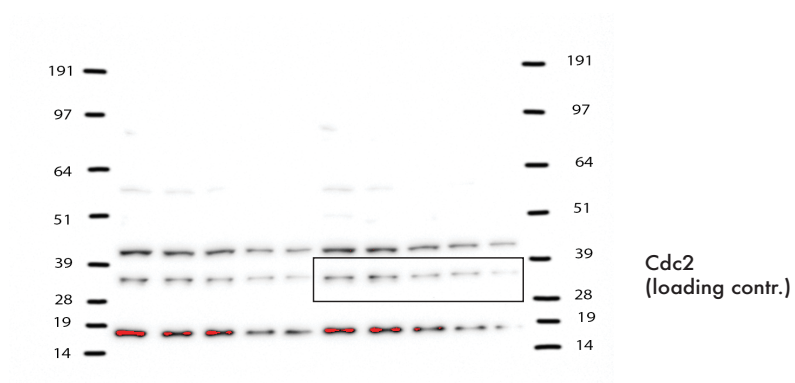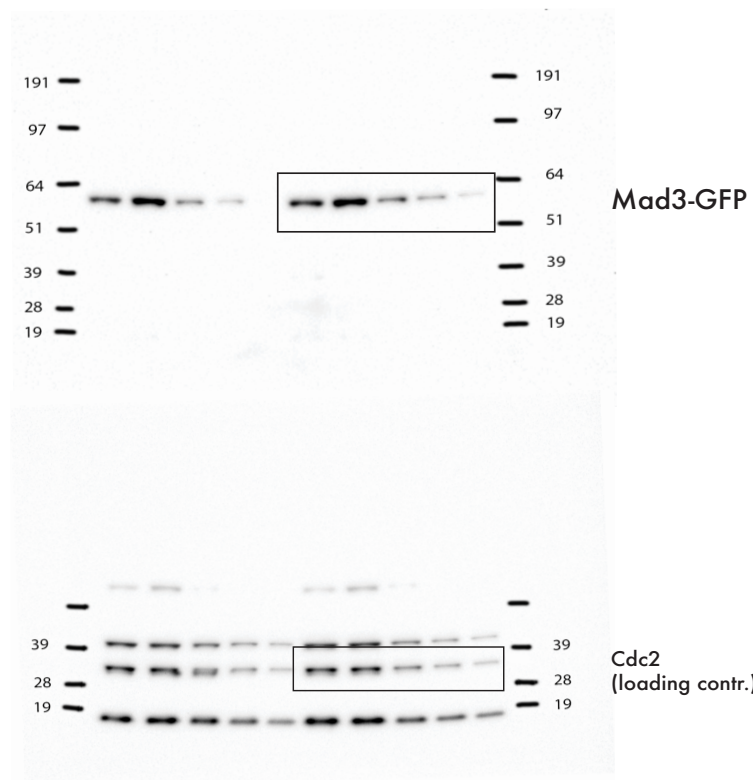

Supplement: Supplementary file 7 — Source Data for Figure 4 [file EMBJ-41-e107896-s008.zip › source_data_fig4/SourceData_Fig_4A_uncropped.pdf]

Membrane was cut before antibody incubations

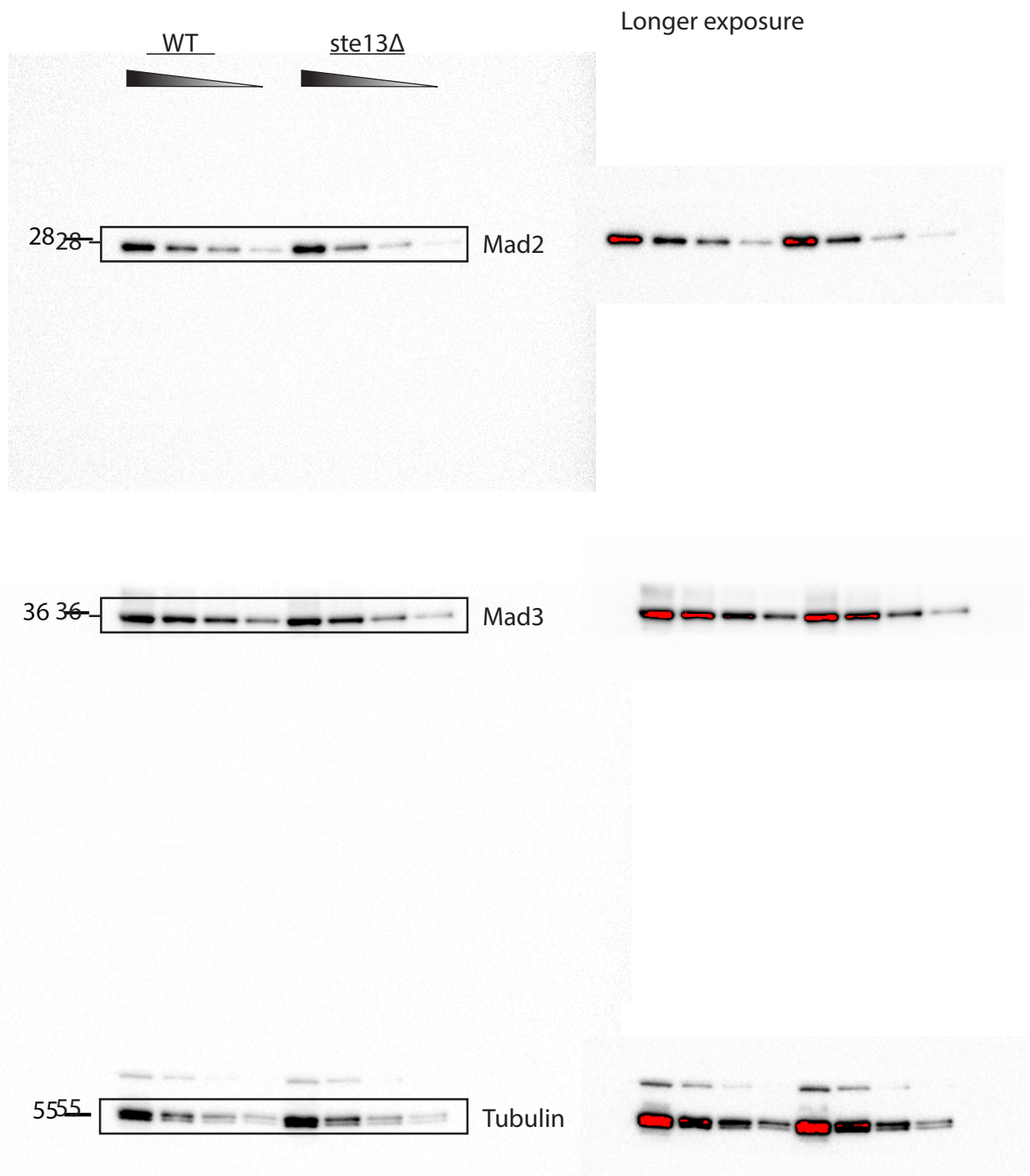

Supplement: Supplementary file 7 — Source Data for Figure 4 [file EMBJ-41-e107896-s008.zip › source_data_fig4/SourceData_Fig_4B_uncropped.pdf]

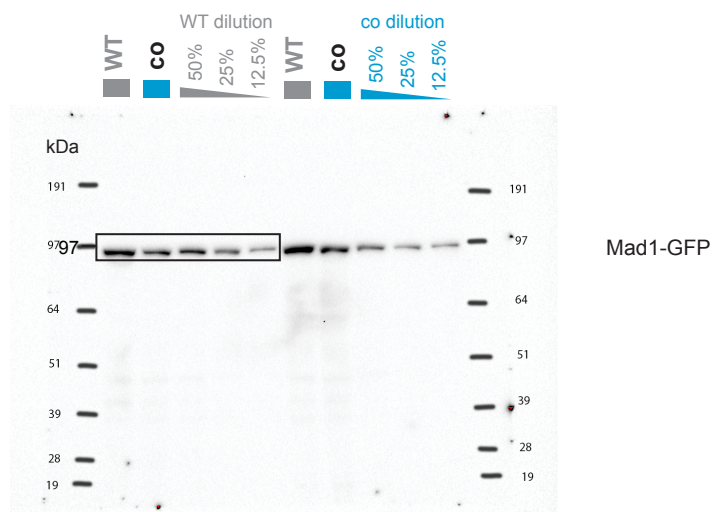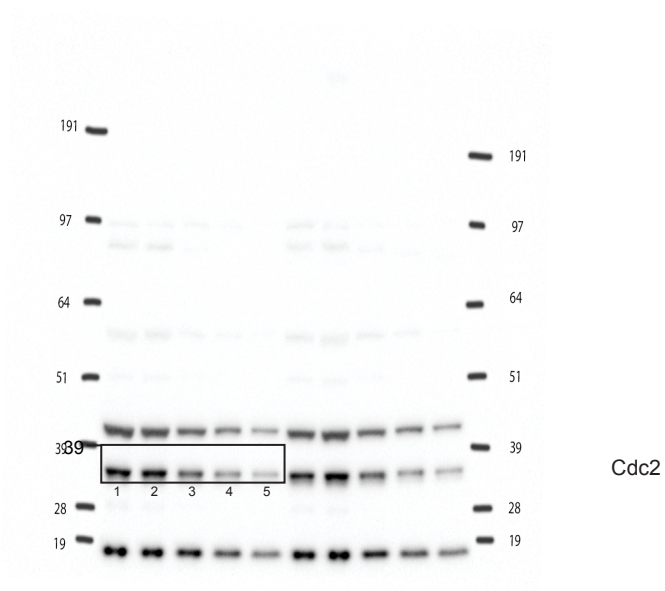

Supplement: Supplementary file 9 — Source Data for Figure 6 [file EMBJ-41-e107896-s001.zip › source_data_fig6/SourceData_Fig_6A_uncropped.pdf]

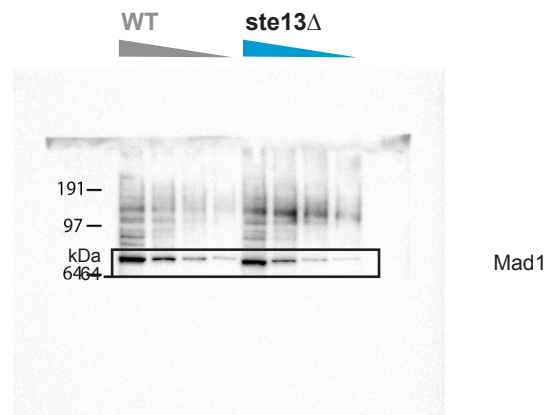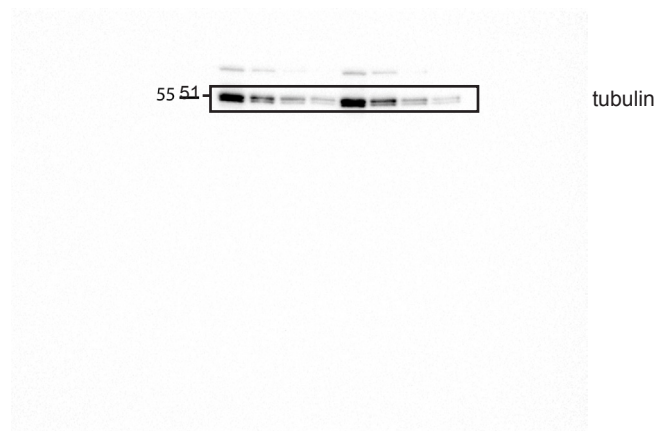

Supplement: Supplementary file 9 — Source Data for Figure 6 [file EMBJ-41-e107896-s001.zip › source_data_fig6/SourceData_Fig_6B_uncropped.pdf]

Mad1-WT-GFP  
(mixed with GFP-negative cells)

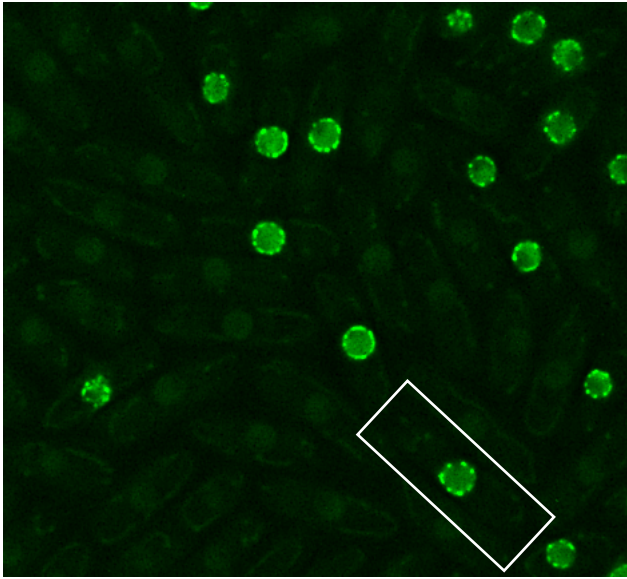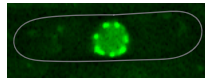

Mad1-co-GFP  
(mixed with GFP-negative cells)

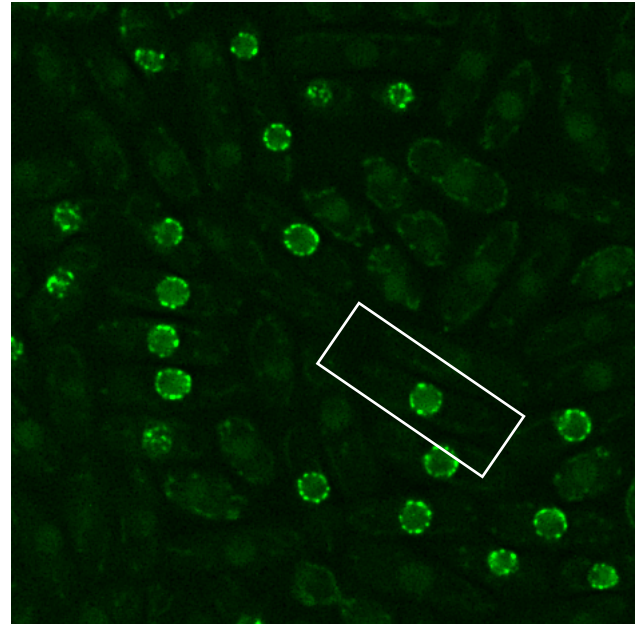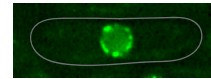

Supplement: Supplementary file 9 — Source Data for Figure 6 [file EMBJ-41-e107896-s001.zip › source_data_fig6/SourceData_Fig_6E_uncropped.pdf]

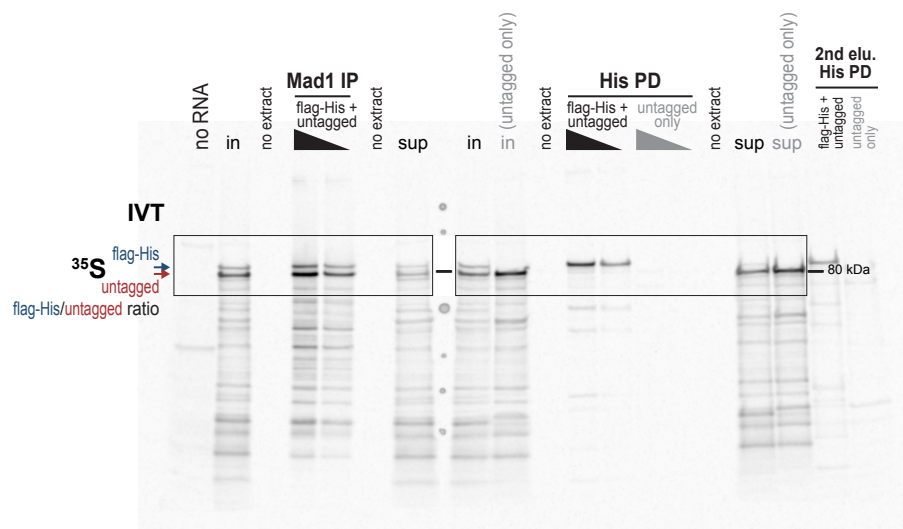

Supplement: Supplementary file 10 — Source Data for Figure 7 [file EMBJ-41-e107896-s003.zip › source_data_fig7/SourceData_Fig_7D_uncropped.pdf]
