# Supplementary material for: Mitotic checkpoint gene expression is tuned by codon usage bias
Source: EMBO J. 2022 Jul 11;41(15):e107896. doi: 10.15252/embj.2021107896 (PMC9340482; doi:10.15252/embj.2021107896)

Mad2-WT-GFP
(mixed with GFP-negative cells)

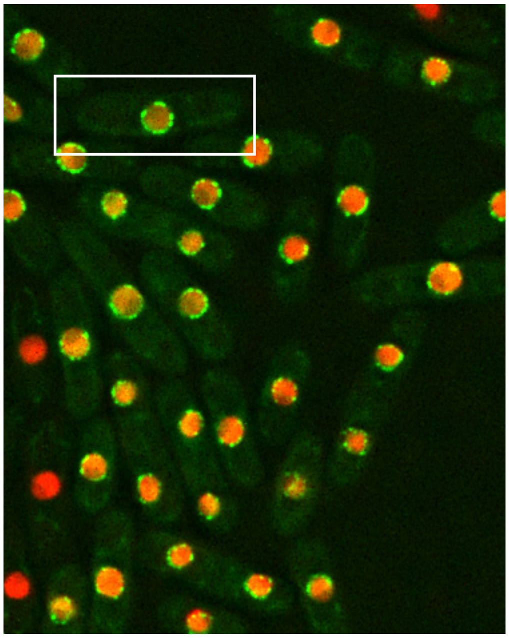

Mad2-co-GFP
(mixed with GFP-negative cells)

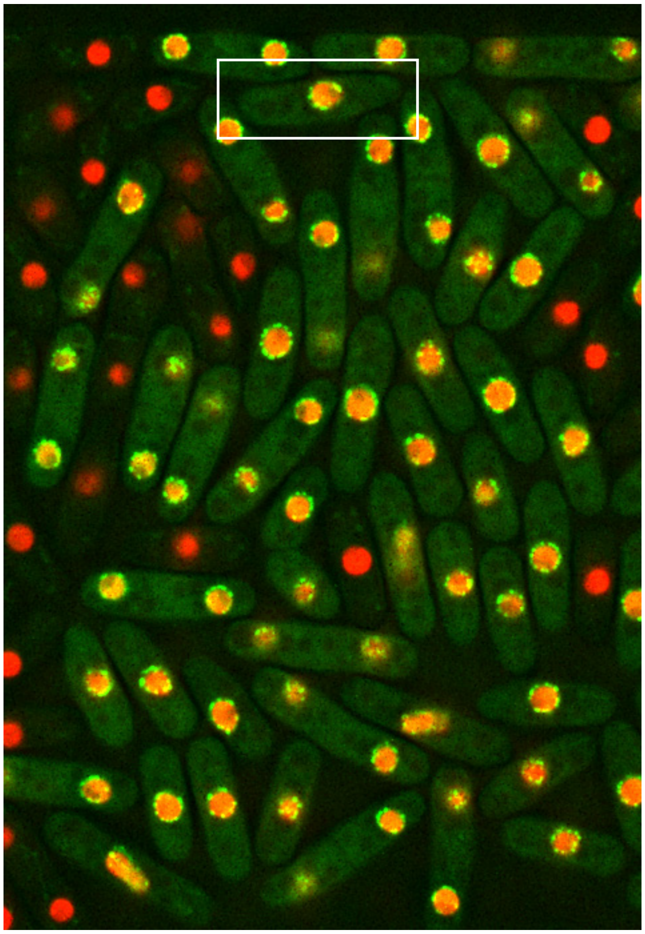

Mad3-WT-GFP
(mixed with GFP-negative cells)

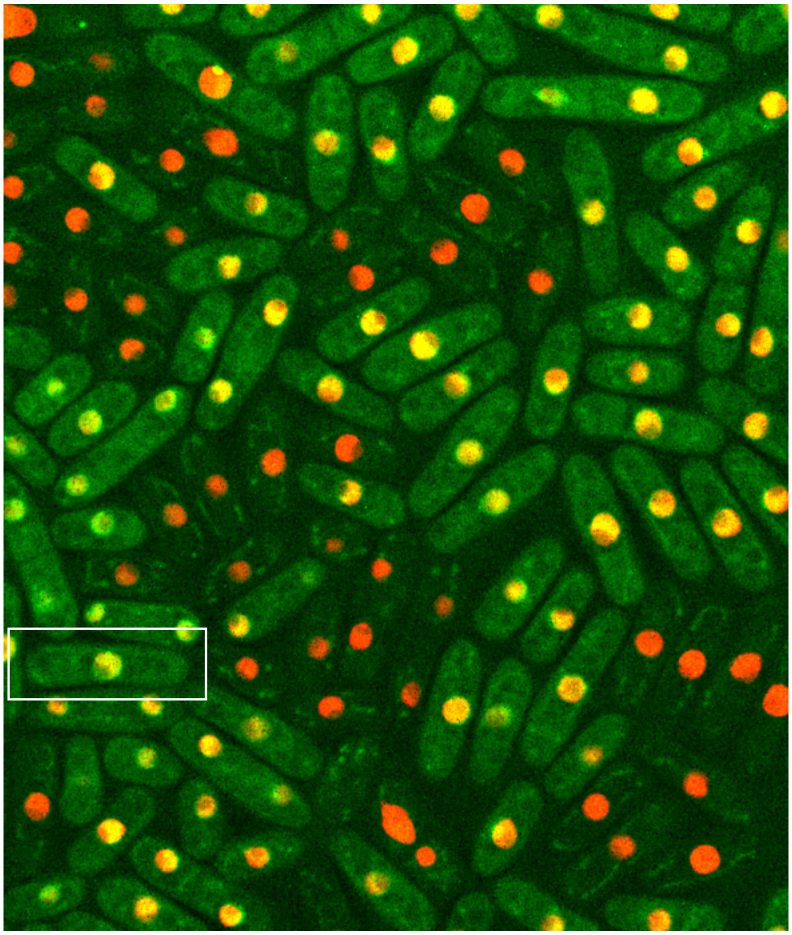

Mad3-co-GFP
(mixed with GFP-negative cells)

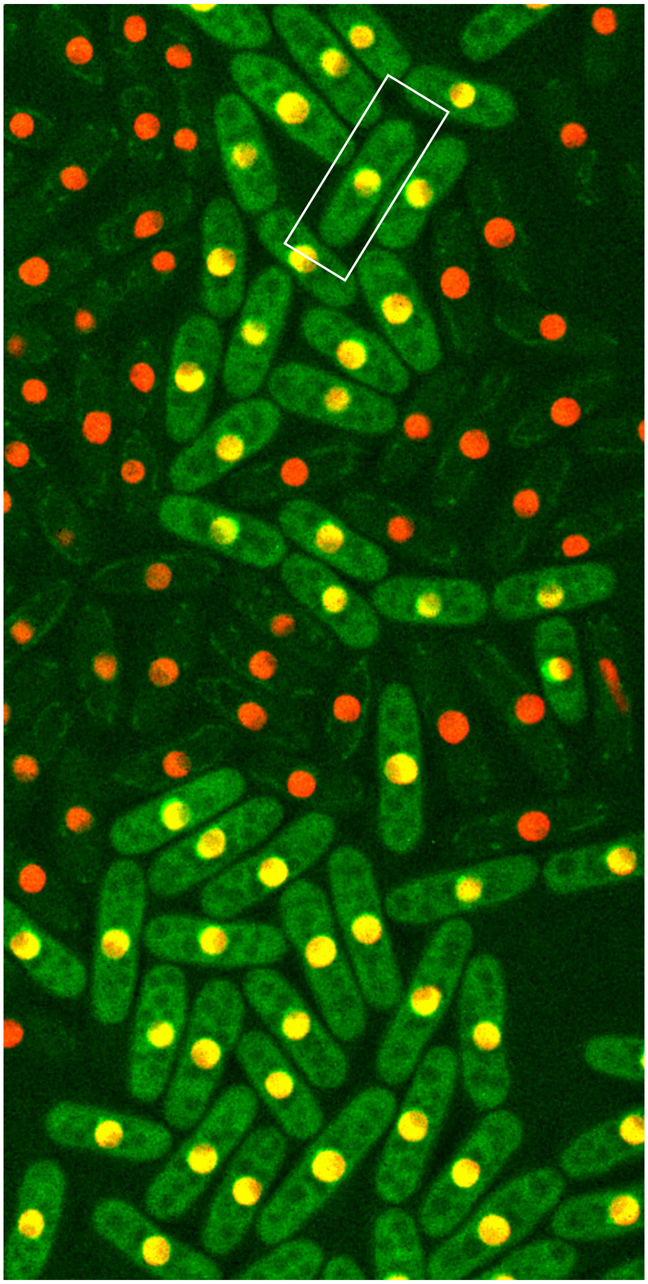

Supplement: Supplementary file 7 — Source Data for Figure 4 [file EMBJ-41-e107896-s008.zip › source_data_fig4/SourceData_Fig_4E_uncropped.pdf]
