# Supplementary material for: Mitotic checkpoint gene expression is tuned by codon usage bias
Source: EMBO J. 2022 Jul 11;41(15):e107896. doi: 10.15252/embj.2021107896 (PMC9340482; doi:10.15252/embj.2021107896)

Left side (GFP IP)
Membrane cut for different antibody incubations

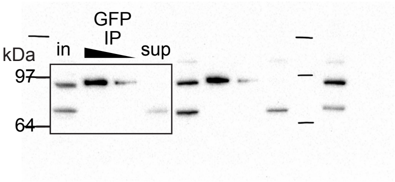

(anti-Mad1)

Ponceau

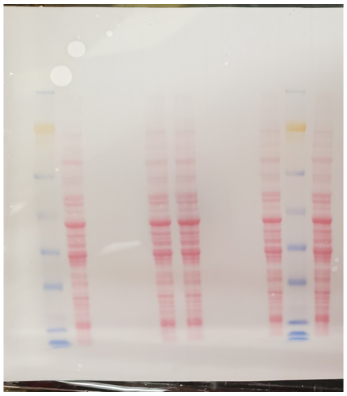

Righ side (Strep pull-down)
Membrane cut for different antibody incubations

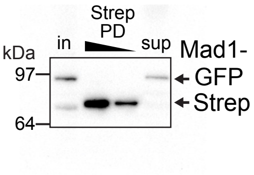

(anti-Mad1)

Ponceau

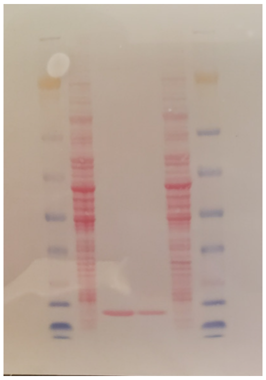

Supplement: Supplementary file 10 — Source Data for Figure 7 [file EMBJ-41-e107896-s003.zip › source_data_fig7/SourceData_Fig_7C_uncropped.pdf]
