# Supplementary material for: Mitotic checkpoint gene expression is tuned by codon usage bias
Source: EMBO J. 2022 Jul 11;41(15):e107896. doi: 10.15252/embj.2021107896 (PMC9340482; doi:10.15252/embj.2021107896)
Supplement: Supplementary file 11 [file EMBJ-41-e107896-s010.pdf]

THE EMBO JOURNAL

Mitotic checkpoint gene expression is tuned by codon usage bias

Eric Esposito, Douglas Weidemann, Jessie Rogers, Claire Morton, Erod Keaton Baybay, Jing Chen, and Silke Hauf
DOI: 10.15252/embj.2021107896

Corresponding author(s): Silke Hauf (silke@vt.edu)

Review Timeline:

Submission Date:	31st Jan 21
Editorial Decision:	16th Mar 21
Revision Received:	10th Apr 22
Editorial Decision:	20th May 22
Revision Received:	30th May 22
Accepted:	6th Jun 22

Editor: Stefanie Boehm

Transaction Report:

(Note: With the exception of the correction of typographical or spelling errors that could be a source of ambiguity, letters and reports are not edited. Depending on transfer agreements, referee reports obtained elsewhere may or may not be included in this compilation. Referee reports are anonymous unless the Referee chooses to sign their reports.)

1st Editorial Decision 16th Mar 2021

Thank you again for submitting your manuscript for consideration by The EMBO Journal, as well as for providing a pre-decision point-by-point response to the reviewer comments. As outlined in your preliminary response, in the revised version, it will be crucial to add further experimental data to support the proposed model of co-translational Mad1 homodimer assembly (ref#1- point 6; ref#2- point 6), as well as further addressing the functional role of the proposed regulatory mode for spindle assembly checkpoint function and cell cycle regulation (ref#3- point 4). In addition, statistical analyses appropriate to the respective experimental context, as well as sufficient detail on experimental procedures, simulations and analyses must be provided throughout the manuscript. Please also carefully respond to all other referee comments and revise the manuscript accordingly, in particular also to ensure that all conclusions are sufficiently supported by data and potential remaining alternate models discussed.

Please note that it is our policy to allow only a single round of major revision. We realize that lab work worldwide is currently affected by the COVID-19/SARS-CoV-2 pandemic and that an experimental revision may be delayed. We can extend the revision time when needed, and we have extended our 'scooping protection policy' to cover the period required for a full revision. However, if you encounter any issues that may significantly delay a revision, please contact us to discuss this as soon as possible.

Referee #1:

The work by Esposito et al. addresses the contribution of codon optimality to mRNA stability and protein expression. This is a concept that has been explored numerous times in the past and the author's results confirm previous findings. This work focuses on yeast (pombe) mad1-3 mRNAs, showing that codon composition contributes to define mad 2-3 mRNAs half-lifes, but not for mad1. This work is mostly technically sound and well performed.
Most of the results presented confirm previous studies, i.e. definition of mRNA and protein expression levels, subcellular localization, influence of codon optimalitu, effect(s) of GFP fusion, coupling of mRNA half-lives and protein stability to determine gene expression peaks etc. The authors do mention the literature and how the results they obtain are consistent with previous works.
There are several potentially new findings (detailed below), but they are not developed to a point where new conclusions can be derived. At the present point, the parts of this work that are "finished" are mostly confirmatory and the newest findings would need a substantial amount of work before they are ready for publication.

Major points:

1- Figures 1, 2 and 3A-B, while relevant for following experiments, confirm published studies.
2- Fig 3C-E and 5, while new for mad1-3 mRNAs, confirm what has been shown for other mRNAs.
3- Fig 4. The impact of mRNA stabilization on protein level changes is very modest (unclear if it is statistically significant), suggesting a buffering mechanism. This is even more sticking if the one assumes the improving codon optimality should also increase translation. It would be more relevant to directly measure translation instead of steady state protein levels.
4- Authors show that low codon optimality is required but not sufficient to regulate mRNA stability, and just cite a published work (Eser, 2016) for 3' UTR cis acting elements without further exploring the interplay between both events. Defining these elements and the interplay with codon optimality could have been a new and interesting mechanistic finding, but is not pursued.
5- Authors combine codon optimality with stet13 depletion, with some apparently contradictory results. However the described effects are expected from previous works showing that codon optimality impacts on mRNA stability upstream of dhh1 (webster 2018) and that codon optimality and mRNA stability are linked through Not5 (buschauer 2020).
6- The authors attempt to link mad1-3 mRNAs stability and translation to dimer assembly by colocalization and coinmunoprecipitation experiments. They conclude that it may be relevant for Mad1. Although the co-IP experiments are consistent with this hypothesis, this approach is not sufficient to prove this point. A much more detailed study would be required.

Minor points

1- The effects of GFP limit the strength of some of the conclusions (i.e. line 100-103, fig. 1D and Fig. S1D), maybe they could try to fuse Mad1 to another fluorescent protein or non-fluorescent tag.
2- In fig. 3D where the authors analyze the mRNA amount of mad2/3-GFP per cell, I would expect that the wt mad2/3-GFP would have increased mRNA/cell in the delta ste13 mutant, similar to that of the codon optimized Mad2/3-GFP, this is not shown in panel 3D, what is shown is the WT mRNA in WT cells.
3- Mad2/3 protein concentrations decrease in the delta ste13 mutant (fig. 4B 4C, lines 208-210), do the tagged proteins, both codon optimized or not, also decrease levels in the ste13 mutants?
4- Authors conclude that Mad1 IP precipitates the same amount of Mad1 and Mad1-GFP, I would say that precipitates more Mad1-GFP (line 255, fig. 6G)

Referee #2:

In this excellent study, Esposito and colleagues show that fission yeast cells maintain consistent levels of three checkpoint proteins: Mad1, Mad2, and Mad3/BubR1, by tuning mRNA and protein stability. The authors find that the low cell-to-cell variance in protein levels for the three genes arises through low mRNA stability coupled with high protein lifetime. Surprisingly, the authors find that non-optimal codon usage is responsible for the low stability of all three mRNA, but via two distinct mechanisms. In the case of Mad2 and Mad3, the mRNA molecules appear to be degraded via a known cellular pathway, whereas the low stability of Mad1 mRNA points to more complex and intriguing mechanism.

Overall, the manuscript presents well-designed experiments and thorough, thoughtful analyses and discussions of their outcomes. The findings presented shed light on a new aspect of the regulation of protein levels involved in spindle assembly checkpoint, and this regulation may well be shared by other proteins in fission yeast and other eukaryotes. Therefore, the manuscript will be of great interest for many cell biologists, and I fully support its publication. I do have a few minor questions and comments that the authors should address either by adding more discussion or by conducting new experiments and/or analyses.

1. The correlation between cell cycle, cell length, and transcript number per cell: Given that Mad1-3 are checkpoint signaling

proteins, it is natural to expect that their expression is likely to be cell-cycle regulated. In fact, data exist documenting the cell-cycle dependence of the expression of these proteins in other eukaryotes. The authors should discuss the potential for cell-cycle regulation in the context of fission yeast. Mad1-Mad2 appear to have roles in nuclear import-export outside of mitosis in budding yeast (work from the Wozniak lab). Mad2 plays other significant roles in human cells (work from the Yu lab), whereas Mad3 is only thought to be important for checkpoint signaling. Therefore, there expression of the three proteins may be subject to cell-cycle-dependent regulation to differential degrees.

On a related note, I think that there is an inconsistency between the strong correlation between cell size and transcript number per cell (Fig. S1) and the Poisson fit with a single rate parameter shown in Fig. 1. Since the average transcript number per cell increases with cell size, doesn't this indicate that underlying synthesis and decay rates are changing? A possible explanation could be chromosome duplication following the S-phase. The authors can address this issue by suitably modifying the discussion.

2. Fig. 2A - it is not clear to me whether the CV was calculated based on the nuclear signal, the cytoplasmic signal, or the sum of the two for the three proteins. Does the value of the coefficient change based on which pool is considered? Additionally, both Mad1 and Mad2 localize strongly to the nuclear envelop, whereas Mad3 does not do so. Are there interesting correlations between the magnitudes of the nuclear, cytoplasmic, and nuclear membrane pools? This analysis is not essential, but it will be of interest to those who work in the checkpoint field.

3. Minor comment - I found is somewhat surprising that the Mad2 intensity in the nucleus appears to be ~ 1.5x lower than that of Mad1 (Fig. S3A). Mad2 must be in excess of Mad1 for mitotic checkpoint signaling according to the current understanding. A comment on this puzzling observation will be useful.

4. Another observation that deserves some discussion is that the amount of Mad2 is greater than that of Mad3 even though the protein and mRNA turn-over rates are comparable for the two proteins. Are the differences in these rates large enough to explain the significant difference in protein levels? The authors should be able to comment on this using their simulations.

5. Minor issue - In budding yeast, human cells, and in C. elegans, attaching a GFP to the N or the C-terminus is known to impair protein function. It is somewhat surprising to me that this is not the case in fission yeast, given that the homologs are structurally very similar. The authors do show that the SAC is functional as per the benomyl sensitivity assay, but this assay may not be sensitive enough to detect impaired Mad2 function. The authors may want to note this possibility in their discussion.

6. Co-IP of Mad1-GFP and Mad1: The possibility of co-translational folding and homodimerization of Mad1 and the role of the Mad1 transcript in promoting this is a really intriguing hypothesis. However, the evidence presented in its support (Fig. 6G-I) is not entirely convincing. Especially in the Mad1-GFP/Mad1 co-IP experiment, an appreciable amount of Mad1 does precipitate with Mad1-GFP. This is consistent with post-translational dimerization of the two proteins. The results of the Mad1-GFP/Mad1-strep experiment appear to be more in line with the co-translational dimerization hypothesis. The authors should comment on this discrepancy. This hypothesis is not the focus of the study, so the following experiment is optional. To rigorously test their hypothesis, the authors should consider an experiment based on in vitro translation. With in vitro translation, the authors should be able to titrate the mRNA relative to the ribosomal capacity and thus observe directly whether co-translational dimerization is significant on Mad1 transcripts.

7. The role of transcriptional control on Mad2 and Mad3 levels: For the sake of completeness, the authors should note the role of Mad2 and Mad3 promoters in setting average transcript levels. This is important because the promoters must maintain a constant and low rate of transcription to enable the mRNA and protein turn-over rates to stabilize protein levels.
To highlight the role of transcriptional regulation, the authors should consider contrasting the result of Mad2 or Mad3 replacement with GFP with the surprising results of Mad1 replacement shown in Fig. 7.

8. In some of the figures (e.g. Fig 6), information about the amount of protein loaded on the immunoblot is missing (e.g. Fig. 6B, G, I).

9. A more detailed description of the overall calculation scheme behind the dynamic simulations presented in Fig. 2B would be useful. I may have overlooked this information, but I could only find a reference to the script used.

Referee #3:

In the study "Mitotic checkpoint gene expression is tuned by coding sequences" Esposito and colleagues study how the expression of core spindle assembly checkpoint proteins is controlled to ensure accurate protein stoichiometries. The authors discover that codon usage tunes the expression level of mad2 and mad3 but not mad1 in fission yeast. Notably, a high frequency of non-optimal codons leads to short half-life of mad2 and mad3 mRNAs, which in combination with long protein half-lives contributes to accurate protein stoichiometry. The authors show that cells deploy a different, albeit unresolved strategy to keep mad1 mRNAs short lived and protein levels well controlled.

How cells control stoichiometries of proteins is a poorly understood yet general problem in cell biology. Perhaps the most puzzling remains how the stoichiometries of different proteins that form complexes are regulated such that cells produce enough but not too much of each individual component of the complex. The SAC is a good example and an excellent starting point in addressing this important question as its components are well characterized, their interactions well documented, and consequences of perturbed homeostasis prominent and detrimental for cell survival. The reviewer, therefore, finds this study relevant, timely, and of broad interest.

The authors make novel claims and provide a comprehensive investigation showing that the expression of the SAC genes is controlled at the level of mRNA and protein stabilities. They show in computational simulations and in cells that a combination of short mRNA half-lives and long protein half-lives provides stable and accurate protein quantities. The short mRNA half-lives are attributed to high frequency of sub-optimal codons. This finding is further corroborated in codon-optimization experiments, where mRNA half-lives are prolonged resulting in increased protein concentrations. However, the comparison of mRNA half-life between unperturbed and codon-optimized strains remains somewhat confusing and would profit from revised data interpretation and presentation as discussed below.

Overall, the manuscript is clearly written and easy to follow, and the figures well-prepared, with a few minor comments discussed below. All experiments are well-documented, and appropriate controls are provided, but most experiments have been performed in duplicates rather than triplicates, and statistical analyses are missing throughout the manuscript as discussed below.

Even though it remains to be identified how Mad1 mRNA and protein levels are controlled, the reviewer fells that this work does represent an important step forward in deciphering how cells control protein quantities, in this case of the SAC complex via regulated mRNA and protein stability.

Major comments

1. The authors provide exciting new insights into how regulated mRNA and protein stabilities result in accurate stoichiometries of the core components of the SAC. However, the data are lacking statistical analyses throughout the manuscript, which the authors should provide in support of their conclusions.

2. Closely related to the previous comment, most experiments throughout this manuscript have been performed in two replicates, and a few rare ones even as just one. To strengthen their claims, and their statistical analyses, the authors should provide data from the third biological replicate.

3. One of the main conclusions of the work presented in this manuscript is that codon usage bias contributes to short half-lives of mad2 and mad3, but not mad1 mRNAs. To support this claim, the authors measure half-lives of these mRNAs. However, the measurements presented in Fig. 2D, Fig. 3E, and Fig. 5C do not seem to reflect the measurements reported in the related text. The authors should revise the text and figures to provide consistency. In addition, they should provide R values for the fits and perform statistical analyses to support their claims on the effect of codon optimization on mRNA stability.

4. In the abstract, as well as introduction (lines 76-78) and discussion lines (287-288) the authors present a tempting suggestion that the codon usage of SAC genes provides a feature that promotes proper function of the SAC. This claim, however, remains unaddressed in the manuscript. To support their statement, the authors should ideally test if the SAC works well in the strains where mad2 and mad3 sequences have been codon-optimized using the benomyl resistance assay (as in Fig. S1B). Adding another set of mutants that encode more suboptimal codons (the reverse of codon-optimization) and the analysis of their resistance to benomyl would further strengthen their claims. Alternatively, and at the very least, the authors should clearly indicate that these are speculations.

Minor comments:

1. There seems to be an irrelevant self-citation in line 69 (Heinrich et al, 2013). This article seems inappropriately cited again in the results section (line 94) where it is referenced as a source of their previous mRNA number measurements using FISH, which doesn't appear to be present in the cited article.

2. The authors state that the efficiency of the mad2-specific FISH probe was lower than that one of the GFP-specific probe. However, it is unclear how they measure the probe efficiency to draw this conclusion. The authors should provide explanation either in the main text or in the materials and methods.

3. The authors should make sure to properly introduce the abbreviations throughout the manuscript.

4. The reviewer recommends that the authors state explicitly in the manuscript text that the data presented in Fig. 2B are from a simulation. This figure is a little hard to read - for example, it is not evident what the x- and y- axes represent. Clarity would be

improved if the authors provided a more elaborated caption within the figure legend.

5. Fig. S7E is wrongly called in text.

6. Measurements of the mad1 mRNA half-life reveal a much noisier pattern than the other two. Is it possible that this higher noise in mRNA levels is relevant for protein quantity control? Or is this noise a consequence of some technical challenges? The authors could touch on this briefly in their discussion.

7. The materials and methods section contains information on Mad1-GFP localization on kinetochores, which appears irrelevant to this manuscript.

We are grateful to all three reviewers for their constructive comments.

We have substantially revised the manuscript based on the suggestions. The major changes and additions are:

(1) We provide substantial additional evidence for co-translational assembly of Mad1 homodimers.
   a. Additional experiments and new quantifications show that the ratio between the isoform that is being pulled on vs. the other isoform is substantially larger than expected if homodimer and heterodimer formation occurred with equal likelihood (Fig 7A, 7C, EV6A, EV6B). This is true regardless of which isoform is being pulled on.
   b. We demonstrate that we see the same in *in vitro* translations (Fig 7D, EV6C).
   c. We show that the bias does not only occur in the presence of the large GFP tag but is also seen when combining Mad1 with a short tag and untagged Mad1 (Fig 7D).
   d. We find no evidence for mRNA co-localization either for one single isoform or between two isoforms (new mRNA FISH experiments in Fig 7E,F)

(2) We have revised almost all our statistical analyses. Since the experiments were a mix of biological, experimental, and technical replicates, we decided, in consultation with our university's Statistical Applications and Innovations Group, that generalized linear mixed models (GLMM) are best suited to model the data. Bootstrapping was used to define the 95 % confidence interval.

   To increase statistical power, we have added several new smFISH experiments for wild-type and ste13D strains.

   We distinguished between biological and experimental replicates in the following way: a biological replicate uses a different strain (expected to have the same genotype, but different from the one initially used), whereas an experimental replicate is a repeat experiment on a different day with the same strain. Technical replicates are repeats of a late step in the procedure with the same material (e.g. re-running an SDS-PAGE or different slides in a FISH experiment). All levels of replicates are now reflected in the generalized linear mixed model (GLMM). Overall numbers are given in the legends, the full information is available in the source data.

   Since mRNA number scales with cell size, and cell size can vary slightly between experiments, we now base all our conclusions regarding changes in mRNA number on cell size-corrected data that was fit by a GLMM (Fig 3, 5, 7, EV1-5).
   New figures show regression lines with 95 % confidence interval from the GLMM, as well as the estimated percent change relative to the reference along with the 95 % confidence interval (see for example Fig 3D,E and 5B,C).

(3) We have employed an additional, more sensitive test for spindle assembly checkpoint (SAC) function by deleting the microtubule-interacting protein Alp7. The results suggest a slightly impaired SAC when codon-optimized *mad1* is expressed (Fig 6F). This supports that synonymous mutations may affect the SAC.

All the changes have led to considerable modifications in Fig 3 and 5, entirely replacing Fig 7, and a major re-writing of the text. The former Fig 7 is now Fig S5A, B. The key conclusions remained unchanged.
We provide a text file that has the major changes highlighted in blue for easier re-reviewing.

Referee #1 (Report for Author)

The work by Esposito et al. addresses the contribution of codon optimality to mRNA stability and protein expression. This is a concept that has been explored numerous times in the past and the author's results confirm previous findings. This work focuses on yeast (pombe) mad1-3 mRNAs, showing that codon composition contributes to define mad 2-3 mRNAs half-lifes, but not for mad1. This work is mostly technically sound and well performed.
Most of the results presented confirm previous studies, i.e. definition of mRNA and protein expression levels, subcellular localization, influence of codon optimalitu, effect(s) of GFP fusion, coupling of mRNA half-lives and protein stability to determine gene expression peaks etc. The authors do mention the literature and how the results they obtain are consistent with previous works. There are several potentially new findings (detailed below), but they are not developed to a point where new conclusions can be derived. At the present point, the parts of this work that are "finished" are mostly confirmatory and the newest findings would need a substantial amount of work before they are ready for publication.

Major points:

1- Figures 1, 2 and 3A-B, while relevant for following experiments, confirm published studies.

Parts of these figures indeed confirm prior findings but are important to characterize the specific strains used here (modified by scarless integration of GFP) and to set the stage for the downstream analyses.
Whether mad1 and mad2 mRNAs co-localize (Fig. 1F) has not been reported to our knowledge and is relevant to assembly of the Mad1/Mad2 complex.

2- Fig 3C-E and 5, while new for mad1-3 mRNAs, confirm what has been shown for other mRNAs.

While the correlation between codon usage and half-life is known, we think an interesting aspect of our study is that it highlights exceptions to the general rule. The mRNA half-lives of mad1 and ecm33 are short despite their moderate and optimal codon-usage (Fig EV4E), and one of the central points we make is that codon usage in mad1 has other nuanced functions outside of influencing mRNA half-life.

3- Fig 4. The impact of mRNA stabilization on protein level changes is very modest (unclear if it is statistically significant), suggesting a buffering mechanism. This is even more sticking if the one assumes the improving codon optimality should also increase translation. It would be more relevant to directly measure translation instead of steady state protein levels.

There is a clear difference between the codon-optimizations and ste13 deletion:
The increases in protein levels of Mad2 and Mad3 after codon optimization are statistically significant (Fig 4C,D).
The reduction in Mad1 protein levels after codon optimization was statistically significant, too (Fig 6C,D). (Note that reduced Mad1 mRNA and protein level after codon-optimization is one of the surprising findings in this study.)

Unlike for the codon-optimizations, we agree that buffering very likely occurs for *ste13* deletion, as was to be expected based on findings from budding yeast (e.g. Sun et al., 2013, Haimovich et al., 2013, Fischer et al., 2020, reviewed in Timmers and Tora, 2018). Despite an increase in mRNA half-life for mad2 and mad3 (Fig 3E), steady-state mRNA levels as well as protein levels are broadly unchanged (Fig 3D, 4B,C). The observations for mad1 are similar (Fig 5B,C, 6B,C).

We have opted not to additionally analyze translation after codon-optimization, since it is not the focus of the manuscript, and would not change the overall conclusions. The increases in Mad2 and Mad3 protein concentrations are slightly higher than the increases in mRNA levels, consistent with an increased translation rate.

4- Authors show that low codon optimality is required but not sufficient to regulate mRNA stability, and just cite a published work (Eser, 2016) for 3' UTR cis acting elements without further exploring the interplay between both events. Defining these elements and the interplay with codon optimality could have been a new and interesting mechanistic finding, but is not pursued.

All of the elements that have been bioinformatically defined in the cited study (and many of the ones characterized in other large-scale studies) are not understood on a mechanistic level. This will require substantial additional work and would in our opinion constitute another paper.

5- Authors combine codon optimality with stet13 depletion, with some apparently contradictory results. However the described effects are expected from previous works showing that codon optimality impacts on mRNA stability upstream of dhh1 (webster 2018) and that codon optimality and mRNA stability are linked through Not5 (buschauer 2020).

We think that correcting all mRNA measurements for cell size, along with additional experiments to increase statistical power, has eliminated what appeared to be contradictory results.

6- The authors attempt to link mad1-3 mRNAs stability and translation to dimer assembly by colocalization and coinmunoprecipitation experiments. They conclude that it may be relevant for Mad1. Although the co-IP experiments are consistent with this hypothesis, this approach is not sufficient to prove this point. A much more detailed study would be required.

We find it very difficult to interpret the co-IP experiments in any other way than that Mad1 homodimers assemble co-translationally. We show in two different strains that, in the presence of two different mad1 mRNA species, proteins of one type are much more likely to associate with the same type than the other type.

This conclusion is further corroborated by a recently published study on human translation (Bertolini et al., *Science* 2021), which demonstrates that mad1 mRNA is enriched in ribosome pairs (disomes) compared to monosomes. Mad1 was one of around 830 proteins that were identified as showing what the authors term “co-co assembly”.

As we explain above, we have now substantially strengthened the data (new Figs 7, EV6, and S6). We think this result is important to report, because previously published experiments need to be re-assessed based on this finding, as we describe in the Discussion.

Minor points

1- The effects of GFP limit the strength of some of the conclusions (i.e. line 100-103, fig. 1D and Fig. S1D), maybe they could try to fuse Mad1 to another fluorescent protein or non-fluorescent tag.

Tags are always a concern. However, any other tag would also come with drawbacks. For example, to compare mRNA levels of the wild-type and codon-optimized genes by FISH, they should carry the same tag, and such a tag should not be much smaller than GFP in order to allow for sufficient probes along the tag to yield a good signal-to-noise ratio. And any tag that is not fluorescent would not allow us to analyze protein concentrations in single cells. In our experience, GFP is one of the best-behaving (i.e. least functional interference, decent maturation time) out of the fluorescent-protein tags in *S. pombe*, and we have therefore refrained from creating strains with another tag.

2- In fig. 3D where the authors analyze the mRNA amount of mad2/3-GFP per cell, I would expect that the wt mad2/3-GFP would have increased mRNA/cell in the delta ste13 mutant, similar to that of the codon optimized Mad2/3-GFP, this is not shown in panel 3D, what is shown is the WT mRNA in WT cells.

We apologize for not having labeled the figure well enough. This figure has been revised in a major way – explained more below.

In the old figure, the gray curves were Mad2/Mad3-GFP (WT, not codon-optimized) in ste13+ background (from the data shown in Figure 1). They served as reference.
The histograms and fits (blue line) in the old figure showed:

- in row1: codon-optimized Mad2/Mad3-GFP in *ste13*$^{+}$
- in row 2: wild-type Mad2/Mad3-GFP in the *ste13D* background
- in row 3: codon-optimized Mad2/Mad3-GFP in the *ste13D* background

We have now revised the figure to make sure we appropriately control for cell size (previously only shown in the supplement, and with less reliable statistics (old S5C,D).

In the new Figure 3, we show mRNA numbers against cell length, along with regression lines and 95 % confidence intervals from the new generalized linear mixed model. The first row shows wild-type mad2-/mad3-GFP with gray regression line. The same regression line is shown in the subsequent rows as reference.
Row2 shows data from codon-optimized mad2-/mad3-GFP, row3 shows wild-type mad2-/mad3-GFP in ste13D. Steady-state mRNA levels are increased after codon-optimization, but not ste13 deletion (likely due to buffering).
Fig EV2 shows additional strains, and EV3 shows the same data, but now distinguishing between cytoplasmic and nuclear mRNA spots.

3- Mad2/3 protein concentrations decrease in the delta ste13 mutant (fig. 4B 4C, lines 208-210), do the tagged proteins, both codon optimized or not, also decrease levels in the ste13 mutants?

The new size-corrected analysis indicates that the mRNA levels of ymEGFP-tagged mad1, mad2, and mad3 in ste13D cells are similar to those of ste13+ cells, which is consistent with little change in the protein levels of (untagged) Mad1, Mad2, and Mad3.

4- Authors conclude that Mad1 IP precipitates the same amount of Mad1 and Mad1-GFP, I would say that precipitates more Mad1-GFP (line 255, fig. 6G).

Thank you for your careful attention to the wording. This has been rephrased: “In contrast, a Mad1 immunoprecipitation precipitated Mad1-GFP and Mad1 in approximately the same ratio in which they were present in the extract.”

Our statement was based on comparison with the input, where the strength of the signal for Mad1-GFP is also stronger than for untagged Mad1 (now Figure 7A, right side). Quantification of this blot (now at the bottom) finds a ratio of 3.0 in the input and of 1.9 in the Mad1 immunoprecipitation.

We ran a similar control for the IVT now shown in Fig 7D, where a Mad1 immunoprecipitation also yielded Mad1-flag-His and untagged Mad1 in roughly the same ratio (on average 0.31) as present in the input (0.28).

Referee #2 (Report for Author)

In this excellent study, Esposito and colleagues show that fission yeast cells maintain consistent levels of three checkpoint proteins: Mad1, Mad2, and Mad3/BubR1, by tuning mRNA and protein stability. The authors find that the low cell-to-cell variance in protein levels for the three genes arises through low mRNA stability coupled with high protein lifetime. Surprisingly, the authors find that non-optimal codon usage is responsible for the low stability of all three mRNA, but via two distinct mechanisms. In the case of Mad2 and Mad3, the mRNA molecules appear to be degraded via a known cellular pathway, whereas the low stability of Mad1 mRNA points to more complex and intriguing mechanism.

Overall, the manuscript presents well-designed experiments and thorough, thoughtful analyses and discussions of their outcomes. The findings presented shed light on a new aspect of the regulation of protein levels involved in spindle assembly checkpoint, and this regulation may well be shared by other proteins in fission yeast and other eukaryotes. Therefore, the manuscript will be of great interest for many cell biologists, and I fully support its publication. I do have a few minor questions and comments that the authors should address either by adding more discussion or by conducting new experiments and/or analyses.

1. The correlation between cell cycle, cell length, and transcript number per cell: Given that Mad1-3 are checkpoint signaling proteins, it is natural to expect that their expression is likely to be cell-cycle regulated. In fact, data exist documenting the cell-cycle dependence of the expression of these proteins in other eukaryotes. The authors should discuss the potential for cell-cycle regulation in the context of fission yeast. Mad1-Mad2 appear to have roles in nuclear import-export outside of mitosis in budding yeast (work from the Wozniak lab). Mad2 plays other significant roles in human cells (work from the Yu lab), whereas Mad3 is only thought to be important for checkpoint signaling. Therefore, there expression of the three proteins may be subject to cell-cycle-dependent regulation to differential degrees.

In fission yeast, at least under standard laboratory conditions, we and others have no evidence for cell cycle regulation of mad1, mad2, or mad3 (https://cyclebase.org/CyclebaseSearch). We have performed mRNA FISH for other genes that show cell cycle regulation, and their pattern is clearly distinct (see the example of cohesin rad21 below). For budding yeast, the situation is not entirely clear for mad3, but both mad1 and mad2 do not seem to be expressed in a cell cycle-regulated fashion either (https://cyclebase.org/CyclebaseSearch).

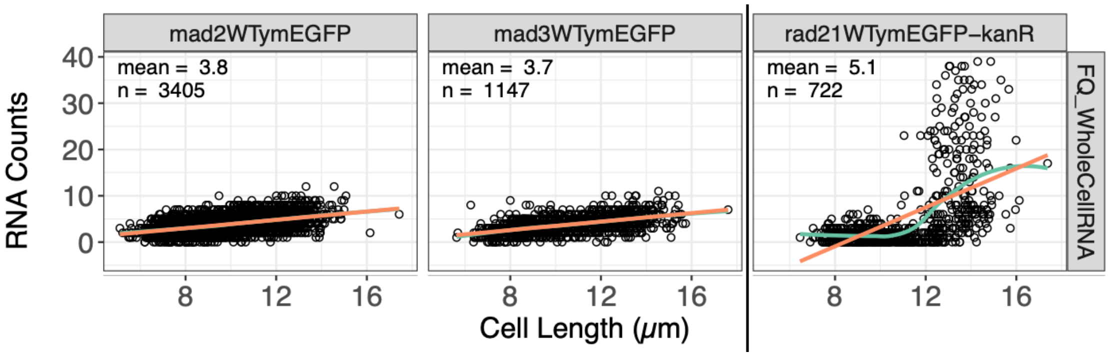

Interestingly, even in human cells, mad1 is very stably expressed and has been labeled as 'housekeeping'. Overall, in human data, mad1 (MAD1L1) and bub3 seem to be the most stably expressed among the SAC genes (FANTOM5, Nature 2014). Mad1 is also found among "low variable genes" in Arabidopsis thaliana (Cortijo/Locke, Mol Syst Biol 2019, PMID: 30679203).

On a related note, I think that there is an inconsistency between the strong correlation between cell

size and transcript number per cell (Fig. S1) and the Poisson fit with a single rate parameter shown in Fig. 1. Since the average transcript number per cell increases with cell size, doesn't this indicate that underlying synthesis and decay rates are changing? A possible explanation could be chromosome duplication following the S-phase. The authors can address this issue by suitably modifying the discussion.

The reviewer is correct that the increase in transcript number with cell size requires changes in the underlying synthesis or degradation rate. Previous studies, including some using fission yeast, have come to the conclusion that the transcription rate increases as cells grow and more RNA Polymerase II (in absolute numbers) is available (e.g. Zhurinsky et al., 2010, Kempe et al., 2014, Padovan-Merhar et al., 2015, Sun et al., 2020, Swaffer et al., bioRxiv 2021). This effect will indeed spread out the distribution when not correcting for cell size.

The fits in Fig. 1 are only meant to show that the distribution is roughly in the range that is consistent with a Poisson process. In fact, when correcting for cell size, the distribution is sub-Poissonian. We simply wanted to state that the observed distribution (although appearing broad) is definitely not “bursty” or super-Poissonian. We have modified the corresponding sub-title to avoid confusion.

While S-phase increases the gene number, the gene number does not seem the limiting factor for the expression of most genes in eukaryotes, and transcript number does not strongly increase in S-phase—presumably because the now higher number of genes still competes for the same pool of transcription machinery (e.g. Padovan-Merhar et al., 2015). In addition, fission yeast cells grown in nutrient-rich medium undergo an extremely rapid G1 phase, and S-phase takes place before cells septate. Hence, most cells in the population are post-S-phase.

2. Fig. 2A - it is not clear to me whether the CV was calculated based on the nuclear signal, the cytoplasmic signal, or the sum of the two for the three proteins. Does the value of the coefficient change based on which pool is considered? Additionally, both Mad1 and Mad2 localize strongly to the nuclear envelop, whereas Mad3 does not do so. Are there interesting correlations between the magnitudes of the nuclear, cytoplasmic, and nuclear membrane pools? This analysis is not essential, but it will be of interest to those who work in the checkpoint field.

We apologize for the incomplete description. The CV was calculated based on the total cellular signal. The nuclear quantifications are sensitive to small segmentation errors, in particular for Mad1 and Mad2, which enrich at the nuclear envelope. We therefore do not think that any strong conclusions can be drawn from the nuclear analysis.

3. Minor comment - I found is somewhat surprising that the Mad2 intensity in the nucleus appears to be ~ 1.5x lower than that of Mad1 (Fig. S3A). Mad2 must be in excess of Mad1 for mitotic checkpoint signaling according to the current understanding. A comment on this puzzling observation will be useful.

This question refers to what is now Fig S2A.

We were also slightly surprised by this result. Several factors likely play a role:
- Nuclear segmentation and quantification are error-prone, as mentioned in the previous point.
- Compared to traditional tagging, the levels of Mad1 after scarless tagging (used here) seem slightly higher, and those of Mad2 slightly lower (now Fig EV1A). Hence, there seems to be even less excess of Mad2 than we had previously estimated.
- Merely tagging mad1 with GFP increases the mRNA number (Fig 1E) and therefore presumably also the Mad1-GFP protein concentration. This means that (untagged) Mad1 levels in the Mad2-GFP strain are likely lower than those indicated by Mad1-GFP, ensuring an excess of Mad2.

- Since a large fraction of Mad2 is stably bound to Mad1, and Mad1 is strongly enriched in the nucleus, the nuclear concentrations of Mad1 and Mad2 will co-vary. In other words, cells at the lower/higher end of the Mad1 distribution will also be at the lower/higher end of the Mad2 distribution.

4. Another observation that deserves some discussion is that the amount of Mad2 is greater than that of Mad3 even though the protein and mRNA turn-over rates are comparable for the two proteins. Are the differences in these rates large enough to explain the significant difference in protein levels? The authors should be able to comment on this using their simulations.

While the concentration of Mad2 is indeed slightly higher than that of Mad3, it is not dramatically so (~110 % for whole-cell levels). Given the similar mRNA concentrations, this could be explained by a 10 % higher translation rate of mad2, for which there is some evidence from ribosome profiling (Rubio et al., 2020). It is also possible that the degradation rate is 10 % lower (or any mixture of the two). Given the very long protein half-life, a 10 % difference (e.g. 6 hours vs. 6.5 hours) may not be detectable.

5. Minor issue - In budding yeast, human cells, and in C. elegans, attaching a GFP to the N or the C-terminus is known to impair protein function. It is somewhat surprising to me that this is not the case in fission yeast, given that the homologs are structurally very similar. The authors do show that the SAC is functional as per the benomyl sensitivity assay, but this assay may not be sensitive enough to detect impaired Mad2 function. The authors may want to note this possibility in their discussion.

We agree that tags may subtly impair functions in ways that are not always easy to detect. We do not obtain strains with a functional SAC when tagging Mad1 or Mad2 N-terminally. For Mad1, this is consistent with not being able to express GFP alone from the mad1 locus (Fig S5A,B). In fact, we are puzzled that Mad1 can be tagged N-terminally in C. elegans and human cells. However, these were—to our knowledge— transgenes, and there are some indications that they are not fully functional (e.g. Moyle/Desai, JCB 2014).

It will be interesting to examine in the future whether the assembly pathway for the Mad1/Mad2 complex is the same between organisms (now mentioned in the Discussion). If not, this could explain why different organisms have a different tolerance for N-/C-terminal tags.

Unlike the observation with C. elegans Mad2-GFP (Lara-Gonzalez/Desai, Science 2021), we see S. pombe Mad2-GFP stably associating with Mad1 (e.g. Heinrich et al., NCB 2013, Fig. S6b, and Heinrich et al., EMBO reports 2014, Fig. 3H).

6. Co-IP of Mad1-GFP and Mad1: The possibility of co-translational folding and homodimerization of Mad1 and the role of the Mad1 transcript in promoting this is a really intriguing hypothesis. However, the evidence presented in its support (Fig. 6G-I) is not entirely convincing. Especially in the Mad1-GFP/Mad1 co-IP experiment, an appreciable amount of Mad1 does precipitate with Mad1-GFP. This is consistent with post-translational dimerization of the two proteins. The results of the Mad1-GFP/Mad1-strep experiment appear to be more in line with the co-translational dimerization hypothesis. The authors should comment on this discrepancy. This hypothesis is not the focus of the study, so the following experiment is optional. To rigorously test their hypothesis, the authors should consider an experiment based on in vitro translation. With in vitro translation, the authors should be able to titrate the mRNA relative to the ribosomal capacity and thus observe directly whether co-translational dimerization is significant on Mad1 transcripts.

The reviewer makes several excellent points, and we hope we have now addressed all of them (Fig 7, EV6, and S6).

(1) Relative ratio between the two forms immunoprecipitated or pulled down:

Thank you for the suggestion to add a quantitative analysis. The new Fig EV6 shows which ratio would be expected in IPs/pull-downs, given the ratio of isoforms in the extract, if hetero- and homodimers between the isoforms were equally likely. In all cases, the ratio that we observe exceeds the expected ratio, usually vastly. For example in Fig EV6B, the ratio in the extract is 2:1. Based on this, we would expect a ratio of 3:1 in a GFP-IP, and of 1.5:1 in the Strep pull-down, but instead we observe ratios of roughly 21:1 and 26:1. Other expected/observed ratios are shown in Fig 7A and 7C, and sometimes we were unable to determine the ratio, because a second band was not visible even in long exposures.

(2) Reproducibility in *in vitro* translation:

We have now performed *in vitro* transcription and translation reactions, and generally observe the same bias as in yeast extracts (Fig 7D, EV6C, S6). In the experiment shown in Fig 7D, combining a short flag-His tag and untagged Mad1, we would have expected a ratio of 1.3:1 in the His pull-down if heterodimers were equally likely, but instead we observe a ratio of around 35:1.

We are aware of one prior study that showed that heterodimers can be forced by high mRNA concentrations in an *in vitro* translation–presumably, because this would make it easier for two mRNA molecules to be in close enough vicinity for the protein chains to interact (Nicholls et al., JBC 2002, PMID: 11805092, https://doi.org/10.1074/jbc.M108815200). Despite going up to higher mRNA concentration than used in that study, we saw—if anything—a trend in the opposite direction (Fig EV6 and S6). Since the ratios of the isoforms, and the translation efficiency also changed as mRNA concentrations changed, we do not think we can draw any major conclusions from this result. It is important to emphasize that we still saw larger than expected ratios at all mRNA concentrations in the *in vitro* translation.

We had intended to use C-terminal fragments of Mad1 as a control where heterodimerization might become possible (Kim et al., 2012, PMID: 22493223, https://doi.org/10.1073/pnas.1118210109). However, in our hands, using *S. pombe* extracts or proteins, we still saw a bias towards pulling down the same isoform (Fig S6).

On another technical note, these experiments would in principle require demonstrating the absence of Mad1 monomers. For the full-length protein, monomers are extremely unlikely based on the structure (Sironi et al., 2002; Piano et al., 2021). Monomeric Mad1 would likely be unstable or aggregate. But it might be conceivable for the shorter C-terminal fragments. We attempted native gels to get at this, but this turned out to be technically too challenging (impossible?).

7. The role of transcriptional control on Mad2 and Mad3 levels: For the sake of completeness, the authors should note the role of Mad2 and Mad3 promoters in setting average transcript levels. This is important because the promoters must maintain a constant and low rate of transcription to enable the mRNA and protein turn-over rates to stabilize protein levels.
To highlight the role of transcriptional regulation, the authors should consider contrasting the result of Mad2 or Mad3 replacement with GFP with the surprising results of Mad1 replacement shown in Fig. 7.

Replacing mad2 or mad3 with GFP does not result in the same striking results as mad1 replacement, as is shown below, and now shown in Fig S5C, D.

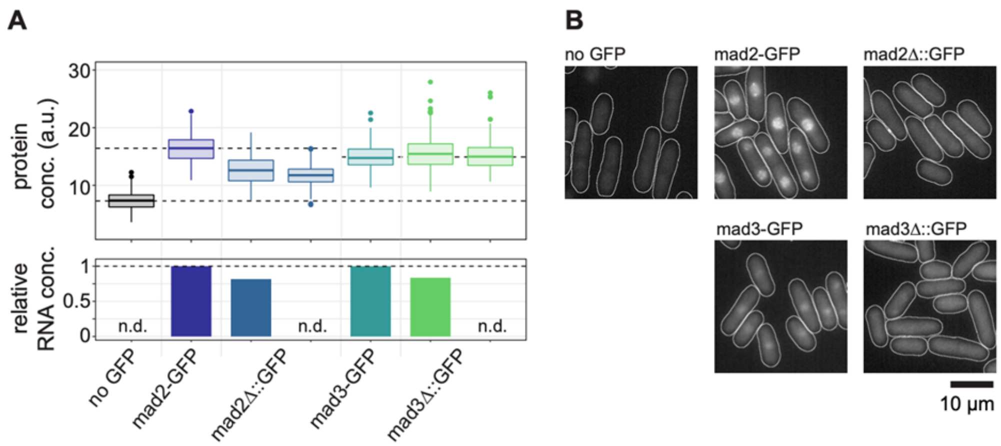

8. In some of the figures (e.g. Fig 6), information about the amount of protein loaded on the immunoblot is missing (e.g. Fig. 6B, G, I).

Thank you for pointing out the omission. For denatured protein extract, we now mention in the Methods section how much extract was loaded. For immunoprecipitations or pull-downs we now mention the input as percent of what was used for the immunoprecipitation or pull-down (each respective legend). Typically, around 30 µg were loaded as input.

9. A more detailed description of the overall calculation scheme behind the dynamic simulations presented in Fig. 2B would be useful. I may have overlooked this information, but I could only find a reference to the script used.

Thank you for pointing this out. The legend now refers to the Methods section, where this is described under “Gene expression models”. These data are now also clearly labeled as “simulation”, as suggested by reviewer #3.

Referee #3 (Report for Author)

In the study "Mitotic checkpoint gene expression is tuned by coding sequences" Esposito and colleagues study how the expression of core spindle assembly checkpoint proteins is controlled to ensure accurate protein stoichiometries. The authors discover that codon usage tunes the expression level of mad2 and mad3 but not mad1 in fission yeast. Notably, a high frequency of non-optimal codons leads to short half-life of mad2 and mad3 mRNAs, which in combination with long protein half-lives contributes to accurate protein stoichiometry. The authors show that cells deploy a different, albeit unresolved strategy to keep mad1 mRNAs short lived and protein levels well controlled.

How cells control stoichiometries of proteins is a poorly understood yet general problem in cell biology. Perhaps the most puzzling remains how the stoichiometries of different proteins that form complexes are regulated such that cells produce enough but not too much of each individual component of the complex. The SAC is a good example and an excellent starting point in addressing this important question as its components are well characterized, their interactions well documented, and consequences of perturbed homeostasis prominent and detrimental for cell survival. The reviewer, therefore, finds this study relevant, timely, and of broad interest.

The authors make novel claims and provide a comprehensive investigation showing that the expression of the SAC genes is controlled at the level of mRNA and protein stabilities. They show in computational simulations and in cells that a combination of short mRNA half-lives and long protein half-lives provides stable and accurate protein quantities. The short mRNA half-lives are attributed to high frequency of sub-optimal codons. This finding is further corroborated in codon-optimization experiments, where mRNA half-lives are prolonged resulting in increased protein concentrations. However, the comparison of mRNA half-life between unperturbed and codon-optimized strains remains somewhat confusing and would profit from revised data interpretation and presentation as discussed below.

Overall, the manuscript is clearly written and easy to follow, and the figures well-prepared, with a few minor comments discussed below. All experiments are well-documented, and appropriate controls are provided, but most experiments have been performed in duplicates rather than triplicates, and statistical analyses are missing throughout the manuscript as discussed below.

Even though it remains to be identified how Mad1 mRNA and protein levels are controlled, the reviewer fells that this work does represent an important step forward in deciphering how cells control protein quantities, in this case of the SAC complex via regulated mRNA and protein stability.

Major comments

1. The authors provide exciting new insights into how regulated mRNA and protein stabilities result in accurate stoichiometries of the core components of the SAC. However, the data are lacking statistical analyses throughout the manuscript, which the authors should provide in support of their conclusions.

We have now added statistical tests throughout:

- As explained above, we have in most cases opted to model the results using a generalized linear mixed model (GLMM) in order to be able to accommodate for different levels of repeats. Bootstrapping was used to obtain 95 % confidence intervals, which are now shown in the figures or tables.

In detail:

- Fig 1E: Difference examined by a GLMM, shown in Fig EV1E. Difference for mad1 (but not mad2) statistically significant.
- Fig 1F: Difference almost black-and-white and not examined statistically.
- Fig 2A: Nmt1 statistically different from SAC proteins by Wilcoxon rank sum test.
- Fig 3D and E: Differences examined by a GLMM. The change in steady-state mRNA numbers after codon-optimization, and the change in mRNA half-life after ste13 deletion were statistically significant. (For the half-life, see additional statistical analysis in Fig EV2C, which the new legend refers to).
- Fig 4C: Differences examined by t-tests (p values in legend).
- Fig 4D: Differences examined by a GLMM and statistically significant.
- Fig 5B and C: Differences examined by a GLMM. Neither the change in mad1 steady-state mRNA numbers after codon-optimization, nor the change in mad1 mRNA half-life after ste13 deletion were statistically significant. (For the half-life, see additional statistical analysis in Fig EV4E, which the new legend refers to).
- Fig 6C: Differences examined by t-tests (p values in legend).
- Fig 6D: Difference examined by GLMM and statistically significant.
- Fig 6F: Differences in each experiment examined by Kolmogorov-Smirnov test.
- Fig 7E,F: Almost perfect overlap between samples, not examined statistically.

2. Closely related to the previous comment, most experiments throughout this manuscript have been performed in two replicates, and a few rare ones even as just one. To strengthen their claims, and their statistical analyses, the authors should provide data from the third biological replicate.

We think our distinction between ‘biological’ (independent strain) and ‘experimental’ (repeat experiment) replicates may have led to some confusion.

The full information is now available in the source data. The Excel files contain one tab with the primary data, and—where useful—another “summary” sheet, which provides an easy overview on the number of biological and/or experimental replicates, and the number of cells in each replicate (See, for example, SourceData_Fig_1D_3D_5B_EV1C_EV2B_EV3_EV4A-C_EV5.xlsx). The figure legends have been revised as well.

To further corroborate our results, we have added additional repeat experiments for smRNA FISH in wild-type and ste13Δ cells.

3. One of the main conclusions of the work presented in this manuscript is that codon usage bias contributes to short half-lives of mad2 and mad3, but not mad1 mRNAs. To support this claim, the authors measure half-lives of these mRNAs. However, the measurements presented in Fig. 2D, Fig. 3E, and Fig. 5C do not seem to reflect the measurements reported in the related text. The authors should revise the text and figures to provide consistency. In addition, they should provide R values for the fits and perform statistical analyses to support their claims on the effect of codon optimization on mRNA stability.

The representation of these data has changed in the revised version.

In the previous version of the manuscript, we provided three different types of fits, which yielded qualitatively similar, but quantitatively slightly different results. This probably contributed to the confusion.

We are now fitting the data with a generalized linear mixed model (Fig 2D, 3E, 5C, EV4D), and show the 95 % confidence interval, as well as additional statistical analyses (Fig EV2C, EV4E). The change in half-life is statistically significant for mad2, mad3, and the ecm33 control. It is not statistically significant for mad1 and act1.

The Methods section has been revised accordingly.

4. In the abstract, as well as introduction (lines 76-78) and discussion lines (287-288) the authors present a tempting suggestion that the codon usage of SAC genes provides a feature that promotes proper function of the SAC. This claim, however, remains unaddressed in the manuscript. To support their statement, the authors should ideally test if the SAC works well in the strains where mad2 and mad3 sequences have been codon-optimized using the benomyl resistance assay (as in Fig. S1B). Adding another set of mutants that encode more suboptimal codons (the reverse of codon-optimization) and the analysis of their resistance to benomyl would further strengthen their claims. Alternatively, and at the very least, the authors should clearly indicate that these are speculations.

Since codon-optimization of mad2 and mad3 increases the protein concentrations, we do not expect impaired spindle assembly checkpoint (SAC) function.

The situation for Mad1 is more interesting: codon-optimization reduces the concentration, which on the one hand may impair the SAC due to reduced levels of Mad1 or possibly imperfect Mad1 complex formation or folding, but may also reinforce the SAC, since the pool of non-Mad1-bound Mad2 now likely is higher.

In the previous version of the manuscript, we showed that the SAC remains functional in strains with codon-optimized mad1 when all kinetochores are unattached, leading to a strong signal (now Fig S4E). To further explore this, we have now tested SAC function in codon-optimized mad1 strains using a weaker SAC activation by depleting the microtubule-interacting protein Alp7 (Fig 6F, S4F). We observe a reproducible tendency of cells expressing codon-optimized mad1 to delay in mitosis for shorter, although this did not reach statistical significance in each individual experiment.

Minor comments:

1. There seems to be an irrelevant self-citation in line 69 (Heinrich et al, 2013). This article seems inappropriately cited again in the results section (line 94) where it is referenced as a source of their previous mRNA number measurements using FISH, which doesn't appear to be present in the cited article.

We are sorry for appearing to have included a self-citation for no good reason, but we think this must be a misunderstanding. Line 69 is about the requirement for proper stoichiometry between Mad1 and Mad2. These data are in Fig. 4 of this article (https://www.nature.com/articles/ncb2864). The previous mRNA number measurements using FISH are in Fig. 3B of the same article.

2. The authors state that the efficiency of the mad2-specific FISH probe was lower than that one of the GFP-specific probe. However, it is unclear how they measure the probe efficiency to draw this conclusion. The authors should provide explanation either in the main text or in the materials and methods.

We are sorry for not having explained better. We have re-phrased the corresponding text.

This is based on counting mRNAs number for a GFP-fused gene, once with gene-specific probes and once with GFP probes (shown in Fig EV1D). The mean mRNA numbers for mad1-GFP are almost identical for the gene-specific (3.7) and the GFP probes (3.8). A slightly lower number for the gene-specific probe may be expected, since the majority of mRNA degradation in yeast seems to occur from the 5' end, and the GFP tag is at the 3' end.

For mad2, the gene-specific probes detected a lower mRNA number than the GFP-specific probes using the very same mad2-GFP strain (mean of 2.3 versus 3.6 mRNAs per cell; Fig EV1D). Given that—based on the mRNA half-life measurements—the turn-over of Mad2 does not seem to be dramatically faster than that of Mad1, we find this result most consistent with the gene-specific mad2 probes being less efficient in detecting mRNA (e.g. fewer probes binding and not always reaching the detection threshold).

The experiment in Fig 1E, which compares strains with untagged and tagged mad1 or mad2, only uses the gene-specific probes. Because the probes are the same, the relative difference between untagged and tagged should not be influenced by the lower efficiency of the mad2 probe.

We decided to show the probe efficiency (Fig EV1D) in order to explain why labeling of the same mad2-GFP strain can once yield an average of 3.8 mRNAs per cell (Fig 1D, with the GFP probe), and once an average of 2.3 mRNAs per cell (Fig 1E, with the gene-specific probe).

We hope the re-phrasing of the text (around line 107) has made this less confusing.

3. The authors should make sure to properly introduce the abbreviations throughout the manuscript.

We have carefully revised the text and we hopefully now introduce all abbreviations appropriately.

4. The reviewer recommends that the authors state explicitly in the manuscript text that the data presented in Fig. 2B are from a simulation. This figure is a little hard to read - for example, it is not evident what the x- and y- axes represent. Clarity would be improved if the authors provided a more elaborated caption within the figure legend.

Thank you for the suggestion. We have now labeled the simulation in Fig 2B, and the predictions in Fig 2C, S3B, and S3C as such, and have made efforts to improve both the labeling in the figure as well as the figure legend.

5. Fig. S7E is wrongly called in text.

Thank you. This has been corrected.

6. Measurements of the mad1 mRNA half-life reveal a much noisier pattern than the other two. Is it possible that this higher noise in mRNA levels is relevant for protein quantity control? Or is this noise a consequence of some technical challenges? The authors could touch on this briefly in their discussion.

Given the technical challenges of these experiments, we don't think we can conclude that there is a difference. The confidence intervals of mad1 (Fig 5C), mad2, and mad3 (Fig 3E) are all broad at the early timepoints. One of the ste13Δ experiments has an outlier at 25 min, but this is true for mad1, mad2, and mad3.

7. The materials and methods section contains information on Mad1-GFP localization on kinetochores, which appears irrelevant to this manuscript.

We think this may be a section that refers to data only shown in the supplementary material (now Fig S4D). We have kept it but could remove S4D and the corresponding methods part if the reviewer prefers.

1st Revision - Editorial Decision 20th May 2022

Thank you for submitting your revised manuscript and please excuse the delay in communicating this decision to you, which was due to repeatedly delayed referee reports. We have now however received comments from all of the initial referees (please see below). Referee #2 and #3 now explicitly support publication, while referee #1 is still not convinced by the degree of novelty provided by the findings overall. However, as no concerns questioning the findings per se remain, we have decided to proceed with the manuscript given the interest of the work for the cell cycle field, which had also been expressed by additional external experts we had contacted for their opinions. Referee #2 has two remaining suggestions, which you should consider and revise the text if appropriate. In addition, I would ask you to address a number of editorial issues that are listed in detail below in the final revised version of the manuscript. Please make all changes *only* to the document provided by our data editors (please see below for more information) and upload this as the main manuscript file when submitting the final revision.

Please contact me if you have further questions regarding the revision or any of the specific points listed below. Thank you again for giving us the chance to consider your manuscript for The EMBO Journal.

Referee #1:

The revised version of the manuscript solves some of the limitations of this study raised by the reviewers. However, I'm my opinion, the two main criticism are not addressed. First, the fact that a significant number of the results presented in this work confirm previous studies, (i.e. definition of mRNA and protein expression levels, subcellular localization, influence of codon optimality, effect(s) of GFP fusion, coupling of mRNA half-lives and protein stability to determine gene expression peaks etc.) by the authors and others. This is even acknowledged by the authors in the reply to points 1, 2, 5 and 6. Second, it is also well stablished that codon optimality is not the only mechanism regulating mRNA stability, a significant contribution will require elucidating these mechanism(s) for mad1-3 mRNAs, but the authors did not perform any experiment in this direction referring to future works. As it is this study does not represent a novel contribution to the level that should be expected for EMBO J.
Of the six raised main points, the response of the authors is just restating what was already included in the original manuscript.
Minor point 2, 3 and 4 are properly addressed/clarified.

Referee #2:

In this revised manuscript, Esposito et al. shed light on the codon-level regulation of the abundance for Mad1, Mad2, and Mad2, three spindle assembly checkpoint signaling proteins. They show that codon usage plays a vital role in regulating protein levels. In the case of Mad1, the authors show that disturbing codon usage degrades the fidelity of chromosome segregation during mitosis. They also provide convincing evidence for the co-translational homo-dimer formation for the Mad1 protein. Even though some of the reported findings, especially on the RNA biology side, confirm prior knowledge, applying this knowledge to mitotic proteins yields new insights. It raises new questions regarding the significance of Mad1 homodimer formation. The study was carefully conceived and executed, and the revisions further improved the quality of the manuscript. It is an important contribution to the mitosis field.

I have two suggestions for generalizing the significance of these findings, but whether to follow them is completely optional. Have the authors investigated the codon usage statistics for the three proteins in other organisms? The answer to this question (assuming that the Codon Stabilization Coefficient data are available) may help generalize these insights. Evidence for the co-translational dimerization of Mad1 also makes me wonder if other homodimers with large coiled-coil domains, e.g., kinesins, use the same mechanism.

Referee #3:

In the revised study "Mitotic checkpoint gene expression is tuned by coding sequences" Esposito and colleagues compellingly show that the expression of the spindle assembly checkpoint proteins Mad1, Mad2 and Mad3 in fission yeast is highly coordinated to provide steady and accurate protein levels. The authors show that codon usage and long protein half-lives tune the expression level of Mad2 and Mad3. The expression levels of Mad1 are regulated via a different pathway that involves co-translational assembly into homodimers. All these mechanisms are thought to provide optimal functioning of the spindle assembly checkpoint.

The revised version of the manuscript significantly strengthens the conclusions presented in the original version of the research article by Esposito and colleagues. The authors responded to all reviewers' comments and suggestions. Notably, the revised version of the manuscript includes 1) new experiments to support their findings on co-translational homodimerization of mad1, 2) improved statistical analyses and generalized linear mixed models, and 3) orthogonal approaches to test the functionality of the spindle assembly checkpoint in the various strains presented, all of which were identified as key weaknesses of the original manuscript.

The new data supporting co-translational assembly of mad1 not only broaden the scope of the article, but also reveal how cells utilize different mechanisms to control stoichiometries of different proteins that work in the same pathway. Such complex regulatory mechanisms are certainly not restricted only to the spindle assembly checkpoint complex. Rather, they are present much more broadly, yet remain poorly understood. Esposito and colleagues also revise their statistical and model fitting analyses throughout the revised manuscript, providing uniformity and completeness to their study. One of the principal claims of the original article was that the pathways that control protein quantities provide optimal functioning of the SAC. In the revised manuscript, the authors present additional evidence that the function of the SAC is reproducibly, albeit not statistically significantly impaired in cells with deregulated Mad1 levels.

Finally, the authors also addressed all the minor comments by providing either new experiments and analyses or a more detailed description and discussion of their findings.

In conclusion, the reviewer finds this study of high quality and worthy of publication.

Comment by reviewer # 2:

I have two suggestions for generalizing the significance of these findings, but whether to follow them is completely optional. Have the authors investigated the codon usage statistics for the three proteins in other organisms? The answer to this question (assuming that the Codon Stabilization Coefficient data are available) may help generalize these insights. Evidence for the co-translational dimerization of Mad1 also makes me wonder if other homodimers with large coiled-coil domains, e.g., kinesins, use the same mechanism.

These are great points. The second one was already covered in the text, but probably not explicitly enough. Bertolini, Bukau, Kramer, and colleagues performed a genome-wide study (Science, 2021, PMID: 33384371) for human protein homomers that may form co-translationally on the same mRNA (coined "co-co assembly"). They isolated ribosome disomes and analyzed their footprints on the mRNA. This approach found 829 human protein homomers that assemble co-translationally with high confidence. This list includes MAD1. Coiled-coil regions (such as present in MAD1) were the most prevalent driver of co-co assembly, and the list of co-co-assembling homomers also contains multiple kinesins and the nuclear basket protein TPR. We have rephrased the text in the Discussion part to make it clearer what has been found in this study.

Regarding codon-usage statistics in other organisms:

We are showing codon usage bias across Mad1 and Mad2 in S.c. and H.s. in Figure S7. We feel that any analysis beyond that is too uncertain due to the huge discrepancies in mRNA half-lives between studies (and even within studies). We have assembled all available, recent data in a separate figure to illustrate the point: generally, SAC gene CSCg values in S.c. are also low (indicating non-optimal codon usage), consistent with the data in S.p.; mRNA half-life data may suggest that the S.c. MAD2 mRNA half-life is longer than that for MAD1. This would reinforce the point, that these proteins, although tightly linked in their function, use different expression strategies, as we propose for S. pombe. However, the huge variability between studies makes this an unsound conclusion. The situation is yet worse for human cells. Any given SAC gene can differ for its relative mRNA half-life within the proteome by 75 percentiles or more. Presumably, this large variation is linked to the low expression of SAC genes (in S. pombe just a few mRNAs per cell), which make them challenging to measure in large-scale studies. Two human studies suggest that MAD1 may use more optimal codons (higher CSCg values) than MAD2. Again, this would reflect what we see in S. pombe (Fig 3B) and would also support different expression strategies. However, a third study (albeit using fewer data) does not support this point. Overall, therefore, we felt that any conclusions on the expression strategies in S.c. and H.s. would be premature, and that there is no benefit in showing these published data. We hope you concur.

hank you again for submitting the final revised version of your manuscript. As mentioned, you will hear from me again regarding the final textual edits of the transfer files, but for now I happy to inform you that we have formally accepted the study for publication in The EMBO Journal.

EMBO Press Author Checklist

Silke Hauf	
EMBOJ	
2021-107896R	

USEFUL LINKS FOR COMPLETING THIS FORM
The EMBO Journal - Author Guidelines
EMBO Reports - Author Guidelines
Molecular Systems Biology - Author Guidelines
EMBO Molecular Medicine - Author Guidelines

Reporting Checklist for Life Science Articles (updated January 2022)
This checklist is adapted from Materials Design Analysis Reporting (MDAR) Checklist for Authors. MDAR establishes a minimum set of requirements in transparent reporting in the life sciences (see Statement of Task: 10.31222/osf.io/9sm4x). Please follow the journal's guidelines in preparing your manuscript.
Please note that a copy of this checklist will be published alongside your article.

Abridged guidelines for figures
1. Data
The data shown in figures should satisfy the following conditions:
- the data were obtained and processed according to the field's best practice and are presented to reflect the results of the experiments in an accurate and unbiased manner.
- ideally, figure panels should include only measurements that are directly comparable to each other and obtained with the same assay.
- plots include clearly labeled error bars for independent experiments and sample sizes. Unless justified, error bars should not be shown for technical replicates.
- if n<5, the individual data points from each experiment should be plotted. Any statistical test employed should be justified.
- Source Data should be included to report the data underlying figures according to the guidelines set out in the authorship guidelines on Data Presentation.

2. Captions
Each figure caption should contain the following information, for each panel where they are relevant:
- a specification of the experimental system investigated (eg cell line, species name).
- the assay(s) and method(s) used to carry out the reported observations and measurements.
- an explicit mention of the biological and chemical entity(ies) that are being measured.
- an explicit mention of the biological and chemical entity(ies) that are altered/varied/perturbed in a controlled manner.
- the exact sample size (n) for each experimental group/condition, given as a number, not a range;
- a description of the sample collection allowing the reader to understand whether the samples represent technical or biological replicates (including how many animals, litters, cultures, etc.).
- a statement of how many times the experiment shown was independently replicated in the laboratory.
- definitions of statistical methods and measures:
  - common tests, such as t-test (please specify whether paired vs. unpaired), simple χ2 tests, Wilcoxon and Mann-Whitney tests, can be unambiguously identified by name only, but more complex techniques should be described in the methods section;
  - are tests one-sided or two-sided?
  - are there adjustments for multiple comparisons?
  - exact statistical test results, e.g., P values = x but not P values < x;
  - definition of 'center values' as median or average;
  - definition of error bars as s.d. or s.e.m.

Please complete ALL of the questions below.
Select "Not Applicable" only when the requested information is not relevant for your study.

Materials

Newly Created Materials	Information included in the manuscript?	In which section is the information available? (Reagents and Tools Table, Materials and Methods, Figures, Data Availability Section)
New materials and reagents need to be available; do any restrictions apply?	Yes	Data Availability Section

Antibodies	Information included in the manuscript?	In which section is the information available? (Reagents and Tools Table, Materials and Methods, Figures, Data Availability Section)
For **antibodies** provide the following information:<br>- Commercial antibodies: RRID (if possible) or supplier name, catalogue number and or/clone number<br>- Non-commercial: RRID or citation	Yes	Reagents and Tools Table

DNA and RNA sequences	Information included in the manuscript?	In which section is the information available? (Reagents and Tools Table, Materials and Methods, Figures, Data Availability Section)
**Short novel DNA or RNA including primers, probes**: provide the sequences.	Yes	Table S2 - S6

Cell materials	Information included in the manuscript?	In which section is the information available? (Reagents and Tools Table, Materials and Methods, Figures, Data Availability Section)
**Cell lines:** Provide species information, strain. Provide accession number in repository **OR** supplier name, catalog number, clone number, and/**OR** RRID.	Not Applicable	
**Primary cultures:** Provide species, strain, sex of origin, genetic modification status.	Not Applicable	
Report if the cell lines were recently **authenticated** (e.g., by STR profiling) and tested for mycoplasma contamination.	Not Applicable	

Experimental animals	Information included in the manuscript?	In which section is the information available? (Reagents and Tools Table, Materials and Methods, Figures, Data Availability Section)
**Laboratory animals or Model organisms:** Provide species, strain, sex, age, genetic modification status. Provide accession number in repository **OR** supplier name, catalog number, clone number, **OR** RRID.	Not Applicable	
**Animal observed in or captured from the field:** Provide species, sex, and age where possible.	Not Applicable	
Please detail **housing and husbandry conditions**.	Not Applicable	

Plants and microbes	Information included in the manuscript?	In which section is the information available? (Reagents and Tools Table, Materials and Methods, Figures, Data Availability Section)
**Plants:** provide species and strain, ecotype and cultivar where relevant, unique accession number if available, and source (including location for collected wild specimens).	Not Applicable	
**Microbes:** provide species and strain, unique accession number if available, and source.	Yes	Table S1

Human research participants	Information included in the manuscript?	In which section is the information available? (Reagents and Tools Table, Materials and Methods, Figures, Data Availability Section)
If collected and within the bounds of privacy constraints report on age, sex and gender or ethnicity for all study participants.	Not Applicable	

Core facilities	Information included in the manuscript?	In which section is the information available? (Reagents and Tools Table, Materials and Methods, Figures, Data Availability Section)
If your work benefited from core facilities, was their service mentioned in the acknowledgments section?	Not Applicable	

Design

Study protocol	Information included in the manuscript?	In which section is the information available? (Reagents and Tools Table, Materials and Methods, Figures, Data Availability Section)
If study protocol has been **pre-registered, provide DOI in the manuscript**. For clinical trials, provide the trial registration number **OR** cite DOI.	Not Applicable	
Report the **clinical trial registration number** (at ClinicalTrials.gov or equivalent), where applicable.	Not Applicable	

Laboratory protocol	Information included in the manuscript?	In which section is the information available? (Reagents and Tools Table, Materials and Methods, Figures, Data Availability Section)
Provide DOI OR other citation details if **external detailed step-by-step protocols** are available.	Not Applicable	

Experimental study design and statistics	Information included in the manuscript?	In which section is the information available? (Reagents and Tools Table, Materials and Methods, Figures, Data Availability Section)
Include a statement about **sample size** estimate even if no statistical methods were used.	Yes	Methods and Protocols / Statistical tests
Were any steps taken to minimize the effects of subjective bias when allocating animals/samples to treatment (e.g. **randomization procedure**)? If yes, have they been described?	Not Applicable	
Include a statement about **blinding** even if no blinding was done.	Yes	Methods and Protocols / Statistical tests
Describe **inclusion/exclusion criteria** if samples or animals were excluded from the analysis. Were the criteria pre-established?<br><br>If sample or data points were omitted from analysis, report if this was due to attrition or intentional exclusion and provide justification.	Yes	Criteria were not pre-established, but are described in the Methods and Protocols section
For every figure, are **statistical tests** justified as appropriate? Do the data meet the assumptions of the tests (e.g., normal distribution)? Describe any methods used to assess it. Is there an estimate of variation within each group of data? Is the variance similar between the groups that are being statistically compared?	Yes	

Sample definition and in-laboratory replication	Information included in the manuscript?	In which section is the information available? (Reagents and Tools Table, Materials and Methods, Figures, Data Availability Section)
In the figure legends: state number of times the experiment was **replicated** in laboratory.	Yes	Legends and source data
In the figure legends: define whether data describe **technical or biological replicates**.	Yes	Legends and source data, Methods and Protocols

Ethics

Ethics	Information included in the manuscript?	In which section is the information available? (Reagents and Tools Table, Materials and Methods, Figures, Data Availability Section)
Studies involving **human participants**: State details of **authority granting ethics approval** (IRB or equivalent committee(s), provide reference number for approval.	Not Applicable	
Studies involving **human participants**: Include a statement confirming that **informed consent** was obtained from all subjects and that the experiments conformed to the principles set out in the WMA Declaration of Helsinki and the Department of Health and Human Services Belmont Report.	Not Applicable	
Studies involving **human participants:** For publication of **patient photos**, include a statement confirming that consent to publish was obtained.	Not Applicable	
Studies involving experimental **animals**: State details of **authority granting ethics approva**l (IRB or equivalent committee(s), provide reference number for approval. Include a statement of compliance with ethical regulations.	Not Applicable	
Studies involving **specimen and field samples:** State if relevant **permits** obtained, provide details of authority approving study; if none were required, explain why.	Not Applicable	

Dual Use Research of Concern (DURC)	Information included in the manuscript?	In which section is the information available? (Reagents and Tools Table, Materials and Methods, Figures, Data Availability Section)
Could your study fall under dual use research restrictions? Please check biosecurity documents and list of **select agents and toxins** (CDC): https://www.selectagents.gov/sat/list.htm	Not Applicable	
If you used a select agent, is the security level of the lab appropriate and reported in the manuscript?	Not Applicable	
If a study is subject to dual use research of concern regulations, is the name of the **authority granting approval and reference number** for the regulatory approval provided in the manuscript?	Not Applicable	

Reporting

The MDAR framework recommends adoption of discipline-specific guidelines, established and endorsed through community initiatives. Journals have their own policy about requiring specific guidelines and recommendations to complement MDAR.

Adherence to community standards	Information included in the manuscript?	In which section is the information available? (Reagents and Tools Table, Materials and Methods, Figures, Data Availability Section)
State if relevant guidelines or checklists (e.g., **ICMJE, MIBBI, ARRIVE, PRISMA**) have been followed or provided.	Not Applicable	
For **tumor marker prognostic studies,** we recommend that you follow the **REMARK** reporting guidelines (see link list at top right). See author guidelines, under 'Reporting Guidelines'. Please confirm you have followed these guidelines.	Not Applicable	
For **phase II and III randomized controlled trials**, please refer to the **CONSORT** flow diagram (see link list at top right) and submit the CONSORT checklist (see link list at top right) with your submission. See author guidelines, under 'Reporting Guidelines'. Please confirm you have submitted this list.	Not Applicable	

Data Availability

Data availability	Information included in the manuscript?	In which section is the information available? (Reagents and Tools Table, Materials and Methods, Figures, Data Availability Section)
Have **primary datasets** been deposited according to the journal's guidelines (see 'Data Deposition' section) and the respective accession numbers provided in the Data Availability Section?	Not Applicable	
Were **human clinical and genomic datasets** deposited in a public access-controlled repository in accordance to ethical obligations to the patients and to the applicable consent agreement?	Not Applicable	
Are **computational models** that are central and integral to a study available without restrictions in a machine-readable form? Were the relevant accession numbers or links provided?	Not Applicable	
If publicly available data were reused, provide the respective **data citations in the reference list**.	Yes	References, Text, and Methods and Protocols
