# Supplementary material for: Mitotic checkpoint gene expression is tuned by codon usage bias
Source: EMBO J. 2022 Jul 11;41(15):e107896. doi: 10.15252/embj.2021107896 (PMC9340482; doi:10.15252/embj.2021107896)

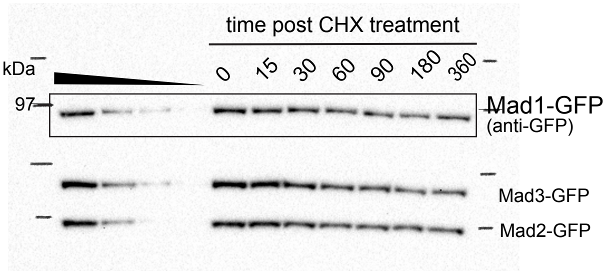

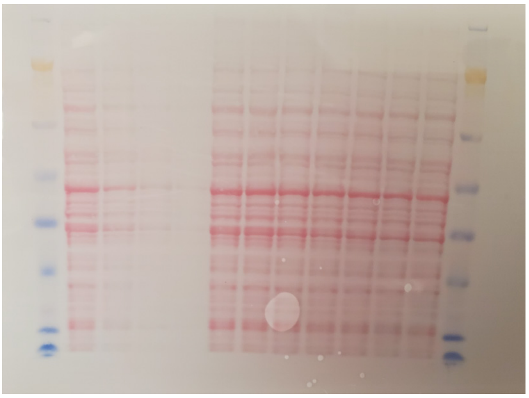

Membrane cut for different antibody incubations

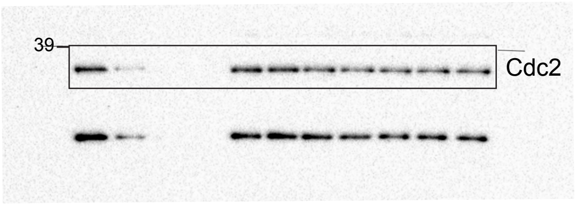

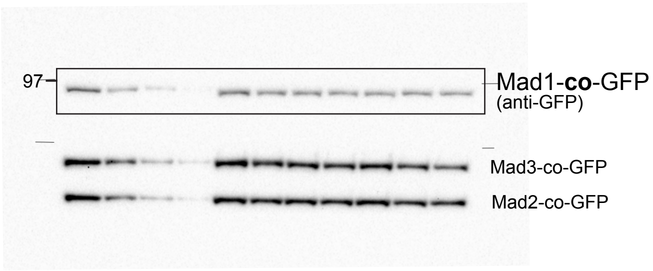

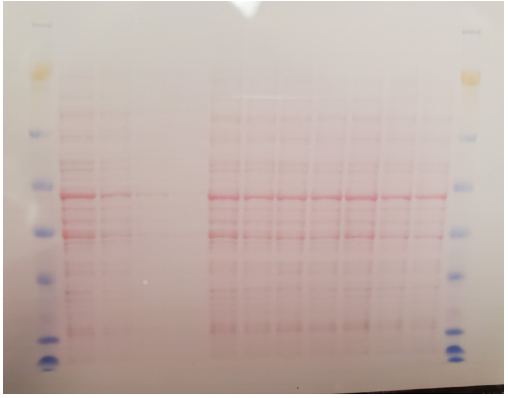

Membrane cut for different antibody incubations

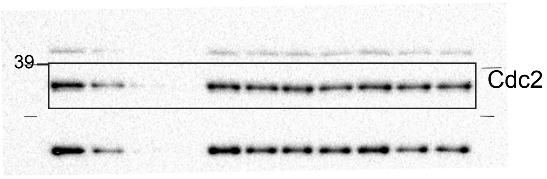

Supplement: Supplementary file 3 — Source Data for Expanded View and Appendix [file EMBJ-41-e107896-s006.zip › EMBJ_2363_source_data_Appendix/source_data_Appendix/source_data_figS4.zip!/source_data_figS4/SourceData_Fig_S4A_uncropped.pdf]
