# Supplementary material for: Mitotic checkpoint gene expression is tuned by codon usage bias
Source: EMBO J. 2022 Jul 11;41(15):e107896. doi: 10.15252/embj.2021107896 (PMC9340482; doi:10.15252/embj.2021107896)

m-anti-GFP

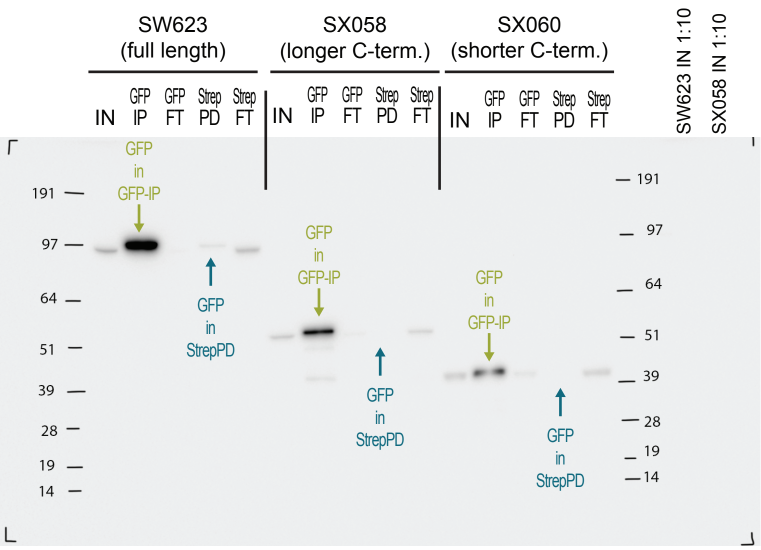

20 sec exposure

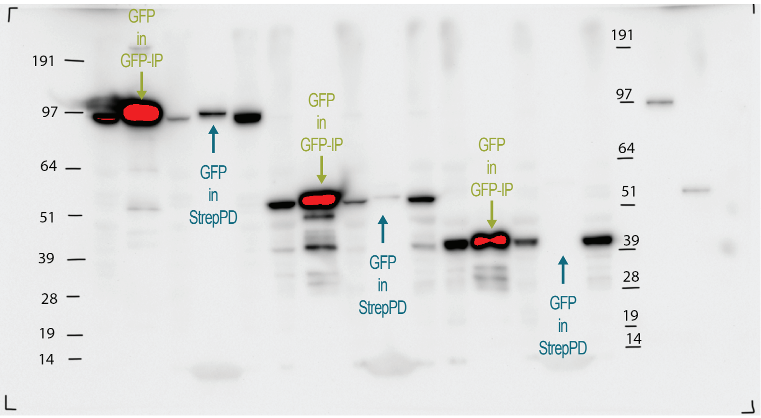

30 min exposure

rb-anti-Strep

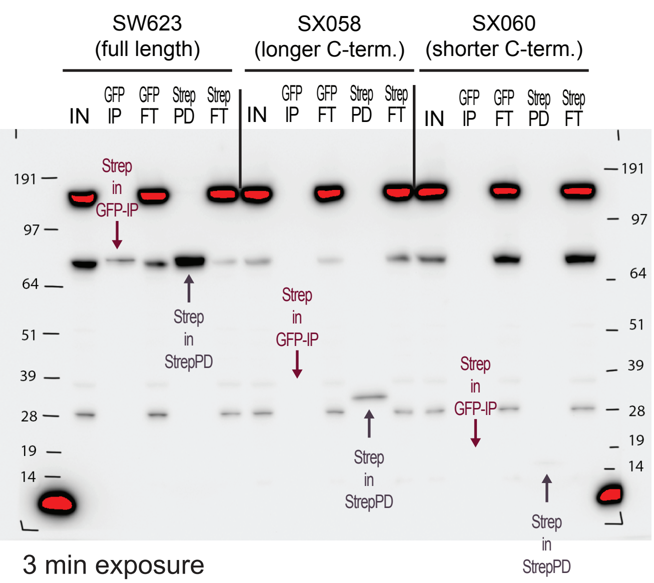

3 min exposure

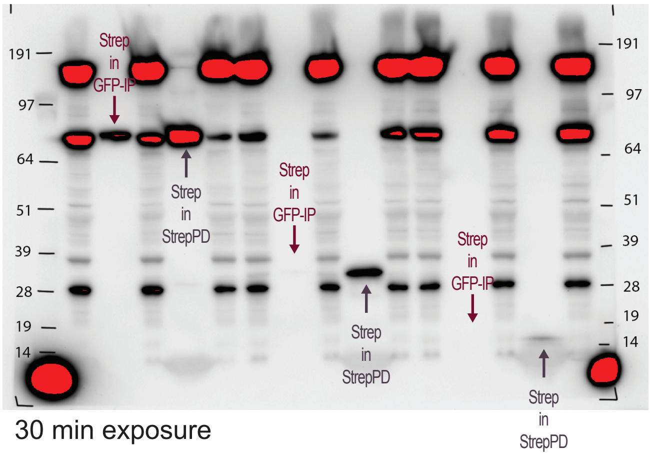

30 min exposure

full length Mad1-GFP – 105 kDa
full length Mad1-strep – 78 kDa
longer Mad1-GFP fragment – 56 kDa
longer Mad1-strep fragment – 29 kDa
shorter Mad1-GFP fragment – 41 kDa
shorter Mad1-Strep fragment – 17 kDa
Band from Tactin XT - 13.5 kDa (denaturing elution)

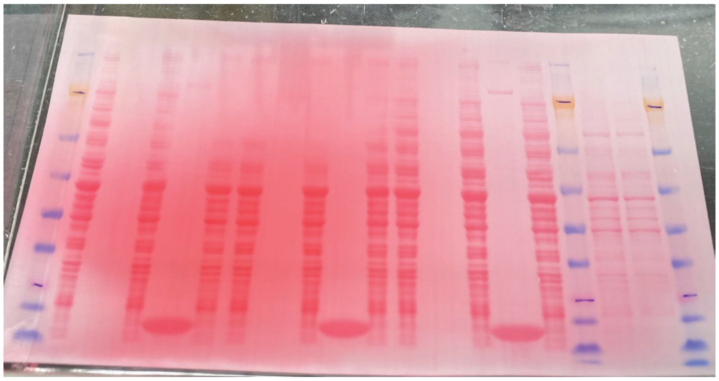

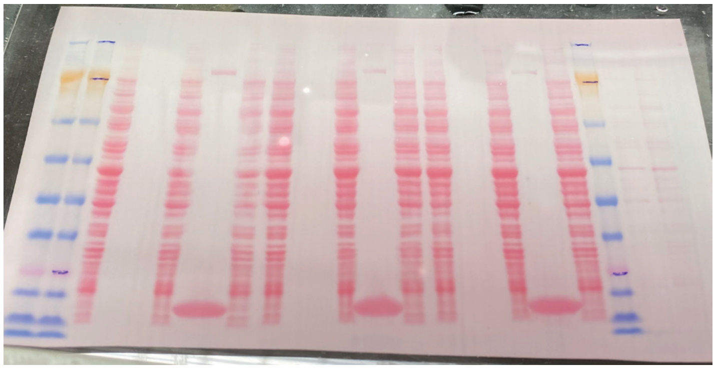

Supplement: Supplementary file 3 — Source Data for Expanded View and Appendix [file EMBJ-41-e107896-s006.zip › EMBJ_2363_source_data_Appendix/source_data_Appendix/source_data_figS6.zip!/source_data_figS6/SourceData_Fig_S6A_uncropped.pdf]
