# Supplementary material for: Mitotic checkpoint gene expression is tuned by codon usage bias
Source: EMBO J. 2022 Jul 11;41(15):e107896. doi: 10.15252/embj.2021107896 (PMC9340482; doi:10.15252/embj.2021107896)
Supplement: Supplementary file 3 — Source Data for Expanded View and Appendix [file EMBJ-41-e107896-s006.zip › EMBJ_2363_source_data_Appendix/source_data_Appendix/source_data_figS6.zip!/source_data_figS6/SourceData_Fig_S6B_uncropped.pdf]

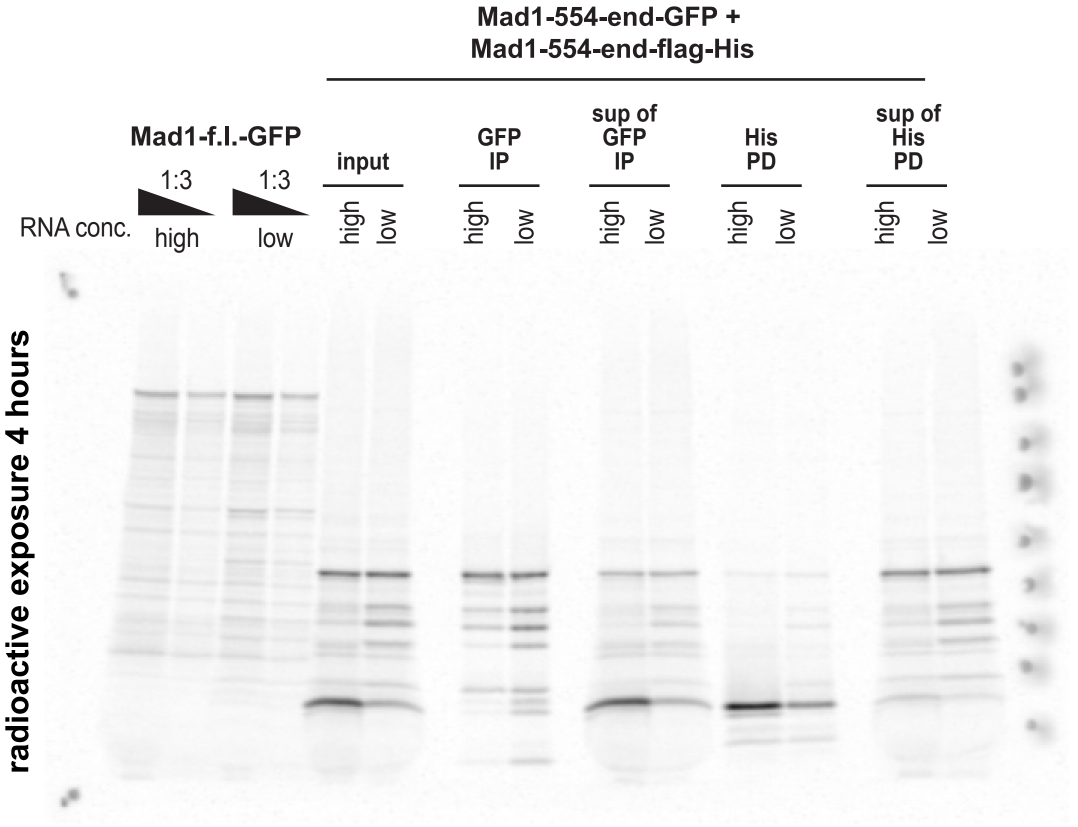
Mad1-554-end-GFP +
Mad1-554-end-flag-His
Mad1-f.l.-GFP
1:3
1:3
RNA conc.
high
low
input
GFP IP
sup of GFP IP
His PD
sup of His PD
high
low
radioactive exposure 4 hours

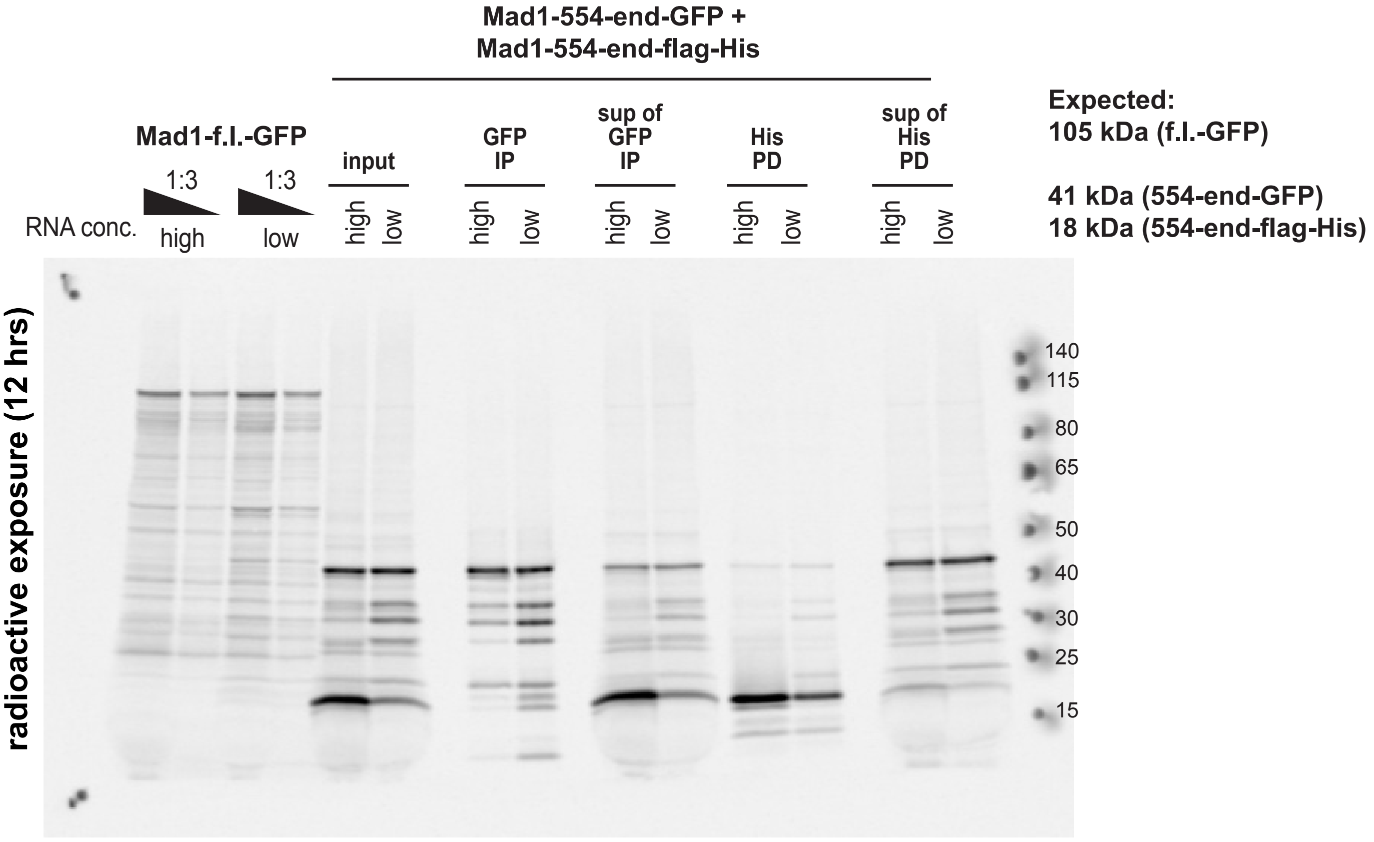
Mad1-554-end-GFP +
Mad1-554-end-flag-His
Mad1-f.l.-GFP
1:3
1:3
RNA conc.
high
low
input
GFP IP
sup of GFP IP
His PD
sup of His PD
high
low
radioactive exposure (12 hrs)
140
115
80
65
50
40
30
25
15
Expected:
105 kDa (f.l.-GFP)
41 kDa (554-end-GFP)
18 kDa (554-end-flag-His)
