# Supplementary material for: Mitotic checkpoint gene expression is tuned by codon usage bias
Source: EMBO J. 2022 Jul 11;41(15):e107896. doi: 10.15252/embj.2021107896 (PMC9340482; doi:10.15252/embj.2021107896)

Ponceau

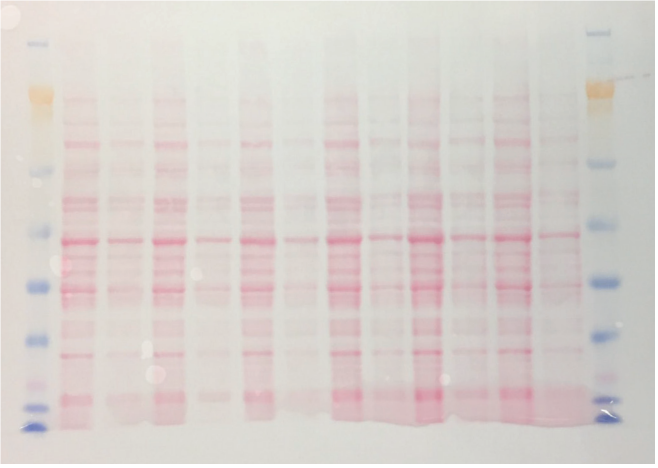

Membrane cut for different antibody incubations

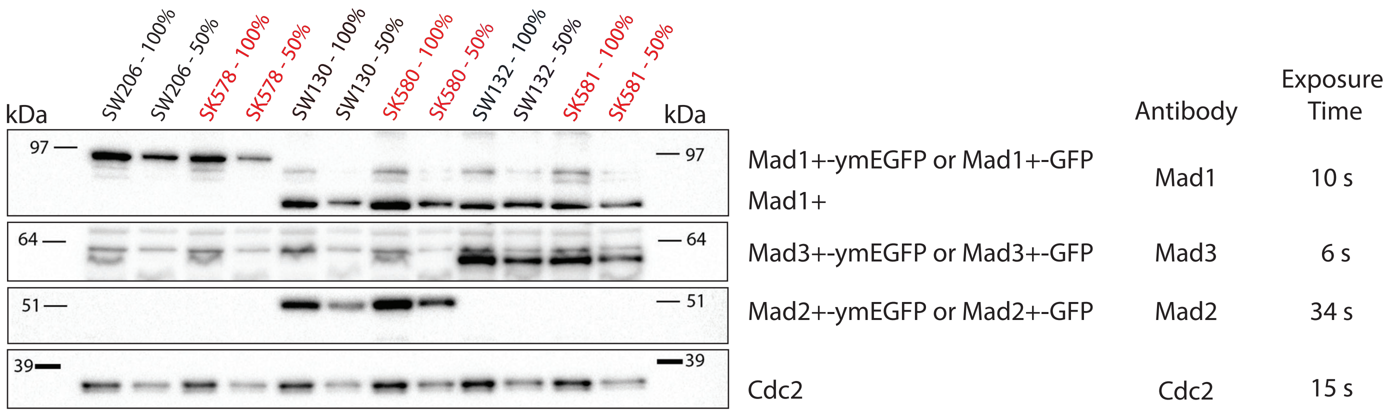

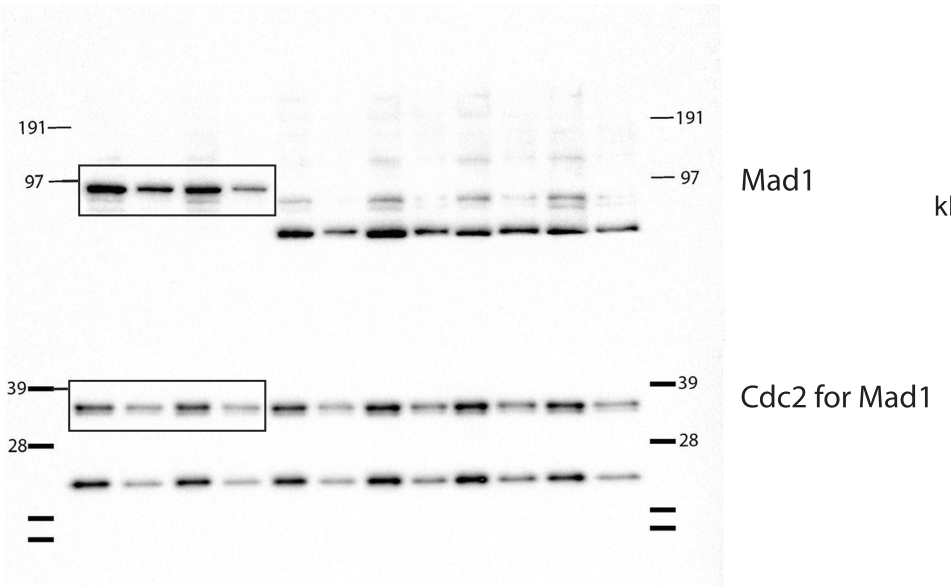

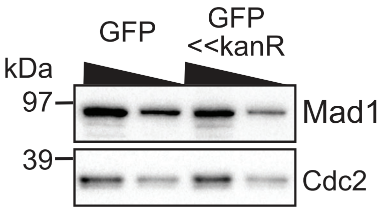

Ponceau

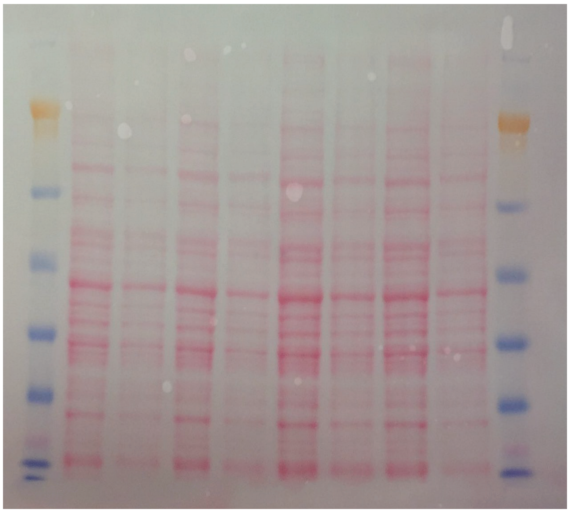

Membrane cut for different antibody incubations

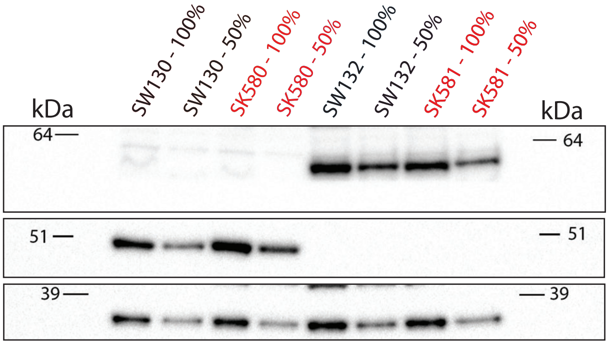

	Antibody	Exposure Time
Mad3+-ymEGFP or Mad3+-GFP	Mad3	5 s
Mad2+-ymEGFP or Mad2+-GFP	Mad2	46 s
Cdc2	Cdc2	27 s

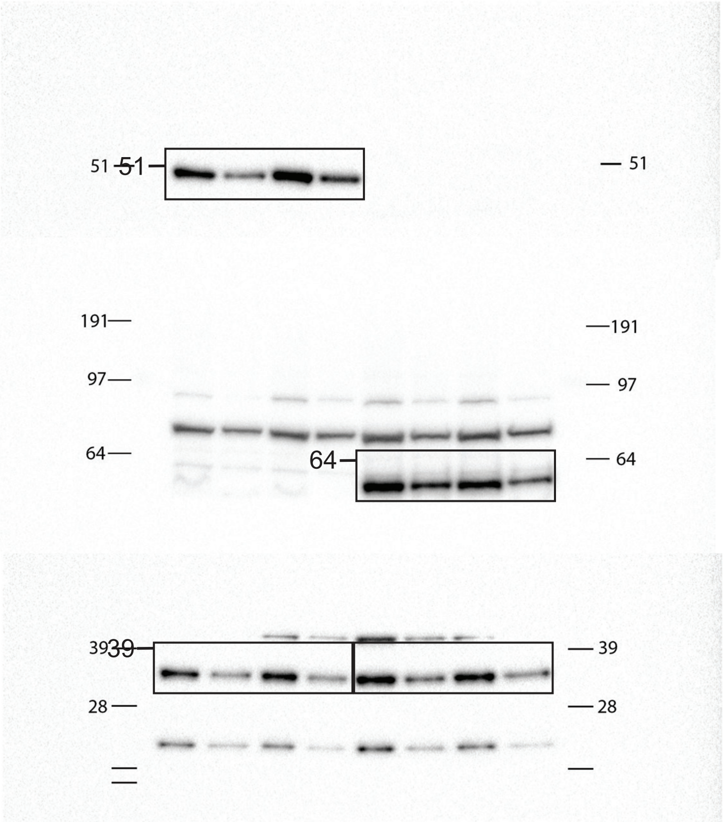

Mad2

Mad2

Cdc2 for Mad2 and Mad3

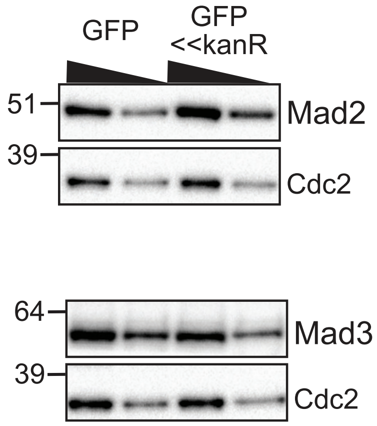

Supplement: Supplementary file 3 — Source Data for Expanded View and Appendix [file EMBJ-41-e107896-s006.zip › EMBJ_2363_source_data_EVfigures/source_data_EVfigures/source_data_figEV1.zip!/source_data_figEV1/SourceData_Fig_EV1A_uncropped.pdf]
