# Supplementary material for: Mitotic checkpoint gene expression is tuned by codon usage bias
Source: EMBO J. 2022 Jul 11;41(15):e107896. doi: 10.15252/embj.2021107896 (PMC9340482; doi:10.15252/embj.2021107896)
Supplement: Supplementary file 3 — Source Data for Expanded View and Appendix [file EMBJ-41-e107896-s006.zip › EMBJ_2363_source_data_EVfigures/source_data_EVfigures/source_data_figEV6.zip!/source_data_figEV6/SourceData_Fig_EV6B_uncropped.pdf]

rb anti-Mad1
(after mouse anti-GFP and quenching)

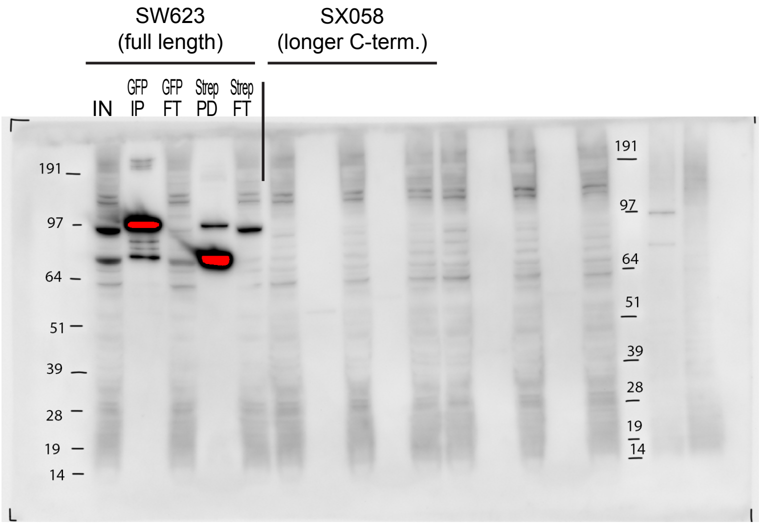

3 min exposure

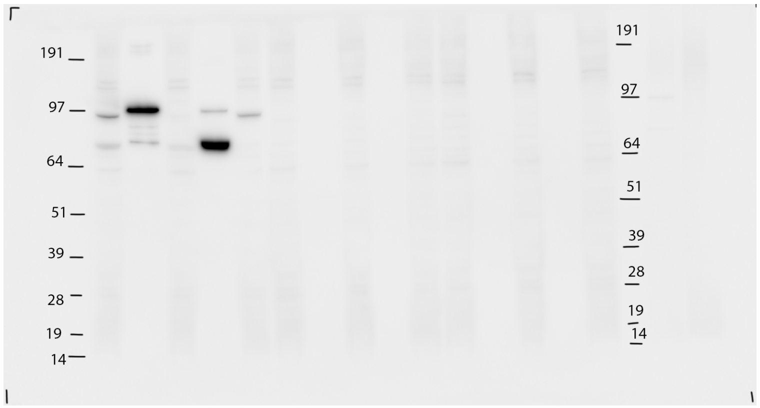

20 sec exposure
